# Reviewing and screening ionic liquids and deep eutectic solvents for effective CO_2_ capture

**DOI:** 10.3389/fchem.2022.951951

**Published:** 2022-08-10

**Authors:** Sahar Foorginezhad, Gangqiang Yu, Xiaoyan Ji

**Affiliations:** ^1^ Energy Science/Energy Engineering, Department of Engineering Sciences and Mathematics, Luleå University of Technology, Luleå, Sweden; ^2^ Faculty of Environment and Life, Beijing University of Technology, Beijing, China

**Keywords:** ionic liquid, deep eutectic solvents, CO_2_ capture, COSMO-RS, green absorbents

## Abstract

CO_2_ capture is essential for both mitigating CO_2_ emissions and purifying/conditioning gases for fuel and chemical production. To further improve the process performance with low environmental impacts, different strategies have been proposed, where developing liquid green absorbent for capturing CO_2_ is one of the effective options. Ionic liquids (IL)/deep eutectic solvents (DES) have recently emerged as green absorbents with unique properties, especially DESs also benefit from facile synthesis, low toxicity, and high biodegradability. To promote their development, this work summarized the recent research progress on ILs/DESs developed for CO_2_ capture from the aspects of those physical- and chemical-based, and COSMO-RS was combined to predict the properties that are unavailable from published articles in order to evaluate their performance based on the key properties for different IL/DES-based technologies. Finally, top 10 ILs/DESs were listed based on the corresponding criteria. The shared information will provide insight into screening and further developing IL/DES-based technologies for CO_2_ capture.

## 1 Introduction

Nowadays, the energy supply highly depends on carbonaceous fuels, especially fossil fuels, including petroleum, coal, and conventional/unconventional natural gas (Wang and Song, 2020). The combustion of these fuels in mobile energy systems, power plants, and industrial factories results in the emission of greenhouse gases (Wang and Song, 2020). CO_2_ is considered as the most important anthropogenic greenhouse gas, leading to ocean acidification, global warming, land desertification, and rising sea level (Sanz-Perez et al., 2016). Also, it is delineated that the concentration of CO_2_ in the atmosphere has significantly soared from 340 to 408 ppm in the 1980–2019 time period (Ghanbari et al., 2020). Thus, CO_2_ capture from flue gases has gained considerable attention among industrial and academic authorities.

On the other hand, CO_2_ separation is needed to produce renewable fuels to reduce CO_2_ emissions from the transportation sector. For instance, biomass gasification offers a possibility to produce more carbon-neutral transportation fuels, such as methanol, hydrogen, and synthetic hydrocarbons, where CO_2_ removal is always required (Shahbaz et al., 2021). In the production of synthetic hydrocarbons with the Fischer-Tropsch technology, the inert CO_2_ is removed to increase the efficiency and selectivity of C_5+_; in the methanol production, CO_2_ removal is made to obtain a favorable ratio of the gas mixture to increase the production yield; while in the hydrogen production, CO_2_ separation is needed to purify hydrogen. In parallel, bio-methane production and its usage as vehicle fuels provide an alternative and important pathway to mitigate CO_2_ emissions from the transportation sector. In general, the raw biogas generated in the anaerobic digestion contains CO_2_ of around 30–50 vol%. To be used as vehicle fuels, CO_2_ removal from the raw biogas (i.e., biogas upgrading) is needed to lower the CO_2_ content to 2–6 vol% (Ahmed et al., 2021). Besides, bio-carbon capture, utilization and/or storage (bio-CCUS) from the bioenergy sector is of considerable interest towards negative carbon emissions, further highlighting the importance of CO_2_ capture/separation.

Currently, the available technologies for CO_2_ separation mainly include absorption, adsorption, membrane, and cryogenic separation, and they are very costly ($50–100/ton-CO_2_) combined with other challenges (Mondal et al., 2012). For example, the aqueous amine-based technology (absorption) is energy-intensive, and volatility (secondary pollution), degradation, and corrosion are other deficiencies; the selectivity of the membrane is often lost in the presence of H_2_; adsorption is energy consuming and usually requires large-scale operations; the Rectisol process needs high power for refrigeration. Further development is needed to decrease the cost of CO_2_ separation with marginal or no environmental influence, where solvent-based technologies have been suggested as the most suitable candidates considering the tolerance for the impurities from the gas streams.

To develop solvent-based technology for CO_2_ separation, different kinds of absorbents have been proposed, where ionic liquids (ILs) have drawn significant attention because 1) they are nonvolatile, nonflammable, environmentally benign, and thermal stable; 2) their chemical and physical properties are tunable, making it possible to design a specific IL for a specific CO_2_ capture process; and 3) relatively high CO_2_ solubility and selectivity as well as relatively low energy-demand for solvent regeneration. Additionally, a new class of ILs or IL analogous, i.e., deep eutectic solvents (DESs), share similar properties to the conventional ILs together with other additional appealing properties, such as their simple preparation processes, low cost, biodegradability, and environmentally friendly materials used in synthesis (Sarmad et al., 2017a).

Owing to the unique properties of both ILs and DESs, intensive work has been conducted, and results have been reported all the time, calling for a new survey of the latest research. Also, the extensive work imposes the challenge to screen and suggest proper ILs and DESs for a specific purpose, which is related to the compositions and conditions of the streams needed for CO_2_ separation as well as other available resources, such as waste-heat or the availability of steam, etc. To the best of our knowledge, it is still unclear which ILs and DESs within those developed are more desirable for a specific IL/DES-based technology. Indeed, process simulation and evaluation are conducted, but just after a very brief and quantitative comparison of some properties. Especially in some works, the important properties, e.g., viscosity and/or selectivity, are not included or concerned sufficiently, making it difficult to convince the reliability of the simulation results (Hospital-Benito et al., 2021). All these issues call for a proper screen, selection, and suggestion of promising ILs and DESs for further study and development.

To screen ILs/DESs, properties are needed, which can be determined experimentally or predicted theoretically. Theoretical models have been developed to predict properties of ILs/DESs qualitatively or quantitively, and the models include Quantitative Structure Property Relationship (QSPR) method (Eike et al., 2004), regular solution theory (Kilaru et al., 2008), Molecular dynamics (Kerlé et al., 2009), Monte Carlo (Shah and Maginn, 2005), group contribution method (Kim et al., 2005; Zhang et al., 2021b; Zhou et al., 2021), Conductor-like Screening Model for Real Solvents (COSMO-RS) method (Liu et al., 2021), statistical associating fluid theory (SAFT)-based equation of state (Ji et al., 2012; Ji and Zhu, 2013; Ji et al., 2014; Shen et al., 2015; Sun et al., 2019; Alkhatib et al., 2020; Sun, 2020), etc. Based on the properties, ILs and DESs are screened with different criteria, for example, thermodynamic CO_2_ absorption capacity (CO_2_ solubility and Henry’s constant) (Liu et al., 2021) and (Henry’s constant, selectivity, relative polarity, and molar volumes) (Sumon and Henni, 2011), thermodynamic and kinetic performances for CO_2_ absorption (CO_2_ absorption capacity, viscosity, melting point, and Henry’s constant) (Farahipour et al., 2016; Zhang et al., 2021a) as well as CO_2_ chemical absorption properties (chemical equilibrium constants, Henry’s constants, and reaction enthalpies) (Moya et al., 2020), etc. Also, based on the thermodynamic analysis under specific operation conditions, screening has been conducted for both physical-based ILs and DESs (Zhang et al., 2016). In recent years, Zhou’s group has developed data-driven machine learning models to predict both the thermodynamic and kinetic properties of CO_2_ absorption with ILs/DESs (e.g., the CO_2_ solubility, absorbent viscosity and CO_2_ mass transfer property) (Song et al., 2020), providing a more comprehensive route for screening the promising ILs or DESs. However, the models are dependent on a large number of reliable experimental data, while the widely used COSMO-based models have a prior feature, resulting in the robust application value, especially when experimental data are missing. Moreover, COSMO-RS can combine with process simulation via Aspen to further evaluate the performance and conduct techno-economic analysis (García-Gutiérrez et al., 2016; De Riva et al., 2018). Therefore, COSMO-RS is a powerful tool for prior screening ILs/DESs.

In this work, the latest developed ILs and DESs were surveyed and collected, which were further combined with those have been collected in other review articles to provide a complete database. The IL/DES-based technologies were introduced, and the important properties were identified for each technology, providing criteria for IL/DES screening. Because not all the properties of ILs/DESs were provided, COSMO-RS was combined to predict the properties required in screening. Subsequently, the desirable ILs and DESs were screened, selected, and suggested for different IL/DES-based technologies.

## 2 Technologies for CO_2_ separation with ionic liquids/deep eutectic solvents

### 2.1 Ionic liquids/deep eutectic solvents as solvents

Over the past two decades, ILs/DESs as absorbents for CO_2_ capture have been widely studied (Smith et al., 2014; Zeng et al., 2017; Hansen et al., 2020). The extensive attention and research are based on their desirable properties, such as negligible vapor pressure, stable thermodynamic and chemical properties, tunable and designable structures, and good solubility for CO_2_. In particular, the negligible vapor pressure can simplify the CO_2_ absorption process.

#### 2.1.1 Ionic liquids as solvents

Blanchard et al. first reported that supercritical CO_2_ could be effectively dissolved in IL [C4MIM][PF6] at 25°C and 40 MPa, but ILs were insoluble in CO_2_ (Blanchard et al., 2001). Since then, research on the CO_2_ capture by ILs as absorbents has been reported extensively. According to the difference in capture mechanisms, ILs can be treated as two classes, i.e., the physical-based absorbents and chemical-based absorbents. The former represents the conventional ILs being able to interact with CO_2_ physically, while the latter represents the functionalized ILs being able to interact with CO_2_ chemically. In conventional ILs, anions usually play a leading role in the CO_2_ capture, while the capture effect is relatively less affected by cations (Anthony et al., 2005; Ramdin et al., 2012; Dai et al., 2017).

Similar to other physical absorbents, the CO_2_ absorption capacity of these physical-based ILs is inferior to those of traditional organic amines. In order to further enhance and improve the CO_2_ absorption capacity, researchers developed functionalized ILs with chemical interaction sites, such as carboxyl-based ILs, amino-based ILs, and amino acid-based ILs (Zeng et al., 2017). Bates et al. (2002) first prepared the amino-functionalized IL [NH_2_P-BIN][BF_4_] to capture CO_2_, with an absorption capacity up to 0.50 mol/mol. Xue et al. (2011) reported the CO_2_ solubility in amine-functionalized IL [AemMIM][Tau] up to 0.90 mol/mol. Later, the functionalized ILs with the anions introduced by amines were proposed. For example, Gurkan et al. (2010) presented two amino acid-functionalized ILs, [P_66614_][Pro] and [P_66614_][Met], both of which exhibited CO_2_ absorption capacity up to 0.90 mol/mol, near a 1:1 stoichiometry, and subsequently, an array of non-amino-functionalized ILs were developed (Lin et al., 2019b). To further improve the absorption capacity, Wang et al. prepared and synthesized the pyridine-based IL [P66614][3-OMe3-2-Op] (Luo et al., 2014), demonstrating the absorption capacity of up to 1.60 mol/mol under 20°C and 0.1 MPa because of the two interaction sites presented between oxygen and nitrogen in the anion and CO_2_.

Since the industry is more interested in the mass absorption capacity, researchers have made great efforts to improve and enhance the mass absorption capacity of ILs for CO_2_ capture. For example, Zhang et al. (2013) developed a novel kind of bisamino-functionalized IL, whose mass absorption capacity of CO_2_ can reach 18.5%. Chen et al. (2016) prepared a functionalized IL [P_4442_]_2_ [IDA] with the highest molar CO_2_ absorption capacity (1.69 mol/mol), corresponding to the mass absorption capacity of 2.84 mol/kg under 40°C and 0.1 MP. However, the viscosity of [P_4442_]_2_ [IDA] can be increased by almost 15 times after the absorption of CO_2_.

In a real process of CO_2_ separation, in addition to the CO_2_ absorption capacity (solubility), viscosity is also a key factor to consider. This is particularly essential for the IL-based technology, as the viscosity of most developed IL is much higher than the conventional solvents (Jiang et al., 2019a). Functionalized ILs have an even higher viscosity than conventional ones, and the absorption with CO_2_ is, in general, accompanied by a chemical reaction that further increases the viscosity (Liu et al., 2009; Sistla and Khanna, 2015; Ma et al., 2019). Therefore, it is necessary to develop ILs with low viscosity. Recently, Zhang et al. found an effective method to reduce the viscosity of ILs by introducing ether groups into cations, and increasing the number of ether-oxygen bonds decreased the viscosity (Zeng et al., 2015). Adding molecular solvent to an IL-based hybrid is another effective method to reduce viscosity. For example, when an organic amine solvent is added to ILs, the viscosity is significantly reduced, while the CO_2_ absorption capacity is not significantly weakened (Zhao et al., 2011).

Furthermore, in practical CO_2_ capture processes, the CO_2_ selectivity to other gases should be carefully considered (Ma et al., 2018). This is because ILs will serve as either a physical absorbent only, or the absorbent with both chemical and physical contributions (Ma et al., 2021) that is different from the conventional chemical absorbents. For instance, in the CO_2_ capture from natural gas or biogas, Zeng et al. (2015) designed and synthesized ether-based functionalized ILs, and their selectivity of CO_2_/CH_4_ was improved by 50% compared with non-functionalized ILs. Additionally, the interaction between IL and CO_2_, in general, is weak compared with the amine-based absorbents, and the focus has been specifically concerned on the desorption enthalpy in the research.

#### 2.1.2 Deep eutectic solvents as solvents

As a new type of “green solvents,” DESs can be regarded as IL analogous, and it has unique advantages such as lower preparation cost, biodegradability, and more eco-friendly properties when compared with ILs (Zhang et al., 2012; Smith et al., 2014). DESs have received wide attention for gas separation, especially CO_2_ capture (Smith et al., 2014; Hansen et al., 2020). When DESs are used as absorbents for CO_2_ capture, they can be divided into physical- and chemical-based absorbents, just like ILs. Most conventional DESs capture CO_2_ by means of physical absorption, while functionalized DESs like superbase-based DESs can capture CO_2_ by means of chemical absorption (Sarmad et al., 2017b). The study by Sarmad et al. (2017b) showed that DESs are promising alternatives to ILs because the solubility of CO_2_ in DESs is even higher than the similar IL counterparts. Recently, Huang et al. (Liu et al., 2019) found that the CO_2_ solubility in the DES (ChCl + urea) can be accommodated by the HBA/HBD molar ratios, following the order of 1:2 > 1:1.5 > 1:2.5, which leads to the fact that the selectivities of gas pairs for CO_2_/H_2_S and CO_2_/CH_4_ can be tuned by controlling the HBA/HBD molar ratios. Their study also explored the mechanism of CO_2_ capture at the molecular level, that is, it was dominated by the free volume inside DESs. However, the ChCl-based DESs have higher viscosities than the conventional ILs (Smith et al., 2014; Hansen et al., 2020).

In order to improve CO_2_ absorption capacity, superbase-based DESs with chemical absorption have been developed. Wang et al. (2010a) used DESs/ILs and superbase mixtures to prepare the superbase-based DES/ILs. This study clarified the role of the superbase in CO_2_ capture, that is, the superbase enables abstracting acidic hydrogens from the imidazolium ring of cation in ILs to enhance the affinity with CO_2_, increasing CO_2_ capture performance. Other publications have also proved that adding superbases or basic groups into the DES/IL systems can evidently improve the CO_2_ absorption capacity (Wang et al., 2010b; Wang et al., 2011). It should be noted that the addition of these superbases or basic groups always increases viscosity, which is unfavorable to the absorption and increases the energy usage for transporting absorbent in the CO_2_ separation process.

In short, both ILs and DESs show strong potential for industrial application in CO_2_ capture, thereby replacing traditional organic amine absorbents to overcome solvent volatility loss, equipment corrosion caused by organic amine absorbents as well as high energy usage for absorbent regeneration. Physical-based ILs show relatively low CO_2_ solubility and selectivity of CO_2_ to other gases while relatively low viscosity also. The task-specific functionalized ILs enhance CO_2_ solubility and selectivity, but generally possess high viscosity, complex preparation steps, high synthesis cost, and high absorption enthalpy. In comparison, DESs have received extensive research attention with the simpler preparation process, lower synthesis cost, and greener biodegradability, but generally have higher viscosities than ILs.

When ILs/DESs are used as a liquid absorbent, and the physical absorption-based technology is chosen, the CO_2_ mass absorption capacity is important, the viscosity needs a particular concern, and the selectivity is another important property when screening and suggesting ILs. Instead, for the chemical absorption-based technology, the CO_2_ mass absorption capacity, the viscosity, including that after CO_2_ absorption, and the desorption enthalpy are three primary properties, while the selectivity may need to concern a certain extent.

### 2.2 Nano-confined ionic liquids/deep eutectic solvents

#### 2.2.1 Nano-confined ionic liquids

In order to overcome the high viscosity for functionalized ILs and low selectivity for conventional ILs as liquid absorbents, the so-called nano-confined ILs, that is, ILs are confined in nanoporous matrices, such as metal-organic frameworks (MOFs), molecular sieves, silica, and porous carbons, with their spatial geometrical dimensions have been proposed. In this case, either or both the nanoporous matrix (rigid host) and IL (soft guest) can be suitably modified for task-specific separation processes. Nano-confined ILs can effectively reduce the amount of ILs and overcome their viscosity issues in the potential application, resulting in hybrid materials with distinct advantages like low energy usage for regeneration, high selectivity, and recoverable CO_2_ sorption. It was found that the conventional nano-confined ILs in silica could enhance CO_2_ sorption capacity and rate. Shi and Luebke (2013) studied CO_2_ sorption performance in [HMIM][Tf_2_N] confined into silica slit pores with widths from 2.5 to 4.5 nm using molecular simulations. It was found that the CO_2_ solubility in the confined IL is significantly higher than that in the relevant bulk IL due to the increased molar volume of confined ILs. In addition to the conventional ILs, confining functionalized ILs have also been reported for CO_2_ capture. Zhang et al. (2006) first supported the tetrabutylphosphonium amino acid ILs on silica gels to achieve fast and reversible CO_2_ sorption with the capacity of 0.50 mol/mol. It is worth noting that, when most of the pore surface of support is occupied by the immobilized IL, the CO_2_ sorption capacity of nano-confined ILs is dominated by the immobilized ILs rather than solid adsorbent (Ren et al., 2012). Similar to the bulk ILs, the CO_2_ sorption capacity is always dependent on the IL molecular structures. For instance, in terms of a list of amino acid-based ILs confined in porous silica, the CO_2_ sorption performance was mainly dominated by anions, and the highest CO_2_ sorption capacity was presented at the IL with the maximum number of amino groups. Also, the nano-confined IL with the longer cationic alkyl side chain corresponds to the lower CO_2_ sorption capacity (Ruckart et al., 2015). These studies and findings indicate that it is essential to put the focus on ILs, and CO_2_ absorption capacity of bulk ILs can be used as an index to screen and suggest ILs for confinement.

In some cases, the performance of confined ILs depends on both ILs and supports. Research has demonstrated that MOF is a promising class of materials for confining ILs (Zhang et al., 2017). Ban et al. (2015) confined [BMIM][Tf_2_N] into the nanocages of ZIF-8 to achieve a highly selective gas separation for CO_2_/N_2_ and CO_2_/CH_4_ by adjusting the molecular sieving properties of ZIF-8. The molecular simulation by Vicent-Luna et al. (2013) indicated that adding ILs into the pore of MOFs can intensify the CO_2_ sorption capacity at low pressures. Whereas the sorption behavior of N_2_ and CH_4_ cannot be affected. These findings may be ascribed to the “like dissolves like” principle. The IL@MOF composites can significantly increase the selectivity of CO_2_/N_2_, and a higher IL concentration in MOFs corresponds to a higher selectivity (Xue et al., 2016). Furthermore, the selectivity of CO_2_ separation can be improved by achieving a better dispersion behavior of ILs in MOFs, which explains MOF is a better candidate than the covalent organic framework (COF) (Xue et al., 2016). Han et al. (2021) synthesized a novel IL-ZIF-IL composite with the shell-interlayer-core structure using [TETA]L and ZIF-8, and investigated its CO_2_ capture performance. The IL-ZIF-IL composite exhibited a strong molecular sieving ability, and its performance can be adjusted by controlling the IL loading, thereby changing the thickness of the outer IL layer in the IL-ZIF-IL composite. Under the different operating conditions, the selectivity of CO_2_/CH_4_ (50:50) and CO_2_/N_2_ (15:85) mixtures was as high as 260–1990 and 1,688–5,572, respectively. This fully demonstrates the potential of nano-confined ILs in ZIF-8 for the selective separation of CO_2_. It also indicates that the selectivity in the IL-confined technology can be tuned by adjusting other parameters than IL itself.

Zeolites or molecular sieves are also a class of promising porous materials to confine ILs for CO_2_ capture (Yu et al., 2014; Ahmad et al., 2018). For example, Yu et al. (2014) employed conventional ILs ([C_n_MIM][Br], *n* = 4, 6, 8, and 10) as ship in a bottle synthesized in NaY zeolite to prepare [C_n_MIM][Br]@NaY composites successfully. In this study, [C_n_MIM][Br] was incorporated inside NaY, leading to higher stability of the confined ILs than the bulk counterparts. Among the studied ILs, [C_4_MIM][Br] has the highest CO_2_ sorption capacity of up to 0.456 mol/kg, which proved the potential of confined IL in zeolites for CO_2_ capture. Ahmad et al. (2018) prepared poly [VEMIM][Tf_2_N]/zeolite composite and found that poly [VEMIM][Tf_2_N]/zeolite composite can improve CO_2_ sorption capacity when compared with the single poly [VEMIM][Tf_2_N].

In addition, combining ILs and membrane materials to prepare IL-based membranes is another alternative strategy to avoid the high viscosity of ILs, and more importantly, the gas separation performance can also be efficiently improved. Here, most of the work is based on polyIL as the supported polymer membrane. Compared to a pure polyIL membrane, the polyIL-IL composite membrane containing free IL can increase the permeability of CO_2_, N_2_, and CH_4_ by 300%–600% (Bara et al., 2008). For mixed matrix membranes (MMMs) containing polyILs or polymers, free ILs and porous particles, the presence of ILs can improve the interfacial adhesion between particles and organic polymers, increasing CO_2_ permeability and selectivity (Hudiono et al., 2010; Hudiono et al., 2011). Hao et al. (2013) investigated poly (RTIL)/RTIL/ZIF-8 MMMs for natural gas sweetening and post-combustion CO_2_ capture. In their study, the poly (RTIL) was [VBIM][Tf_2_N], and the RTIL was [EMIM][BF_4_], [EMIM][Tf_2_N], and [EMIM][B(CN)_4_]. It was found that the [VBIM][Tf_2_N]/[EMIM][B(CN)_4_]/ZIF-8 system showed the impressive CO_2_/N_2_ separation performance with the selectivity of up to 21 at 35°C and 3.5 bar.

#### 2.2.2 Nano-confined deep eutectic solvents

DESs, as a novel class of IL analogous, have also been confined into solid materials to capture CO_2_. Lin et al. (2021) synthesized two-dimension materials of nano-confined DESs using DES (ChCl + ethylene glycol) and the nanoslits of titanium carbide (Ti_3_C_2_T_x_), and the properties of this material were determined by hydrogen bonds (HBs) between DESs and solid materials. The nano-confined DES demonstrated good thermal stability, long-term durability, and relatively high selectivity of CO_2_ separation, in which the selectivity of CO_2_/N_2_, CO_2_/CH_4_, and CO_2_/H_2_ are as high as 319.15, 249.01, and 12.38, respectively. Amira et al. (2018) first reported the DES-based membranes by incorporating DES + ethylene glycol (1:3) into polyvinylidene fluoride-co-polytetrafluoroethylene to capture CO_2_. It was found that the CO_2_ permeation was up to 25.5 × 103 GPU (gas permeation unit), but the selectivity of CO_2_/N_2_ was only 2.0. (Lin et al., 2019a) further developed the DES-based membranes and prepared the graphene oxide (GO) supported DES membranes by confining ChCl + ethylene glycol with different HBA/HBD molar ratios into the GO nanoslits. The composite membranes demonstrated remarkable permeation selectivity of CO_2_/N_2_ (>400), CO_2_/CH_4_ (>300), and CO_2_/H_2_ (>20), superior thermodynamic stability, and long-term endurance. The intensifying mechanism of CO_2_ capture was also investigated by using molecular dynamics simulations, that is, GO provided nanoconfined space for the DES, resulting in the unique spatial configuration, as well the interaction of DES-GO weakened the original interaction between DES molecules, which enlarges the free volume and prompts the gas diffusion.

It is evident that the nano-confined DESs/ILs can overcome the major shortages of bulk ILs/DESs e.g., fluidity, high viscosity, and slow diffusion. However, it should be noted that the nano-confined DESs/ILs as a class of hybrid material consisted of non-freely moving liquids and porous solids, and the loss of liquid phase cannot be negligible. Moreover, nano-confined DESs/ILs as macroscopic solid materials may change the CO_2_ capture process from absorption to adsorption (or membrane separation), which is suitable for the CO_2_ streams with low CO_2_ concentration and low gas flow rate, instated of those with high-concentration CO_2_ capture at high gas flow rates. Therefore, IL/DES-based liquid absorbents still show their advantages, and different IL/DES-based technologies need to be developed for different cases.

Meanwhile, combining the features of ILs/DESs and IL/DES-based technologies, the key properties can be quite different. When ILs/DESs are used as a solvent, if it is a physical-based technology, CO_2_ solubility in weight (mass)-basis, selectivity, and viscosity need to be considered simultaneously, while for the chemical-based one, CO_2_ solubility in weight-basis, viscosity before and after CO_2_ absorption as well as desorption enthalpy should be concerned. For the advanced nano-confined ILs/DESs, CO_2_ solubility in weight-basis is the primary one, and desorption enthalpy may need to be included, while concerns on viscosity and selectivity can be mitigated.

## 3 Literature survey, data collection, properties perdition, and ionic liquids/deep eutectic solvents screening

Considering the importance of CO_2_ capture and separation, a lot of ILs and DESs have been reported and summarized in different review articles, while more are continuously reported individually. To collect ILs and DESs that have been developed together with their properties, firstly, the published review articles were summarized to accumulate the ILs and DESs that were summarized, and then the recently developed or reported ILs and DESs were surveyed. All the reviewed and surveyed ILs and DESs were collected together with the properties, including CO_2_ solubility, viscosity, selectivity/heat of absorption, and for those without providing such properties, COSMO-RS was used to predict for the promising ILs/DESs. Finally, top 10 ILs and DESs were suggested for different IL/DES-based technology developments.

During this procedure, the concern on selectivity depends on the gas streams for CO_2_ separation. Since selectivity is more essential for the pure physical-based ILs and DESs, which are preferable for the gas streams with relatively high CO_2_ content, such as biogas or natural gas (CH_4_+CO_2_), synthesis gas (H_2_+CO_2_, H_2_+CO_2_+CO), here CH_4_, CO, and H_2_, together with N_2_, were also concerned in order to estimate the ideal selectivity.

It should be pointed out that corrosion is an important property, while to the best of our knowledge, the relevant study on ILs/DESs is still limited, and thus such property was excluded in screening ILs/DESs.

### 3.1 Summary of previous review articles

Rashid (Rashid, 2021) reviewed the ILs developed in 2010–2020 to separate gases such as CO_2_, etc., where imidazolium-, amine-, phosphonium- and guanidium-based ILs were reviewed for CO_2_ separation, and [BMIM]^+^ was identified as the most-widely used cation. Shama et al. (2021) summarized the synthesis protocol and process, carbon capture capacity, absorption type (physical or chemical), as well as merits and demerits of ILs developed in 2006–2020. [Gly][ChLac], [Gly][EMIMCl], [BMIM][PF_6_], [EMIM][Tf_2_N], [HMIM][Tf_2_N], [ChCl][Urea], [ChCl][Gly], and [ChCl][EG] were included as physical absorption, and phosphonium-, amine-, imidazolium-, and choline-based ILs were collected as the chemical absorption. Sood et al. (2021) evaluated the recent advances in the IL-based CO_2_ capture in 2017–2020/2021, in which imidazolium-, phosphonium-, and amine-based ILs were reviewed, and [EMIM][NTF_2_] was identified as a profitable IL. Pellegrini et al. (2021) introduced ILs as the water-lean solvent for CO_2_ capture, and amine-, imidazolium-, and ammonium-based ILs were specifically discussed. Also, DESs, including ChCl:urea and polyamine hydrochloride:thymol, were introduced for CO_2_ capture.

Liu et al. (2021) summarized CO_2_ solubility in physical- and chemical-based ILs and DESs. In terms of physical-based ILs, ammonium-, phosphonium-, imidazolium-, pyrrolidinium-, and sulfonyl-based were reviewed, and [BMIM][BF_4_] (4.20 mol/kg, 273.15 K, 20.89 bar), [AMIM][Tf_2_N] (3.88 mol/kg, 313.2 K, 57.1 bar), and DEA][Bu] (3.71 mol/kg, 303 K, 99.35 bar) were selected as top three. For those chemical-based, imidazolium-, imide-, gunidinium-, phosphonium-, amino functionalized- and ammonium-based were collected, and [DETAH][Im] (11.91 mol/kg), [DETAH][Py] (11.39 mol/kg), and [DETAH][Gly] (10.15 mol/kg) were selected as the top three. The physical-based DESs were categorized into phosphonium- and ammonium-based as well as [ChCl][EA], [ChCl][U], [ChCl][1,2-propanediol], [L-Arg][Gly], [GuaCl][EA], [BHDECl][LA], and [BHDECl][acetic acid]. [N_4444_Cl][DECA] (1:2) showed the highest solubility of 1.52 mol/kg at 298.15 K and 19.9 bar. The chemical-based DESs were classified into amine-, imidazolium-, ammonium-based as well as DESs comprised of DBU and TBD combined with various alcohols including ethylene glycol, benzyl alcohol, 2-imidazolidone, dimethylolurea, 1,3-dimethylurea, and MDEA. [TBD][EG] (1:4) delineated the highest solubility of 12.9 mol/kg at 298.15 K and 1 bar.

Wibowo et al. (2021) summarized the application of DESs for CO_2_ capture from gas streams, specially syngas, where CO_2_ solubilities in the DESs developed in 2012–2020 were summarized. This review covered the DESs composed of ChCl as HBA mixed with various HBDs, including triethylene glycol, ethylene glycol, urea, glycerol, guiacol, and MEA. Besides, imidazolium-, phosphonium- and ammonium-based DES as well as [L-Arg][Gly] were included. Hansen et al. (2020) reviewed the fundamentals and applications of DES. Here, ammonium-, phosphonium-, and imidazolium-based DESs as well as the DESs formed by the combination of ChCl with urea, ethylene glycol, levulinic acid, alcium chloride hexahydrate, and furfuryl alcohol were collected.Wang et al. (2021b) reviewed functionalized and non-functionalized DES used for CO_2_, SO_2_, and NO capture. Choline-, amine-, and ammonium-based DESs in addition to [Gua][MEA], [DBN][DMLU], [DBN][DMU], [DBN][EU], [UEC][EDA], and [DBN][EU] were reported. In terms of physical-based DESs, [TPAC][MEA] exhibited the highest capacity of 1.71–3.53 mol/kg, and [MEAC][EDA] showed the highest capacity of 8.86 mol/kg within the functionalized DESs.

### 3.2 Review of ionic liquids and deep eutectic solvents developed since 2021

According to the survey, the developed ILs and DESs up to 2020 have been well collected and summarized, and thus, the newly published ILs and DESs since 2021 were surveyed and collected in this part based on the Web of Science (WOS) database.

#### 3.2.1 Physical-based ionic liquids

11 studies have been focused on physical-based ILs since 2021. The studied ILs and corresponding properties and other information are summarized in Table 1. Here, Thapaliya et al. (2021) synthesized two tetracationic ILs (DABCO-T and DABCO-B) and four dicationic ILs (2OEt-Im, 3OEt-Im, C_8_-Im, and C_10_-Im), among which 3OEt-Im represented the highest capacity of CO_2_ (0.433 mol/kg at 298.15 K, 10 bar). Jalili et al. (2022) synthesized 1-benzyl-3-methylimidazolium bis (trifluoromethylsulfonyl) imide ([BZMIM][Tf_2_N]) IL. The CO_2_ solubility reached 1.919 ± 0.054 mol/kg at 303.15 K and 30.5 bar, and the selectivity over H_2_ reached 2.89.

**TABLE 1:** Physical-based ILs reported in 2021–2022.

Abbreviation	Full name	*T* (K)	*P* (bar)	Viscosity (cP)		Selectivity	Absorption capacity (mol/kg)	References
				Initial	Final			
[HMIM][TCB]	1-hexyl-3-methylimidazolium tetracyanoborate	293	28	—	—	—	0.992	Amiri et al. (2021)
[BMIM][Br]	1-butyl-3-methylimidazolium bromide	303.15	1	—	—	2.417 (H_2_S/CO_2_)	0.114	Adhi et al. (2021)
P [VBIm][SCN]	Poly (1-butyl-3-vinylimidazolium thiocyanate)	298.15	1	—	—	—	0.525	Noorani and Mehrdad, (2021a)
P [VBIm][BF4]	Poly (1-butyl-3-vinylimidazolium tetrafuoroborate)			—	—	—	0.216	
P [VBIm][Br]	Poly (1-butyl-3-vinylimidazolium bromide)			—	—	—	0.136	
[EMIM][FAP]	1-ethyl-3-methylimidazo tris (pentafluoroethyl) trifluorophosphate	293.15	50	75 at 293	—	—	∼4.195	Wu et al. (2021)
[BMIM][FAP]	1-butyl-3-methylimidazo tris (pentafluoroethyl) trifluorophosphate			93 at 293	—	—	∼4.628	
[HMIM][FAP]	1-hexyl-3-methylimidazo tris (pentafluoroethyl) trifluorophosphate			116 at 293	—	—	∼5.791	
[(2,2)O_Et_Im][Tf_2_N]_2_	1,1′,3,3′-bis(3,6-dioxaoctane-1,8-diyl) bis(imidazolium) bis (trifluoromethanesulfonyl) imide	298.15	10	—	—	—	0.368	Thapaliya et al. (2021)
[(3,3)O_Et_Im][Tf_2_N]_2_	1,1′,3,3′-bis(3,6,9-trioxaundecane-1,11-diyl)bis(imidazolium) bis (trifluoromethanesulfonyl) imide			—	—	—	0.433	
[(3,3)O_Et_DABCO][Tf_2_N]_4_	1,1′,4,4′-bis(3,6,9-trioxaundecane-1,11-diyl)bis (1,4-diazoniabicyclo [2.2.2]octane) bis(trifluoromethanesulfonyl) imide			—	—	—	0.261	
[(3,3)O_Et_DABCO][BETI]_4_	1,1′,4,4′-bis(3,6,9-trioxaundecane-1,11-diyl)bis(1,4-diazoniabicyclo [2.2.2]octane) bis(pentafluoroethanesulfonyl) imide			—	—	—	0.193	
[bis(octamethylene)-bis(imidazolium)][Tf_2_N]_2_	1,1′,3,3′-bis(octamethylene)bis (imidazolium) bis(trifluoromethanesulfonyl) imide			—	—	—	0.382	
[bis(decamethylene)-bis(imidazolium)][Tf_2_N]_2_	1,1′,3,3′-bis(decamethylene)bis (imidazolium) bis(trifluoromethanesulfonyl) imide			—	—	—	0.302	
[N_2222_][PF_6_]	Tetraethylammonium hexafluorophosphate	303.15	40	—	—	1.57 (CO_2_/CH_4_)	1.486	Wang et al. (2020)
[N_4444_][PF_6_]	Tetrabutylammonium hexafluorophosphate			—	—	1.24 (CO_2_/CH_4_)	1.277	
[C_12_MIM][PF_6_]	1-dodecyl-3-methylimidazolium hexafluorophosphate			—	—	5.53 (CO_2_/CH_4_)	3.643	
[C_16_MIM][PF_6_]	1-hexadecyl-3-methylimidazolium hexafluorophosphate			—	—	4.21 (CO_2_/CH_4_)	2.08	
[BZMIM][Tf_2_N]	1-benzyl-3-methylimidazolium bis(trifluoromethylsulfonyl) imide	303.15	30.496	—	—	2.89 (CO_2_/H_2_S)	4.23	Jalili et al. (2022)
[EMIM][Tf_2_N]	1-ethyl-3-methylimidazolium bis(trifluoromethylsulfonyl)imide	298.15	40	—	—	—	∼2.746	Peng et al. (2021)
[BMIM][Tf_2_N]	1-butyl-3-methylimidazolium bis(trifluoromethylsulfonyl) imide		35	—	—	—	∼2.201	
[HMIM][Tf_2_N]	1-hexyl-3-methylimidazolium bis(trifluoromethylsulfonyl) amide		40	—	—	—	∼2.421	
[OMIM][Tf_2_N]	1-octyl-3-methylimidazolium bis(trifluoro-methylsulfonyl) imide		40	—	—	—	2.499	
[EMIM][CH_3_SO_3_]	1-ethyl-3-methylimidazolium methanesulfonate	293.15	45	—	—	—	3.2368	Safarov et al. (2021)
[APMIm][Tf_2_N]	1-aminopropyl-3-imidazolium bis (trifluoromethylsulfonyl) imine	303.15	1	—	—	—	0.706	Pan et al. (2021)
[BVIM][Tf_2_N]	1- butyl-3-vinylimidazolium bis(trifluoromethylsulfonyl)imide	303.2	71.1	77 at 298.15 K	—	—	6.268	Yim et al. (2021)
[N_4222_][Tf_2_N]	Butyltriethylammonium bis(trifluoromethylsulfonyl) imide	303.2	124.2	99 at 298.15 K	—	—	4.180	
[P_4441_][Tf_2_N]	Tributylmethylphosphonium bis(trifluoromethylsulfonyl) imide	303.2	103.9	209 at 298.15 K	—	—	6.063	

Besides, the research work was also conducted for the reported ILs. For example, Wu et al. (2021) studied the CO_2_ solubility of three ILs ([EMIM][FAP], [HMIM][FAP], and [BMIM][FAP]) in a wide temperature (293.15–333.15 K) and pressure (0–50 bar) range. Safarov et al. (2021) chose 1-ethyl-3-methylimidazolium methanesulfonate [EMIM][CH_3_SO_3_] with a solubility of 3.3 mol/kg at 293.15 K and 45.77 bar. Peng et al. (2021). investigated [HMIM][Tf_2_N], [BMIM][Tf_2_N], [EMIM][Tf_2_N], and [OMIM][Tf_2_N] at 298.15–328.15 K and 20–50 bar. Yim et al. (2021) selected [BVIM][Tf_2_N], [N_4222_][Tf_2_N], and [P_4441_][Tf_2_N].

#### 3.2.2 Chemical-based ionic liquids

16 studies have been focused on chemical-based ILs since 2021, which are listed in Table 2. During this period, Zema et al. (2021) prepared K^+^ chelated dual functional ILs using monoethanolamine (MEA), 2-amino-1-butanol (AMB), 2-amino-2-methyl-1-propanol (AMP), and DL-1- amino-2-propanol (DLAMP), and it was found that CO_2_ solubility (mol/kg) followed [K (AMP)_2_][Im] > [K (DLAMP)_2_][Im] > [K (AMB)_2_][Im] > [K (MEA)_2_][Im]. Qu et al. (2021) synthesized amino acid ILs (AAILs), including 1-methoxylbutyl-3-methylimidazolium lysine [MOBMIM][Lys], 1-methoxylbutyl-3-methylimidazolium arginine [MOBMIM][Arg], 1-methoxylbutyl-3-methylimidazolium glycine [MOBMIM][Gly], and 1-methoxylbutyl-3-methylimidazolium histidine [MOBMIM][His], and [MOBMIM][Gly] exhibited the highest mass absorption capacity (9.66 mol/kg) and the lowest viscosity. After the absorption, [MOBMIM][His] and [MOBMIM][Gly] stayed in the liquid phase, while [MOBMIM][Arg] and [MOBMIM][Gly] turned to gel. Recently, Zailani et al. (2022) synthesized ammonium based protic ILs using 2-ethylhexylammonium and bis-(2-ethylhexyl) ammonium coupled with pentanoate, hexanoate, and heptanoate ([EHA][C_5_], [EHA][C_6_], [EHA][C_7_], [BEHA][C_5_], [BEHA][C_6_], and [BEHA][C_7_]). The absorption capacity (mol/kg) decreased in the order of [C_5_] < [C_6_] < [C_7_], and [EHA][C_7_] showed the highest absorption capacity (∼3.0 mol/kg at 29 bar and room temperature). Taking the advantage of metal ion coordination, Suo et al. (2022) synthesized metal-ion-amino-based IL using metal salts {[M(OctNH_2_)_4_][NTf_2_]_2_, M = Co, Cu, Ni, Zn, and Mg}. The CO_2_ absorption capacity (mol/kg) followed the order of [Li(OctNH_2_)_4_][NTf_2_]_2_ > [Mg (DecNH_2_)_4_][NTf_2_]_2_ ≈ [Mg(OctNH_2_)_4_][NTf_2_]_2_ > [Ni(OctNH_2_)_4_][NTf_2_]_2_ > [Ni(DecNH_2_)_4_][NTf_2_]_2_ > [Co(OctNH_2_)_4_][NTf_2_]_2_ > [Zn(OctNH_2_)_4_][NTf_2_]_2_ > [CuMg(DecNH_2_)_4_][NTf_2_]_2_ > [Cu(OctNH_2_)_4_][NTf_2_]_2_ > [CuZnNiMg(DecNH_2_)_4_][NTf_2_]_2_ > [CuZn(DecNH_2_)_4_][NTf_2_]_2_. Additionally, it was stated that the addition of metal sites led to a significant enhancement of thermal stability. Keller et al. (2022) designed and synthesized aprotic N-heterocyclic anion ILs using triethyl (octyl)-phosphonium cations and different aprotic N-heterocyclic anions. [P_2228_][5MeBnIm], [P_2228_][3SH-4Triz], and [P_2228_][BnTriz] [P_2228_][5BrInda] pose high melting point before and after CO_2_ absorption (>333 K), while [P_2228_][4BrIm], [P_2228_][4Br3MePyra], [P_2228_][3ClInda], [P_2228_][4BrInda], [P_2228_][6BrInda], [P_2228_][6CNInda], and [P_2228_][3IPyra] were studied for CO_2_ absorption. It was observed that the indazolide-/pyrazolide-based ILs became solid after absorption, while [P_2228_][4BrIm] remained liquid. Among them, [P_2228_][4Br3MePyra] exhibited the highest solubility of 2.498 mol/kg at 321.8 K and 0.798 bar, and the uptake capacity (mol/kg) decreased in the order of [4Br3MePyra]^−^ > [6BrInda]^−^ > [3ClInda]^−^ > [4BrInda]^−^ > [3IPyra]^−^ > [4BrIm]^−^ > [6CNInda]^−^. Min et al. (2021) synthesized 1-ethyl-3- methylimidazolium glycinate ([EMIM][Gly]), and the presence of water enhanced the CO_2_ absorption capacity (8.645 mol/kg) at 25°C and 40 ml/min gas flow rate. Besides, Mei et al. (2022) restudied 1-ethyl-3-methylimidazolium acetate ([EMIM][OAc).

**TABLE 2:** Chemical-based ILs reported in 2021–2022.

Abbreviation	Full name		*T* (K)	*P* (bar)	Viscosity (cP)		Selectivity	Absorption capacity (mol/kg)	References
					Initial	Final			
[EHA][C_5_]	2-ethylhexylammonium pentanoate		298.15	30	680.4233 at 293.15	—	—	∼2.68	Zailani et al. (2022)
[EHA][C_6_]	2-ethylhexylammonium hexanoate				423.353 at 293.15	—	—	∼2.852	
[EHA][C_7_]	2-ethylhexylammonium heptanoate				288.4767 at 293.15	—	—	∼3.007	
[BEHA][C_5_]	Bis-(2-ethylhexyl) ammonium pentanoate				36.8410 at 293.15	—	—	∼1.834	
[BEHA][C_6_]	Bis-(2-ethylhexyl) ammonium hexanoate				33.7660 at 293.15	—	—	∼1.901	
[BEHA][C_7_]	Bis-(2-ethylhexyl) ammonium heptanoate				37.6123 at 293.15	—	—	∼2.126	
[EMIM][Gly]	1-ethyl-3- methylimidazolium glycinate		298.15	—	—	—	—	8.645	Min et al. (2021)
[DMEDAH] [Py]	N,N-dimethylethylenediamine pyrazole		295.15	1.01	—	—	—	5.25	Wang et al. (2021a)
[DMEDAH] [Im]	N,N-dimethylethylenediamine imidazole				—	—	—	4.91	
[DMEDAH] [Tz]	N,N-dimethylethylenediamine 1,2,4-triazole				—	—	—	4.32	
[K (AMP)_2_][Im]	K+ chelated 2-amino-2-methyl-1-propanol imidazole		333.2	1	138.7 at 332.2 K	—	—	4.183	Zema et al. (2021)
[K (AMB)_2_][Im]	K+ chelated 2-amino-1-butanol imidazole				53.34 at 332.2 K	—	—	3.832	
[K (DLAMP)_2_][Im]	K+ chelated dl-1-amino-2-propano imidazole				50.90 at 332.2 K	—	—	3.9	
[K (MEA)_2_][Im]	K+ chelated monoethanol amine imidazole				43.03 at 332.2 K	—	—	3.328	
[P_66614_][Benzim]	Trihexyltetradecyl- phosphonium benzimidazolide		295.15	1	1,087 at 298.15	—	—	1.21	Greer et al. (2021)
[P_2224_][4-Triaz]	Triethyl-butyl-phosphonium, 1,2,4, triazolide		353.15	3.080	—	—	—	1.85	Gohndrone et al. (2021)
[P_66614_][2-CNpyr]	Trihexyl-tetradecyl-phosphonium 2-cyanopyrrolide		313.15	0.937	—	—	—	1.207	
[P_66614_][SCH_3_BnIm]	Trihexyl-tetradecyl-phosphonium 2-methylthio-benzimidazolide		333.15	3.164	—	—	—	0.972	
[P_66614_][4-Triaz]	Trihexyl-tetradecyl-phosphonium, 1,2,4, triazolide		313.15	0.753	—	—	—	1.05	
[P_66614_][Inda]	Trihexyl-tetradecyl-phosphonium indazolide		313.15	3.216	—	—	—	1.561	
[P_66614_][BrBnIm]	Trihexyl-tetradecyl-phosphonium 6-bromo-benzimidazolide		313.15	2.693	—	—	—	1.41	
[P_66614_][CF_3_pyra]	Trihexyl-tetradecyl-phosphonium 3-trifluoromethyl-pyrazolide		313.15	4.092	—	—	—	1.661	
[P_66614_][BnIm]	Trihexyl-tetradecyl-phosphonium benzimidazolide		313.15	3.098	—	—	—	1.597	
[MOBMIM][Lys]	1-methoxylbutyl-3-methylimidazolium lysine		323.15	5	3,201.13	—	—	6.721	Qu et al. (2021)
[MOBMIM][His]	1-methoxylbutyl-3-methylimidazolium histidine				2,986.94	—	—	7.026	
[MOBMIM][Arg]	1-methoxylbutyl-3-methylimidazolium arginine				5,631.12	—	—	6.086	
[MOBMIM][Gly]	1-methoxylbutyl-3-methylimidazolium glycine				536.31	—	—	9.66	
[DBNH][Im]	1,5-diazabicyclo [4,3,0]non-5-ene/imidazole		313.2	1	8.3	—	—	3.38	Xiong et al. (2021)
[DBUH][Im]	1,8-diazabicyclo [5,4,0]undec-7-ene/imidazole				18.3	—	—	3.086	
[DBNH][Pyr]	1,5-diazabicyclo [4,3,0]non-5-ene/Pyrazole				5.1	—	—	3.381	
[DBUH][Pyr]	1,8-diazabicyclo [5,4,0]undec-7-ene/Pyrazole				9.7	—	—	3.041	
[EMIM][OAc]	1-ethyl-3-methylimidazolium acetate		298.15	1.836	—	—	—	2.16	Mei et al. (2022)
[Cu(OctNH_2_)_4_][Tf_2_N]_2_	—		298	1	—	—	—	0.061	Suo et al. (2022)
[Zn(OctNH_2_)_4_][Tf_2_N]_2_	—				—	—	—	0.192	
[Co(OctNH_2_)_4_][Tf_2_N]_2_	—				—	—	—	0.307	
[Ni(OctNH_2_)_4_][Tf_2_N]_2_	—				—	—	—	0.395	
[Mg(OctNH_2_)_4_][Tf_2_N]_2_	—				—	—	—	0.455	
[Li(OctNH_2_)_4_][Tf_2_N]_2_	—				—	—	—	0.654	
[Ni(DecNH_2_)_4_][Tf_2_N]_2_	—			—	—	—	—	0.377	
[Mg (DecNH_2_)_4_][Tf_2_N]_2_	—				—	—	—	0.455	
[CuZn(DecNH_2_)_4_][Tf_2_N]_2_	—				—	—	—	0.0238	
[CuMg(DecNH_2_)_4_][Tf_2_N]_2_	—				—	—	—	0.068	
[CuZnNiMg(DecNH_2_)_4_][Tf_2_N]_2_	—				—	—	—	0.0384	
[B_4_MPyr][L-Arg]	1-butyl-4-methyl pyridinium	Arginate	298.15	2	—	—	—	1.45	Noorani et al. (2021)
[B_4_MPyr][L-Lys]	1-butyl-4-methyl pyridinium	Lysinate			—	—	—-	1.24	
[B_4_MPyr][L-His]		Hisdinate			—	—	—-	1.11	
[B_4_MPyr][L-Tyr]		Tyrosinate			—	—	—-	0.966	
[B_4_MPyr][Gly]		Glycinate			—	—	—-	1.46	
[B_4_MPyr][L-Ala]		Alaninate			—	—	—-	1.42	
[B_4_MPyr][L-Val]		Valinate			—	—	—-	1.08	
[B_4_MPyr][L-Pro]		Prolinate			—	—	—-	1.06	
[Cho][Gly]	Cholinium glycinate		298.15	4	—	—	—	6.20	Noorani and Mehrdad, (2021b)
[Cho][Ala]	Cholinium alaninate				—	—	—	4.222	
[Cho][Val]	Cholinium valinate				—	—	—	1.093	
[P_4444_][OAc]	Tetra butyl phosphonium acetate		343.2	14.01	—	—	—	∼1.91	Pena et al. (2021)
[P_2228_][3ClInda]	Triethyl (octyl) phosphonium 3-chloroindazole		332.1	0.763	852 at 298.2	—	—	2.188	Keller et al. (2022)
[P_2228_][4Br3MePyra]	Triethyl (octyl) phosphonium 4-bromo-3-methyl-1H-pyrazole		321.8	0.798	280 at 298.2	—	—	2.298	
[P_2228_][4BrInda]	Triethyl (octyl) phosphonium 4-bromoindazole		332	0.639	1,030 at 298.1	—	—	2.124	
[P_2228_][6BrInda]	Triethyl (octyl) phosphonium 6-bromoindazole		332	0.833	982 at 298.1	—	—	2.334	
[P_2228_][4BrIm]	Triethyl (octyl) phosphonium 4-bromoimidazole		332.1	0.772	588 at 298.2	—	—	1.665	
[P_2228_][6CNInda]	Triethyl (octyl) phosphonium 6-cyanoindazole		337.3	0.479	956 at 298.2	—	—	1.629	
[P_2228_][3IPyra]	Triethyl (octyl) phosphonium 3-iodopyrazole		332.2	0.651	224 at 298.2	—	—	1.951	
[tetraEG (MIM)_2_][Br]_2_	3,30 -(tetraethyleneglycol-1,11-diyl)bis(1-methyl-1h-imidazolium) bromide		343.139	4.9916	39,631 at 333.149 K	—	—	0.233	Avila et al. (2021)
[tetraEG (MIM)_2_] [OAc]_2_	3,30 -(tetraethyleneglycol-1,11-diyl) bis(1-methyl-1h-imidazolium) acetate		343.051	4.9829	2,251.9 at 323.151 K	—	—	1.735	
[C_4_MIM]_2_ [Mal]	1-butyl-3-methylimidazolium malonate		343.106	4.9971	75,670 at 313.15 K	—	—	1.83	
[C_4_MIM]_2_ [Glut]	3-butyl-1-methylimidazolium glutarate		343.106	4.9971	19,082 at 303.148 K	—	—	1.834	
[DBUH][MLU]	1,8-diazabicyclo [5.4.0] undec-7-ene methyl urea		313	1	∼520 at 293.15 K	—	—	1.75	Fu et al. (2021b)
[DBUH]_2_ [DMU]	1,8-diazabicyclo [5.4.0] undec-7-ene 1,3-dimethylurea		313	1	∼45 at 293.15	—	—	2.68	Fu et al. (2021b)

#### 3.2.3 Physical-based deep eutectic solvents

12 studies have been focused on physical-based DESs since 2021, and their specific names, together with other information, are summarized in Table 3. Among them, two are newly synthesized (Deng et al., 2021; Pishro et al., 2021), while others are the previously synthesized DESs with additional efforts on e.g., high-pressure CO_2_ solubility (Haghbakhsh et al., 2021), the effects of water content (Liu et al., 2022), pressure/temperature/stirring rate/water content (Aboshatta and Magueijo, 2021), pressure/temperature/water content (Yan et al., 2022), HBA:HBD molar ratios (Luo et al., 2021; Qin et al., 2021), HBA and HBD nature (Rabhi et al., 2020; Haider et al., 2021; Luo et al., 2021; Mihăilă et al., 2021) on the CO_2_ solubility.

**TABLE 3:** Physical-based DESs reported in 2021–2022.

Abbreviation	Structure			*T* (K)	*P* (bar)	Viscosity (cP)		Selectivity	Absorption capacity (mol/kg)	References
	HBA	Molar ratio (HBA:HBD)	HBD			Initial	Final			
[EAHC][TEPA]	Ethanol amine hydrochloride	1:9	Tetraethylenepenta amine	303.15	16.03	—	—	—	9.671	Pishro et al. (2021)
[ChCl][TEG]	Choline chloride	1:3	Triethyleneglycol	303.15	26.196	—	—	—	0.992	Haghbakhsh et al. (2021)
[GI][AT]	Guanidine isothiocyanate	1:4	Acetamide	303.15	5.768	16.53 ± 0.15 at 303.15 K	—	97 (NH_3_/CO_2_)	0.214 ± 0.004	Deng et al. (2021)
[3Im][PTSA]	Imidazole	3:1	P-toluenesulfonic acid	303.15	14.475	∼55 at 302.5 K	—	—	1.0059	Qin et al. (2021)
[3.5Im][PTSA]		3.5:1			14.982	∼44 at 302.5 K	—	—	1.0642	
[4Im][PTSA]		4:1			14.821	∼42 at 302.5	—	—	1.0959	
[MTPPhBr][4 EG]	Methyltriphenylphosphonium Bromide	1:4	Ethylene glycol	303.15	10.19	—	—	—	0.438	Haider et al. (2021)
[MTPPhBr][4DEG]		1:4	Diethylene glycol		9.21	—	—	—	0.390	
[MTPPhBr][3GLY]		1:3	Glycerol		10.39	—	—	—	0.373	
[TBABr][OA]	Tetrabutylammonium bromide	1:2	Octanoic acid	313.15	40	—	—	—	2.15	Rabhi et al. (2020)
[TBABr][DA]	Tetrabutylammonium bromide	1:2	Decanoic acid			—	—	—	2.53	
[DL-menthol][DA]	DL-menthol	2:1	Dodecanoic acid			—	—	—	2.06	
[TBAB][DEC]	Tetrabutylammonium bromide	1:2	Decanoic acid	303.15	11.17	—	—	—	0.679	Luo et al. (2021)
[TBAB][HEX]	Tetrabutylammonium bromide	1:4	Hexanoic acid	303.15	13.35	—	—	—	0.954	
[TEAC][HEX]	Tetraethylammonium chloride	1:4	Hexanoic acid	303.15	12.63	—	—	—	0.837	
[ChCl][MDEA]	Choline chloride	1:7	N-methyldiethanolamine	—	—	∼75 at 303.15 K	—	—	1.389	Liu et al. (2022)
[ChCl][LvAc]	Choline chloride	1:3	Levulinic-acid	298.15	6	—	—	—	∼1.5	Aboshatta and Magueijo, (2021)
[ChCl][EG]	Choline chloride	1:2	Ethylene glycol	298.15	60	36 at 298.15 K	—	—	1.363	Mihăilă et al. (2021)
[ChCl][U]	Choline chloride	1:2	Urea			735.5 at 298.15 K	—	—	0.455	
[ChCl][MEA]	Choline chloride	1:5	Monoethanolamine	313.15	12.5	20 at 313.15 K	—	20 (CO_2_/CH_4_) at 313.15 K and 12.5 bar	∼3.775	Yan et al. (2022)

For the new DESs, Pishro et al. (2021) synthesized ammonium-based ones using ethanolamine hydrochloride (EAHC) and tetraethylenepentamine (TEPA) with various molar ratios and the one with 1:9 (molar ratio) exhibited the highest solubility of 9.671 mol/kg at 303.15 K and 16.03 bar.

Overall, choline chloride is widely used as HBA, while tetrabutylammonium bromide, methyltriphenylphosphonium bromide, imidazole, guanidine isothiocyanate, and ethanol amine hydrochloride were also used. In terms of HBD, various fatty acids (hexanoic, decanoic, dodecanoic acids, Levulinic-acid), alcohols (glycerol, ethylene glycol, diethylene glycol, triethylene glycol), amines (tetraethylenepenta amine, n-methyldiethanolamine, monoethanolamine), amides (urea, acetamide), and p-toluenesulfonic acid have been utilized.

#### 3.2.4 Chemical-based deep eutectic solvents

Nine studies have been focused on chemical-based DESs since 2021, as summarized in Table 4. Among them, three DESs are newly synthesized (Fu et al., 2021a; Ahmad et al., 2021; Qian et al., 2022), while others are the previously synthesized ones for various purposes, including the discussion about the water content (Liu et al., 2022) and estimating the effects of HBA:HBD molar ration, type of anion, and the alkyl chain (Cichowska-Kopczyńska et al., 2021).

**TABLE 4:** Chemical-based DESs reported in 2021–2022.

Abbreviation	Structure			*T* (K)	*P* (bar)	Viscosity (cP)		Selectivity	Absorption capacity (mol/kg)	References
	HBA	Molar ratio (HBA:HBD)	HBD			Initial	Final			
[ChCl][MEA]	Choline chloride	1:5	Monoethanolamine	—	—	∼25 at 303.15 K	—	—	6.17	Liu et al. (2022)
[ChCl][DEA]		1:6	Diethanolamine	—	—	∼340 at 303.15 K	—	—	3.893	
[MEAHCl][MDEA]	Monoethanolamine hydrochloride	1:3	Methyldiethanolamine	298.15	1	∼270	1,500	—	2.631	Ahmad et al. (2021)
[DEAHCl][MDEA]	Diethanolamine hydrochloride		Methyldiethanolamine			∼270	∼1,460	—	∼2.459	
[MDEAHCl][MDEA]	N-methyl diethanolamine hydrochloride		Methyldiethanolamine			∼200	∼410	—	∼1.35	
[DBU][Py]	1,8-Diazabicyclo [5.4.0] undec-7-ene	1:1	Pyrrolidine	303	1		—	—	6.5	Fu et al. (2021a)
[DBU][Pyr]		2:1	2-Pyrrolidinone			19.1	—	—	8.193	
[DBU][Oxa]			Oxazolidinone				—	—	6.336	
[DBU][*E*th]			Ethyleneurea			50.1	—	—	7.504	
[TBAC][AP]	Tetrabutylammonium chloride	1:4	3-amino-1-propanol		1.6	77.36 at 298.15 K	—	—	∼4.582	Cichowska-Kopczyńska et al. (2021)
[TBAB][AP]	Tetrabutylammonium bromide				1	84.69 at 298.15 K	—	—	∼3.854	
[TEAC][AP]	Tetraethylammonium chloride				0.6	42.37 at 298.15 K	—	—	∼4.076	
[Gly][MEA]	Glycine	1:4	Monoethanolamine	298.15	1.01	—	—	—	∼5.227	Qian et al. (2022)
[Pro][MEA]	Proline		Monoethanolamine			—	—	—	∼4.590	
[Pro][MDEA]	Proline		Monoethanolamine			—	—	—	∼1.056	
[Gly]: [MEA]/EG	Glycine:monoethanolamine	1:4/30 wt%	Ethylene glycol	303.15	1.01	—	—	—	∼5	Qian et al. (2022)
[Pro]: [MEA]/EG	Proline: monoethanolamine	1:4/30 wt%	Ethylene glycol	303.15	1.01	—	—	—	∼4.09	Qian et al. (2022)
[Et_4_N][Thy]:EG	Tetraethylammonium thymol	1:2	Ethylene glycol	298.18	1	292	—	—	5.23	Wang et al. (2021c)
[Et_4_N][Car]:EG	Tetraethylammonium carvacrol	1:2	Ethylene glycol	298.15	1	279	—	—	5.06	Wang et al. (2021c)
[Et_4_N][Thy]:4CH_3_-Im	Tetraethylammonium thymol	1:2	4-methylimidazole	298.15	1	1,298	—	—	4.86	Wang et al. (2021c)
[Et_4_N][Car]:4CH_3_-Im	Tetraethylammonium carvacrol	1:2	4-methylimidazole	298.15	1	891	—	—	4.75	Wang et al. (2021c)
[DBUH][MLU]:EG	1,8-diazabicyclo [5.4.0]undec-7-ene methyl urea	1:1	Ethylene glycol	313.15	1	∼400	—	—	3.14	Fu et al. (2021b)
[DBUH][MLU]:EG	1,8-diazabicyclo [5.4.0]undec-7-ene methyl urea	1:2	Ethylene glycol	313.15	1	∼220 at 293.15 K	—	—	2.70	Fu et al. (2021b)
[DBUH][MLU]:EG	1,8-diazabicyclo [5.4.0]undec-7-ene methyl urea	1:4	Ethylene glycol	313.15	1	—	—	—	2	Fu et al. (2021b)
[DBUH]_2_ [DMU]:EG	1,8-diazabicyclo [5.4.0]undec-7-ene 1,3-dimethylurea	1:1	Ethylene glycol	313.15	1	—	—	—	2.95	Fu et al. (2021b)
[DBUH]_2_ [DMU]:EG	1,8-diazabicyclo [5.4.0]undec-7-ene 1,3-dimethylurea	1:2	Ethylene glycol	313.15	1	∼190 at 293.15 K	-	—	3.70	Fu et al. (2021b)
[DBUH]_2_ [DMU]:EG	1,8-diazabicyclo [5.4.0]undec-7-ene 1,3-dimethylurea	1:4	Ethylene glycol	313.15	1	∼110 at 293.15 K	—	—	3.05	Fu et al. (2021b)
[EMIM][2CNpyr]:EG	1-ethyl-3-methylimidazolium 2- cyanopyrrolide	1:1	Ethylene glycol	298.15	1	—	—	—	∼3.5	Lee et al. (2021)
[EMIM][2CNpyr]:EG	1-ethyl-3-methylimidazolium 2- cyanopyrrolide	1:2	Ethylene glycol	298.15	1	45.1 ± 0.2 at 298.15 K	88.2 ± 1.5 at 298.15 K	—	2.59	Lee et al. (2021)
[DBUH][Car]:EG	1,8-diazabicyclo- [5,4,0]undec-7-ene carvacrol	1:4	Ethylene glycol	298.15	1	166 at 298.15 K	—	—	8.99	Wang et al. (2022b)
[DBUH][Car]:EG	1,8-diazabicyclo- [5,4,0]undec-7-ene carvacrol	1:3	Ethylene glycol	298.15	1	228 at 298.15 K	—	—	8.10	Wang et al. (2022b)
[DBUH][Car]:EG	1,8-diazabicyclo- [5,4,0]undec-7-ene carvacrol	1:2	Ethylene glycol	298.15	1	389 at 298.15 K	—	—	6.82	Wang et al. (2022b)
[DBUH][Thy]:EG	1,8-diazabicyclo- [5,4,0]undec-7-ene thymol	1:4	Ethylene glycol	298.15	1	179 at 298.15 K	—	—	9.08	Wang et al. (2022b)
[DBUH][Thy]:EG	1,8-diazabicyclo- [5,4,0]undec-7-ene thymol	1:3	Ethylene glycol	298.15	1	263 at 298.15 K	—	—	8.10	Wang et al. (2022b)
[DBUH][Thy]:EG	1,8-diazabicyclo- [5,4,0]undec-7-ene thymol	1:2	Ethylene glycol	298.15	1	477 at 298.15 K	—	—	6.82	Wang et al. (2022b)

Ahmad et al. (2021) synthesized the MDEA-based DESs using [DEAHCl], [MDEAHCl], and [MEAHCl] as HBA and [MDEA] as HBD at 1:3 M ratio, and the CO_2_ solubility (mol/kg) followed the order of ∼2.63 > 2.46 > 1.35 at 298.15 K and 1 bar for the following DESs, [MEAHCl][MDEA], [DEAHCl][MDEA], and [MDEAHCl][MDEA]. The viscosity of the prepared DESs was in the range of 200–300 cP, while after CO_2_ absorption, it was increased up to ∼1,500 cP for the first two DES and ∼410 cP for the last one. Fu et al. (2021a) synthesized various types of DESs using 1,8-Diazabicyclo [5.4.0] undec-7-ene (DBU) combined with pyrrolidine, 2-pyrrolidinone, oxazolidinone, and ethyleneurea, and their solubilities (mol/kg) followed the order of [DBU][Pyr] (2:1) > [DBU][Eth] (2:1) > [DBU][Py] > (1:1) > [DNU][Oxa] (2:1), i.e., 8.2 > 7.5 > 6.5 > 6.3 mol/kg at 303 K and 1 bar. Qian et al. (2022) synthesized DESs using glycine and proline as HBA, and monoethanolamine and methyl diethanolamine as HBD with 1:4 M ratio for the post-combustion CO_2_ capture. The solubility (mol/kg) decreased in the following order: [Gly][MEA] > [Pro][MEA] > [Pro][MDEA], i.e., 5.2 > 4.6 > 1.1 mol/kg, at 298.15 K and 1.01 bar.

Apart from two-component DESs, Qian et al. (2022) synthesized three-component ones using amino acids as HBA, amines as HBDs, and 10–30 wt% of ethylene glycol. In this case, the CO_2_ solubility reached up to 5 mol/kg. Some DESs have been made by mixing ILs ethylene glycol and 4-methylimidazole (Fu et al., 2021b; Wang et al., 2021c; Lee et al., 2021; Wang et al., 2022a).

In summary, most of the work since 2021 is mainly on the synthesis of chemical-based ILs. Also, a great effort has been devoted to the imidazulim-, phosphonium- and ammonium-based ILs, which were previously synthesized. Among various physical-based ILs, [BVIM][Tf_2_N] showed the highest capacity of 6.3 mol/kg at 303.2 K and 71.1 bar. For the chemical-based ILs, [DETAH][Im] exhibited the highest solubility of 11.9 mol/kg at 313.15 K and 1 bar. For CO_2_ capture by DESs, choline-, ammonium-, phosphonium-, imidazolium-based solvents have been widely considered. In terms of the physical-based DESs, the highest solubility reached 9.7 mol/kg for [EAHC][TEPA] at 303.15 K and 16.03 bar [DBU][EG] captured CO_2_ with the highest capacity up to 12.5 mol/kg at 298.15 K and 1 bar as the chemical-based DES. Even though DESs show more promising compared to ILs, the research work is still relatively less. More focus needs to be on DESs.

### 3.3 Data collection, properties prediction, and ionic liquids/deep eutectic solvents screening

Based on the information from the previous reviews published in recent years as well as the new ILs and DESs that have been surveyed in this work, the available ILs and DESs that have been developed are summarized in Tables 1–4; Supplementary Table S1–S2. According to the survey and collection, the CO_2_ solubility is the main concern during the development, while the viscosity, selectivity and/or heat of absorption may also be covered, which are also essential for developing different IL/DES-based technologies.

Based on the collected ILs and DESs, promising ones can be suggested for different IL/DES-based technologies based on different criteria. In this work, the screening was conducted based on two options, according to the characterizations of different IL/DES-based technologies for CO_2_ separation.

#### 3.3.1 Option 1: Top 10 ionic liquids/deep eutectic solvents based on the CO_2_ absorption capacity

For the technology of immobilization, i.e., nano-confined ILs/DESs for CO_2_ capture, the CO_2_ absorption capacity is essential, while the challenges related to viscosity and selectivity can be mitigated compared to the technologies with bulk ILs/DESs. Therefore, according to the results listed in Tables 1–4; Supplementary Table S1–S2, top 10 ILs/DESs based on CO_2_ absorption capacity were screened from all the studied ILs/DESs for each type, i.e., physical or chemical-based. The screened ILs/DESs are listed in Table 5.

**TABLE 5:** 10 physical- and chemical-based ILs and DESs with high CO_2_ absorption capacity.

ILs			
Physical-based	Absorption capacity (mol/kg)	Chemical-based	Absorption capacity (mol/kg)
[BVIM][Tf_2_N] Yim et al. (2021)	6.268	[DETAH][Im] Wu et al. (2019)	11.91
[P_4441_][Tf_2_N] Yim et al. (2021)	6.063	[DETAH][Py] Wu et al. (2019)	11.39
[HMIM][FAP] Wu et al. (2021)	∼5.791	[DETAH][Gly] Wu et al. (2019)	10.15
[BMIM][FAP] Wu et al. (2021)	∼4.628	[DETAH][Tz] Wu et al. (2019)	10.10
[BZMIM][Tf_2_N] Jalili et al. (2022)	4.23	[TETAH][Lys] Jing et al. (2018)	9.72
[BMIM][BF_4_] Jiang et al. (2019b)	4.2	[MOBMIM][Gly] Qu et al. (2021)	9.66
[EMIM][FAP] Wu et al. (2021)	∼4.195	[EMIM][Gly] Min et al. (2021)	8.645
[N_4222_][Tf_2_N] Yim et al. (2021)	4.180	[DETAH][Lys] Jing et al. (2018)	7.68
[AMIM][Tf_2_N] Taheri et al. (2018)	3.88	[BMIM][Gly] Qu et al. (2021)	7.251
[DEA][Bu] Alcantara et al. (2018)	3.71	[MOBMIM][His] Qu et al. (2021)	7.026
**DESs**			
[EAHC][TEPA] (1:9) Pishro et al. (2021)	9.671	[TBD][EG] (1:4) García-Argüelles et al. (2017)	12.9
[TPPB][DEG] (1:4) Ghaedi et al. (2017)	7.29	[DBU][EG] (1:4) García-Argüelles et al. (2017)	12.48
[ATPPB][TEG] (1:4) Ghaedi et al. (2017)	6.42	[ChCl][U]/AMP (1:2) Uma Maheswari and Palanivelu (2015)	11
[TPPB][DEG] (1:10) Ghaedi et al. (2017)	5.79	[ChCl][TEG]/AMP (1:4) Uma Maheswari and Palanivelu (2015)	10.51
[TPPB][DEG] (1:16) Ghaedi et al. (2017)	5.24	[ChCl][DEG]/AMP (1:4) Uma Maheswari and Palanivelu (2015)	10.36
[L-Arg][GLY] (1:6) Ren et al. (2018)	4.92	[ChCl][U]/MAE (1:2) Uma Maheswari and Palanivelu (2015)	9.97
[ATPPB][TEG] (1:10) Ghaedi et al. (2017)	4.77	[ChCl][U]/MEA (1:2) Uma Maheswari and Palanivelu (2015)	9.84
[ChCl][La] (1:1) Altamash et al. (2020)	4.52	[DBUH][Thy]/EG (1:4) Wang et al. (2022b)	9.08
[Al][La] (1:1) Altamash et al. (2020)	4.30	[DBUH][Car]/EG (1:4) Wang et al. (2022b)	8.99
[ATPPB][TEG] (1:16) Ghaedi et al. (2017)	4.29	[DBN][BMIMCl][Im] (1:1:2) Zhang et al. (2019)	8.92

#### 3.3.2 Option 2: Top 10 ionic liquids/deep eutectic solvents based on a set of properties

In this option, top 10 ILs/DESs were screened based on a set of properties, including CO_2_ absorption capacity, viscosity, and selectivity/heat of absorption. For the solvent-based technologies with ILs/DESs, desirable properties more than CO_2_ absorption capacity are needed. However, not all the properties are available for the collected ILs/DESs. To achieve the screening, the work was conducted step by step. Step 1: top 20 ILs/DESs were screened based on the CO_2_ absorption capacity, and other properties of these top 20 ILs/DESs were combined or predicted with COSMO-RS; Step 2: top 10 ILs/DESs were further screened based on the different sets of parameters, depending on the feature of ILs/DESs (chemical-based or physical-based).

##### 3.3.2.1 Properties for the top 20 ionic liquids/deep eutectic solvents

According to the CO_2_ absorption capacity of ILs and DESs collected in Tables 1–4; Supplementary Table S1–S2, top 20 ILs/DESs were selected. The chosen 20 ILs/DESs are listed in Supplementary Table S3–S6.

To conduct screening, as most important properties other than CO_2_ absorption capacity are unavailable, theoretical predictions can be desirable. The COSMO-RS model, as a prior method, has been widely used to predict a variety of thermodynamic properties, such as the phase equilibrium, saturated vapor pressure, infinite dilution activity coefficient of systems containing ILs and DESs, and identify molecular mechanisms in chemical separation and reaction processes (Klamt, 1995; Zhou et al., 2012; Klamt et al., 2016; Yu et al., 2022), and the previous research has evidenced the reliability of COSMO-RS in screening ILs and DESs (Palomar et al., 2011; Sistla and Khanna, 2011; Hadj-Kali et al., 2020). In this work, the COSMO-RS model was employed to evaluate the CO_2_ solubility, selectivity of CO_2_ to other gases (i.e., CO, H_2_, CH_4_, and N_2_) and CO_2_ excess enthalpy in ILs and DESs as well as the viscosity of ILs by means of the COSMOThermX19 software. The selectivity of different gases was obtained from the corresponding Henry’s constants.

In the COSMO-RS calculation, the molecular geometry structures of HBAs and HBDs in DESs as well as cations and anions in ILs were optimized based on the density functional theory (DFT) at the calculation level of B3LYP/6–31+G (d, p) (Curtiss et al., 1995) by using the Gaussian 09 program (Frisch et al., 2009). Then, the COSMO file was obtained based on the most stable geometrical configuration by carrying out the calculation of COSMO continuum solution models at the theoretical level of BP86/TZVP (Perdew, 1986; Schäfer et al., 1994).

**FIGURE 1:**
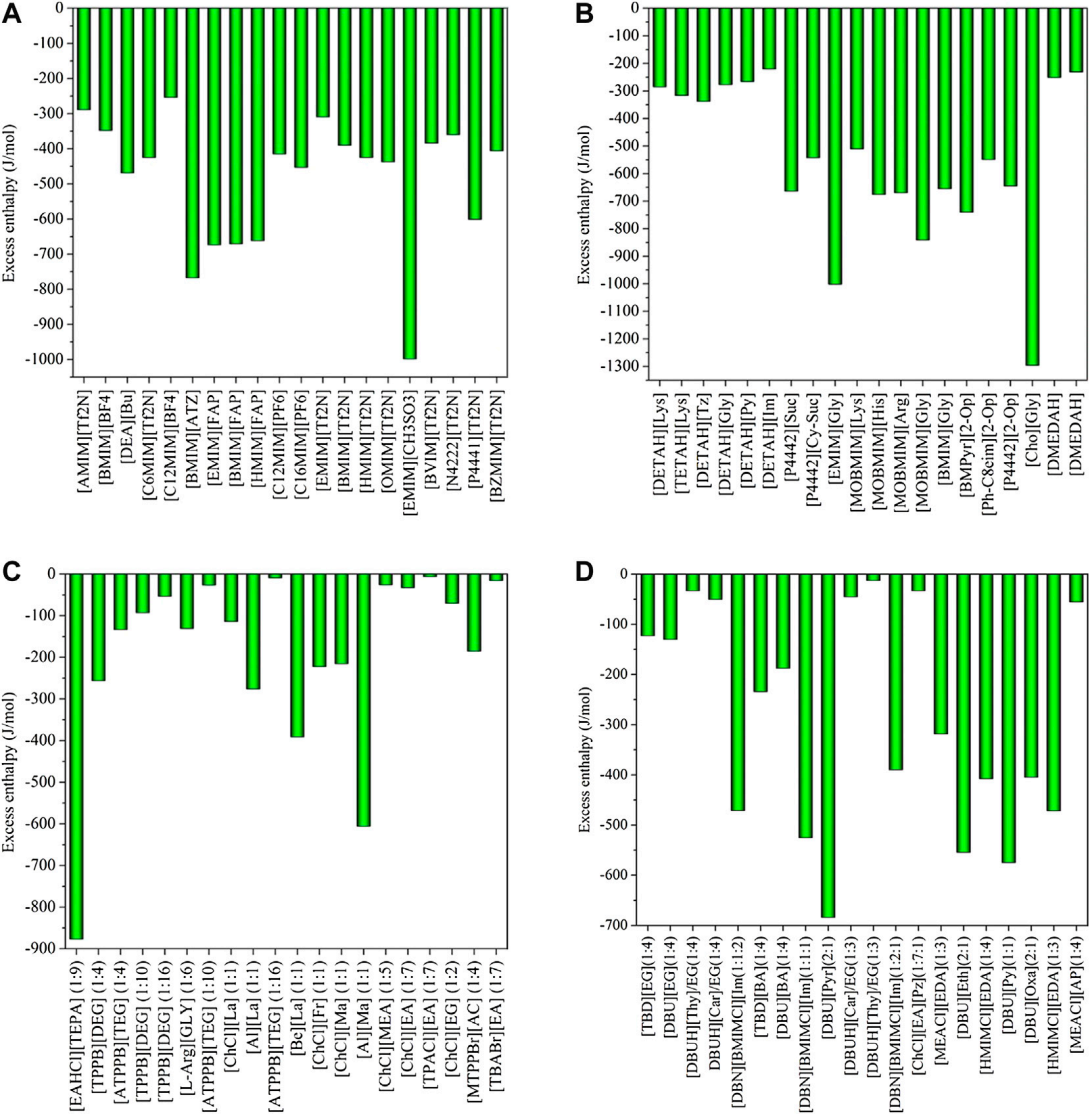
Excess enthalpies of equimolar CO_2_ in physical-based ILs **(A)**, chemical-based ILs **(B)**, physical-based DESs **(C)** and chemical-based DESs **(D)** predicted by the COSMO-RS model at 298.15 (K)

**FIGURE 2:**
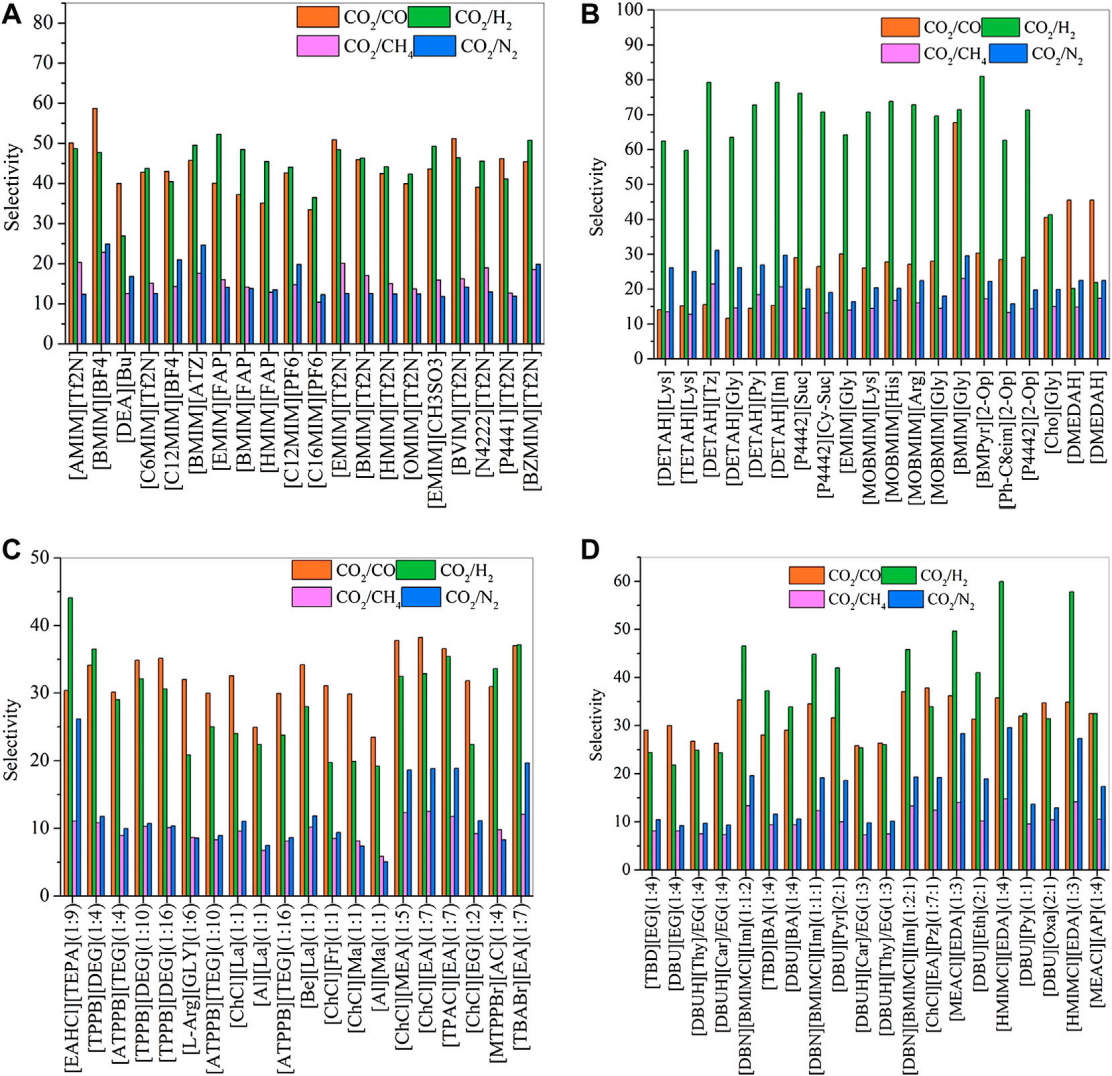
Selectivities of CO_2_/CO, CO_2_/H_2_, CO_2_/CH_4,_ and CO_2_/N_2_ in physical-based ILs **(A)**, chemical-based ILs **(B)**, physical-based DESs **(C)** and chemical-based DESs **(D)** predicted by the COSMO-RS model at 298.15 (K)

**FIGURE 3:**
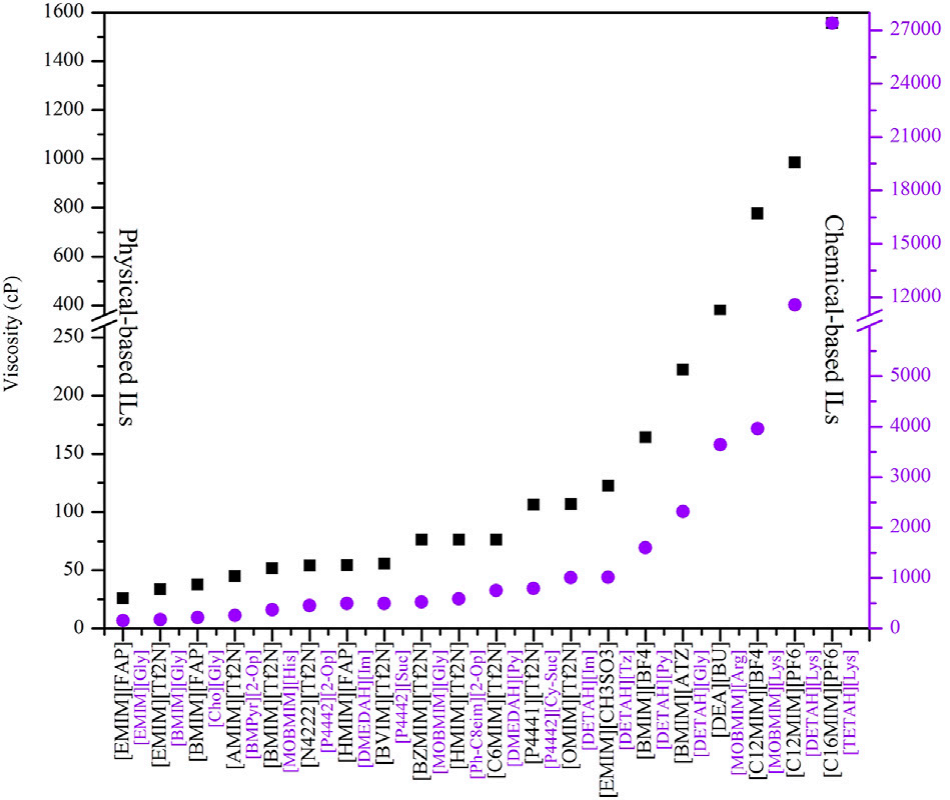
Viscosity of chemical- and physical-based ILs predicted by COSMO-RS.

Using COSMO-RS, the viscosity, selectivity, and CO_2_ excess enthalpy in ILs and DESs were estimated for the top 20 ILs/DESs. The results are summarized in Supplementary Table S7–S8, and depicted in Figures 1–3. Here, it should be mentioned that COSMO-RS cannot be used to predict the viscosities of DESs.

Generally, CO_2_ excess enthalpy and selectivity for physical-based ILs/DESs should be lower than those of chemical-based. According to the prediction results of COSMO-RS (Figure 1), some physical-based ILs/DESs show even higher CO_2_ excess enthalpies than those of chemical-based, and the selectivity of chemical-based ILs/DESs is not as high as we expected (Figure 2), especially for CO_2_ over CO and CH_4_ in chemical-based ILs. These observations indicate that the qualitative prediction of COSMO-RS may need to be verified and further improved. However, no work has been conducted to study the selectively for the chemical-based ILs/DESs, and the reliability of COSMO-RS prediction needs to be further verified. As shown in Figure 3, the viscosity of the chemical-based ILs is in general higher than the physical-based, which is consistent with the observation from experiments. For almost all the top 20 ILs/DESs, the properties other than CO_2_ absorption capacity were not determined experimentally. In this work, COSMO-RS was thus used for a rough estimation for qualitative comparison. It should be mentioned here that, the molar ratios of some physical-based DESs were not mentioned in the literature, and thus the prediction with COSMO-RS cannot be performed. In this case, other DESs were added in the top 20 list to facilitate the utilization of COSMO-RS (see Supplementary Table S6).

##### 3.3.2.2 Top 10 ionic liquids/deep eutectic solvents

As discussed in the previous section, the CO_2_ absorption capacity (mass basis), viscosity, and selectivity were used as the criteria to screen physical-based ILs/DESs, while the absorption capacity (mass basis), viscosity (before and after CO_2_ absorption), and CO_2_ absorption enthalpy were needed to screening chemical-based ILs/DESs. However, the viscosities of most DESs are not accessible, and it was excluded in the DES screening in this section. For the chemical-based ILs/DESs, the viscosity after CO_2_ absorption is also important, which again is inaccessible for most ILs/DESs. To address this, CO_2_ excess enthalpy in ILs and DESs was used as the index to reflect this. Also, CO_2_ absorption capacity is another important issue, which was also reflected with the CO_2_ excess enthalpy in ILs/DESs. Therefore, the top 20 ILs/DESs were further evaluated.

**FIGURE 4:**
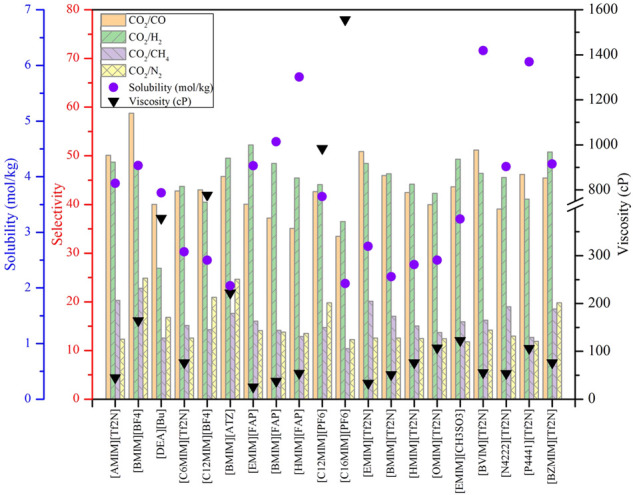
Top 20 physical-based ILs based on their properties.

**FIGURE 5:**
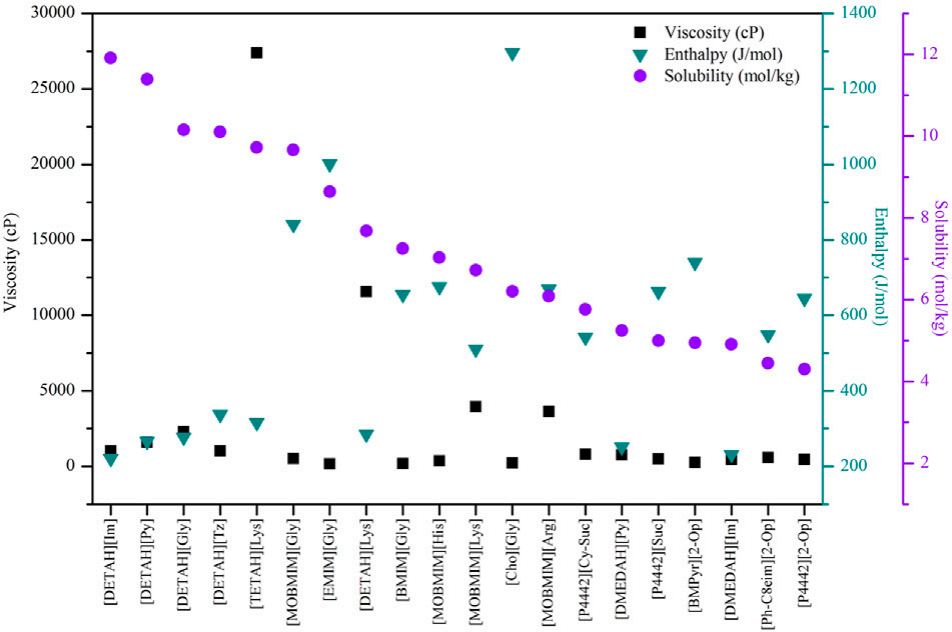
Top 20 chemical-based ILs based on their properties.

**FIGURE 6:**
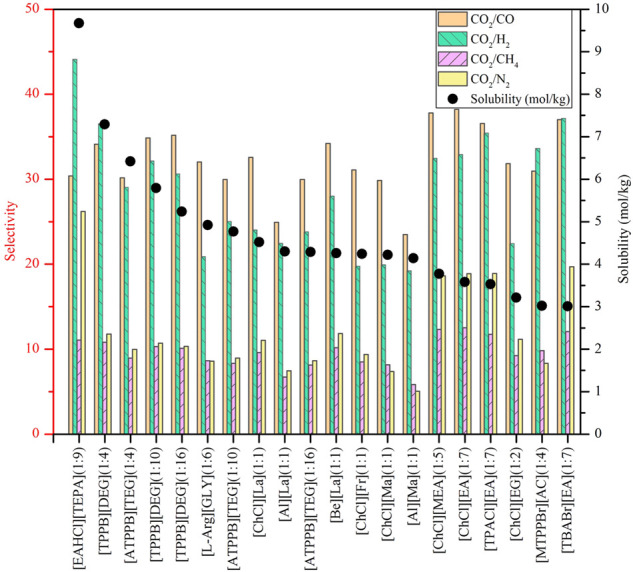
Top 20 physical-based DESs based on their properties.

**FIGURE 7:**
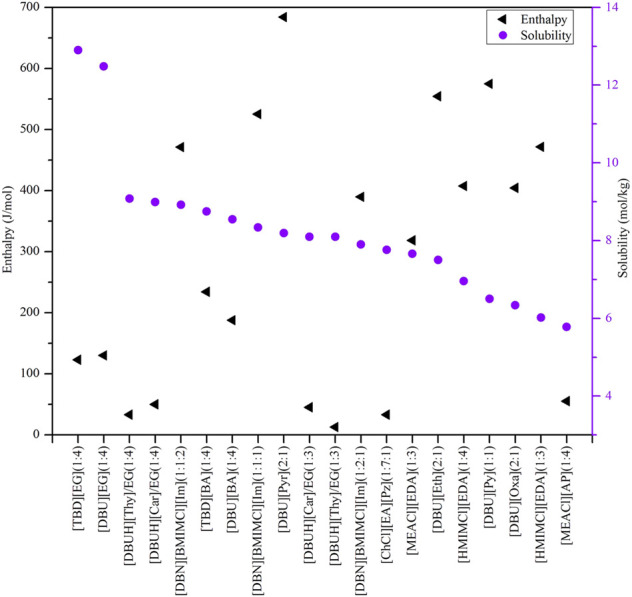
Top 20 chemical-based DESs based on their properties.

According to the results depicted in Figures 4–7, top 10 ILs/DESs were screened, and the results are summarized in Table 6.

**TABLE 6:** Suggested top 10 ILs/DESs.

ILs		DESs	
Physical-based	**Chemical-based**	**Physical based**	**Chemical-based**
**[BVIM][Tf**_**2**_ **N]**	[DETAH][Im]	[EAHC][TEPA] (1:9)	TBD-EG (1:4)
**[HMIM][FAP]**	[DETAH][Py]	[TPPB][DEG] (1:4)	DBU-EG (1:4)
**[P**_**4441**_ **][Tf**_**2**_ **N]**	[DETAH][Gly]	[ATPPB][TEG] (1:4)	[DBUH][Thy]/EG (1:4)
**[BMIM][FAP]**	[DETAH][Tz]	[TPPB][DEG] (1:10)	[DBUH][Thy]/EG (1:3)
**[EMIM][FAP]**	[MOBMIM][Gly]	[TPPB][DEG] (1:16)	[DBUH][Car]/EG (1:4)
**[N**_**4222**_ **][Tf**_**2**_ **N]**	[BMIM][Gly]	[L-Arg][GLY] (1:6)	[ChCl][EA][Pz] (1:7:1)
**[AMIM][Tf**_**2**_ **N]**	[MOBMIM][His]	[ATPPB][TEG] (1:10)	[DBUH][Car]/EG (1:3)
**[BZMIM][Tf**_**2**_ **N]**	[DMEDAH][Py]	[ChCl][La] (1:1)	[DBUH][BA] (1:4)
**[BMIM**[**BF**_**4**_ **]**	[DMEDAH][Im]	[Be][La] (1:1)	[TBD][BA] (1:4)
**[EMIM][CH**_**3**_ **SO**_**3**_ **]**	[P_4442_][Cy-Suc]	[ChCl][Fr] (1:1)	[MEACl][AP] (1:4)

## 4 Conclusion

Owing to the unique properties of ILs and DESs, intensive research has been conducted to develop such novel liquids for CO_2_ capture. In this work, different IL/DES-based technologies were summarized, and the important properties of each technology were identified to provide a guideline for screening the desirable ILs/DESs. Subsequently, the ILs and DESs studied in the recent 2 years were surveyed and then combined with those collected in the previous review articles to provide an extensive database. Furthermore, when the experimental data was not available, COSMO-RS was used to predict the properties that are needed for screening. Based on the established database, the identified important properties, and the predictions from the COSMO-RS, top 10 ILs/DESs were screened for different IL/DES-based technologies, being beneficial for further evaluation and development.
