# Supplementary material for: Reviewing and screening ionic liquids and deep eutectic solvents for effective CO2 capture
Source: Front Chem. 2022 Aug 10;10:951951. doi: 10.3389/fchem.2022.951951 (PMC9399623; doi:10.3389/fchem.2022.951951)
Supplement: Supplementary file 1 [file DataSheet1.docx]

**Supplementary tables:**

**Table S1.** Physical- and chemical-based ILs.

| Abbreviation | Full name | Mw (g/mol) | *T* (K) | *P* (bar) | Absorption capacity (mol/kg) | Ref. |
| --- | --- | --- | --- | --- | --- | --- |
|  |  |  |  |  |  |  |
| Physical-based ILs | | | | | | |
| [AMIM][Tf_2_N] | 1-allyl-3-methylimidazolium bis(trifluoromethyl sulfonyl)imide | 404.33 | 313.2-353.2 | 9.5-58.3 | 0.27-3.88 | (Taheri et al., 2018) |
| [EMIM][ATZ] | 1-ethyl-3-methylimidazolium 3-amino-1h-1,2,4-triazolate | 194.24 | 293.15, 298.15 | 1 | 0.67 | (Zhang et al., 2019b) |
| [EMIM][TCM] | 1-ethyl-3-methylimidazolium tricyanomethanide | 201.23 | 298.2-373.2 | 0.494-3.063 | 0.016-0.3 | (Huang and Peng, 2017) |
| [BMIM][BF_4_] | 1-butyl-3-methylimidazolium tetrafluoroborate | 226.02 | 273.15-353.15 | 5.28-34.38 | 2.49-4.2 | (Jiang et al., 2019b) |
| [DEA][Bu] | Diethylammonium butanoate | 161.29 | 303-333 | 7.25-196.70 | 0.63-3.71 | (Alcantara et al., 2018) |
| [EMIM][Ac] | 1-ethyl-3-methylimidazolium acetate | 170.20 | 298 | 0.06-9.94 | 0.0043-0.78 | (Altamash et al., 2017) |
| [TBMP][Formate] | Tributylmethylphosphonium formate | 262.30 | 298 | 0.06-9.90 | 0.004-0.78 | (Altamash et al., 2017) |
| [C_6_MIM][Tf_2_N] | 1-hexyl-3-methyl-imidazolium bis(trifluoromethylsulfonyl) amide | 447.42 | 303.15-353.15 | 0-35.555 | 0-2.65 | (Jalili et al., 2017) |
| [BMIM][OTf] | 1-butyl-3-methylimidazolium trifluoromethanesulfonate | 288.29 | 303.15 | 0-31.88 | 0-2.4809 ± 0.1881 | (Jalili et al., 2017) |
| [C_12_MIM][PF_6_] | 1-dodecyl-3-methyl-imidazolium hexafluorophosphate | 396.40 | 333.15-373.15 | 9.90-57.90 | 0.22-2.04 | (Dai et al., 2017) |
| [C_12_MIM][BF_4_] | 1-dodecyl-3-methyl-imidazolium tetrafluoroborate | 338.24 | 333.15-373.15 | 9.40-57.30 | 0.29-2.5 | (Dai et al., 2017) |
| [C_12_MIM][Tf_2_N] | 1-dodecyl-3-methyl-imidazolium tetrafluoroborate | 531.58 | 333.15-373.15 | 7.6-56.7 | 0.18-1.97 | (Dai et al., 2017) |
| [BMIM][ATZ] | 1-butyl-3-methylimidazolium 3-amino-1h-1,2,4-triazolate | 222.29 | 298.15 | 1 | 2.04 ± 0.06 | (Zhang et al., 2019b) |
| [BMIM][Tf_2_N] | 1-butyl-3-methylimidazolium bis(trifluoromethylsulfonyl)imide | - | 298.15 | 11.63 | 1.4 | (Aki et al., 2004) |
| [HMIM][Tf_2_N] | 1-hexyl-3-methylimidazolium bis(trifluoromethylsulfonyl)imide | - | 298.15 | 13.15 | 1.313 | (Aki et al., 2004) |
| [BMIM][PF_6_] | 1-butyl-3-methylimidazolium hexafluorophosphate | - | 298.15 | 13.31 | 1.236 | (Aki et al., 2004) |
| [BMIM][BF_4_] | 1-butyl-3-methylimidazolium tetrafluoroborate | - | 298.15 | 12.09 | 1.397 | (Aki et al., 2004) |
| [N_1114_][Tf_2_N] | Butyltrimethylammonium bis- (trifluoromethyl sulfonyl) imide | 396.37 | 298 | 0.06-9.95 | 0.004-0.61 | (Altamash et al., 2017) |
| [BMIM][NO_3_] | 1-butyl-3-methylimidazolium nitrate | - | 298 | 10.31 | 0.614 | (Aki et al., 2004) |
| [PMPy][DCA] | 1-methyl-1- propylpyrrolidinium dicyanamide | 194.23 | 298 | 0.06-9.94 | 0.0044-0.71 | (Altamash et al., 2017) |
| [EIMH][CuCl_2_] | 1-ethylimidazolium chlorocuprate | - | 303.2 | 1 | ~0.022 | (Liu et al., 2019) |
| [EMIM][CuCl_2_] | 1-ethyl-3-methylimidazolium  chlorocuprate | - | 303.2 | 1 | ~0.073 |  |
| [EMIM][SCN] | 1-ethyl-3-methylimidazolium thiocyanate | - | 298.2-373.2 | 0.528-3.08 | 0.0059-0.11 | (Huang and Peng, 2017) |
| [EMIM][DCA] | 1-ethyl-3-methylimidazolium dicyanamide | - | 298.2-373.2 | 0.521-3.05 | 0.0096-0.17 |  |
| [P_66614_]_2_[CoCl_4_] | Trihexyl(tetradecyl)phosphonium tretrachlorocobalt | 1112 | 298.15 | 1 | 0.0827 | (Santos et al., 2014) |
| [P_66614_][FeCl_4_] | Trihexyl(tetradecyl)phosphonium tretrachloroferrate | 681.51 |  |  | 0.105 |  |
| [P_66614_]_2_[MnCl_4_] | Trihexyl(tetradecyl)phosphonium tretra-chloromanganese | 1103 |  |  | 0.0752 |  |
| [P_66614_]_3_[GdCl_6_] | Trihexyl(tetradecyl)phosphonium hexa-chlorogadolinium | 1821.54 |  |  | 0.052 |  |
| [(2,2)O_Et_Im][Tf_2_N]_2_ | 1,1′,3,3′-bis(3,6-dioxaoctane-1,8-diyl)bis(imidazolium) bis(trifluoromethanesulfonyl)imide | - | 298.15 | 10 | 0.368 | (Thapaliya et al., 2021) |
| [(3,3)O_Et_Im][Tf_2_N]_2_ | 1,1′,3,3′-bis(3,6,9-trioxaundecane-1,11-diyl)bis(imidazolium) bis(trifluoromethanesulfonyl)imide | - |  |  | 0.433 |  |
| [(3,3)O_Et_DABCO][Tf_2_N]_4_ | 1,1′,4,4′-bis(3,6,9-trioxaundecane-1,11-diyl)bis(1,4-diazoniabicyclo[2.2.2]octane) bis(trifluoromethanesulfonyl)imide | - |  |  | 0.261 |  |
| [(3,3)O_Et_DABCO][BETI]_4_ | 1,1′,4,4′-bis(3,6,9-trioxaundecane-1,11-diyl)bis(1,4-diazoniabicyclo[2.2.2]octane) bis(pentafluoroethanesulfonyl)imide | - |  |  | 0.193 |  |
| [bis(octamethylene)-bis(imidazolium)][Tf_2_N]_2_ | 1,1′,3,3′-bis(octamethylene)bis(imidazolium) bis(trifluoromethanesulfonyl)imide – [bis(octamethylene)-bis(imidazolium)][Tf_2_N]_2_ | - |  |  | 0.382 |  |
| [bis(decamethylene)-bis(imidazolium)][Tf_2_N]_2_ | 1,1′,3,3′-bis(decamethylene)bis(imidazolium) bis(trifluoromethanesulfonyl)imide – [bis(decamethylene)-bis(imidazolium)][Tf_2_N]_2_ | - |  |  | 0.302 |  |
| Chemical-based ILs | | | | | | |
| [DETAH][Lys] | Diethylenetriammonium lysinate | 277.39 | 313.15 | 1 | 7.68 | (Jing et al., 2018) |
| [TETAH][Lys] | Triethylenetetrammonium lysinate | 266.43 |  | 1 | 9.72 |  |
| [DETAH][Tz] | Diethylenetriammonium 1,2,4-triazolide | 172.23 | 313.15 | 1 | 10.10 | (Wu et al., 2019) |
| [DETAH][Gly] | Diethylenetriammonium glycinate | 178.24 |  | 1 | 10.15 |  |
| [DETAH][Py] | Diethylenetriammonium pyrazolide | 171.25 |  | 1 | 11.39 |  |
| [DETAH][Im] | Diethylenetriammonium imidazolide | 171.25 |  | 1 | 11.91 |  |
| [P_4442_][Suc] | Tri-n-butylphosphine succinimide | 329.46 | 293.15, 303.15 | 0.1 | 5, 3.4 | (Huang et al., 2017) |
| [P_4442_][Suc] | Tri-n-butylphosphine succinimide | 329.46 | 293.15 | 1 | 5.62 | (Huang et al., 2018) |
| [P_4442_][Cy-Suc] | Tri-n-butylphosphine 1,2-cyclohex-  anedicarboximide | 383.56 | 293.15 | 1 | 5.76 |  |
| [EMIM][Gly] | 1-ethyl-3- methylimidazolium glycinate |  | 298.15 | - | 8.645 | (Min et al., 2021) |
| [MOBMIM][Lys] | 1-methoxylbutyl-3-methylimidazolium lysine |  | 323.15 | 5 | 6.721 | (Qu et al., 2021) |
| [MOBMIM][His] | 1-methoxylbutyl-3-methylimidazolium histidine |  |  | 5 | 7.026 |  |
| [MOBMIM][Arg] | 1-methoxylbutyl-3-methylimidazolium arginine |  |  | 5 | 6.086 |  |
| [MOBMIM][Gly] | 1-methoxylbutyl-3-methylimidazolium glycine |  |  | 5 | 9.66 |  |
| [BMIM][Gly] | 1-butyl-3-methylimidazolium glycine | 213.28 |  | 5 | 7.251 |  |
| [P_66614_]_2_[Asp] | - |  | 303.15 | 1 | 2.587 | (Luo et al., 2019b) |
| [K(AMP)_2_][Im] | - |  | 332.2 | 1 | 4.183 | (Zema et al., 2021) |
| [BMPyr][2-Op] | - | 236.36 | 303.15 | 1 | 4.95 | (Luo et al., 2019a) |
| [Ph-C_8_eim][2-Op] | 1-n-ethyl-3-n-octyl-2-phenylimidazolium 2- hydroxyl pyridium | 379.54 | 293.15 | 1 | 4.45 | (Luo et al., 2019a) |
| [P_4442_][2-Op] | Tributyl(ethyl)phosphonium 2-hydroxylpyridium | 325.47 | 303.15 | 1 | 4.30 | (Luo et al., 2019a) |
| [P_2228_][6BrInda] | Triethyl (octyl) phosphonium 6-bromoindazolide | - | 332 | 0.833 | 2.34 | (Keller et al., 2022) |
| [EMIM][Ac] | 1-ethyl-3-methylimidazolium acetate | 170.2 | 313.2 | 1 | 1.65 | (Chen et al., 2018) |
| [EMIM][Ala] | 1-ethyl-3-methylimidazolium alaninate | 200.26 |  | 1 | 1.89 |  |
| [EMIM][Gly] | 1-ethyl-3-methylimidazolium glycinate | 186.23 |  | 1 | 2.32 |  |
| [DMAPAH][2F-PhO] | N,N-dimethyl-1,3-propane [diamine](https://www.sciencedirect.com/topics/chemistry/diamine) 2-fluorophenolate | 214.2 | 303.2 | 1 | 3.12 | (Zhao et al., 2018) |
| [DMAPAH][3F-PhO] | N,N-dimethyl-1,3-propane diamine 3-fluorophenolate | 214.4 |  | 1 | 3.39 |  |
| [DMAPAH][3,5F-PhO] | N,N-dimethyl-1,3-propane diamine 3,5-difluorophenolate | 232.3 |  | 1 | 3.52 |  |
| [DMAPAH][4F-PhO] | N,N-dimethyl-1,3-propane diamine 3-fluorophenolate | 214.4 |  | 1 | 3.99 |  |
| [DMEDAH][Formate] (0.5:1-2.5:1) | N,N-dimethylethylenediammonium formate | 60.07-76.12 | 298.15 | 1 | 0.65-5.16 | (Vijayaraghavan et al., 2018) |
| [DMAPAH][Formate] (0.5:1-2.5:1) | 3-(dimethylamino)-1-propylammonium formate | 64.74-86.14 |  | 1 | 0.70-2.58 |  |
| [DMAPAH][Octanoate] (0.5:1-2.5:1) | 3-(dimethylamino)-1-propylammonium octanoate | 114.19-130.20 |  | 1 | 1.02-4.17 |  |
| [N_1111_][Lys] | Tetramethylammonium lysinate | 219.11 | 303 | 1 | 1.84 | (Meng et al., 2018) |
| [P_2228_][6-BrBnIm] | Triethyl(octyl)phosphonium 6-bromobenzimidazolide | 428.42 | 313.15 | 0-149.9 | 0-2.01 | (Song et al., 2019) |
| [P_2228_][BnIm] | Triethyl(octyl)phosphonium benzimidazolide | 349.52 | 298.15-333.15 | 0-99.8 | 0-2.78 |  |
| [P_2228_][2-CNPyr] | Triethyl(octyl)phosphonium 2-cyanopyrrolide | 323.48 | 298.15-333.15 | 0-99.8 | 0-2.84 |  |
| [BMIM][2-Op] | 1-butyl-3-methylimidazolium 2-hydroxylpyridium | 233.31 | 303.15 | 1 | 4.37 | (Luo et al., 2019a) |
| [N_4442_][2-Op] | Tributyl(ethyl)ammonium 2-hydroxylpyridium | 236.36 |  | 1 | 4.02 |  |
| [P_4442_OH][2-Op] | Tributyl(ethoxyl)phosphonium 2-hydroxylpyridium | 308.51 |  | 1 | 2.75 |  |
| [P_4442_][DAA] | Tri-n-butyl(ethyl)phosphonium diacetamide | 331.48 | 293.15 | 0.1 | 3.37 | (Huang et al., 2017) |
| [P_4442_][Ph-Suc] | Tri-n-butyl(ethyl)phosphonium o-phthalimide | 329.47 | 293.15 | 1 | 4.25 | (Huang et al., 2018) |
| [P_66614_][Beta-Ala] | Trihexyl(tetradecyl)phosphonium bata-alaline | 572.96 | 303.15 | 1 | 1.74 | (Luo et al., 2019b) |
| [P_66614_]_2_[Asp] | - | 1068.83 |  | 1 | 1.83 |  |
| [P_66614_][MA-Tetz] | - | 582.96 |  | 1 | 1.94 |  |
| [TMGH][PhO] | Tetramethylgunidinium phenol | 209.29 | 313.15 | 1 | 0.24 | (Li et al., 2019) |
| [TMGH][Im] | Tetramethylgunidinium imidazole | 183.26 |  | 1 | 3.49 |  |
| [TMGH][Pyrr] | Tetramethylgunidinium pyrrole | 182.27 |  | 1 | 3.62 |  |
| [VBTMA][Ala] | Vinylbenzyltrimethylammonium alanine | 294.44 | 298 | 1 | 0.98 | (Shahrom et al., 2019) |
| [VBTMA][Pro] | Vinylbenzyltrimethylammonium proline | 320.47 |  | 1 | 1.19 |  |
| [VBTMA][Ser] | Vinylbenzyltrimethylammonium serine | 310.44 |  | 1 | 1.26 |  |
| [VBTMA][Hist] | Vinylbenzyltrimethylammonium histidine | 360.50 |  | 1 | 1.28 |  |
| [VBTMA][Tau] | Vinylbenzyltrimethylammonium taurine | 330.48 |  | 1 | 1.33 |  |
| [VBTMA][Gly] | Vinylbenzyltrimethylammonium glycinate | 280.41 |  | 1 | 1.68 |  |
| [VBTMA][Lys] | Vinylbenzyltrimethylammonium lysine | 351.53 |  | 1 | 1.88 |  |
| [VBTMA][Arg] | Vinylbenzyltrimethylammonium arginine | 379.55 |  | 1 | 2.19 |  |
| [AEMIM][BF_4_] | 3-aminoethyl-2-methyl-1-methylimidazolium tetrafluoroborate | - | 303.15 | 1 | 1.92  2.19 | (Sharma et al., 2012) |
| [AEMIM][DCA] | 3-aminoethyl-2-methyl-1-methylimidazolium dicyanamide | - |  | 1 |  |  |
| [N_2222_][Ala] | Tetraethylammonium alanine | - |  | 1 | ~2.06 | (Jiang et al., 2008) |
| [N_2222_][β-Ala] | Tetraethylammonium β-alanine | - | 313.15 | 1 | ~2.29 | (Yu et al., 2009) |
| [N_2224_][Ala] | Triethylbutylammonium alanine |  |  |  | ~1.95 |  |
| [P_66614_][Gly] | Trihexyl(tetradecyl)phosphonium glycine | - | 295.15 | 1 | 2.26 | (Goodrich et al., 2011) |
| [Me_2_N(CH_2_CH_2_OH)_2_][Tau] | Dihydroxyethyldimethylammonium taurinate | - | 310.15 | 4 | 3.56 | (Niedermaier et al., 2014) |
| [TMG][Im] | 1,1,3,3-tetramethylguanidinium imidazole | - | 303.15 | 1 | 5.46 | (Lei et al., 2014) |
| [P_2224_][2-CN-Pyr] | Triethyl(butyl)phosphonium 2-cyano-pyrrolide | - | 295.15 | 0.15 | 3 | (Seo et al., 2015) |
| [P_2228_][2-CN-Pyr] | Triethyl(octyl)phosphonium 2-cyano-pyrrolide |  |  | 0.15 | 2.45 |  |
| [MTBDH][TFE] | - | - | 296.15 | 1 | 4.45 | (Wang et al., 2010) |
| [DBUH][TFE] | 1,8-diazabicyclo[5.4.0]undec-7-ene trifluoroethanol | - | 298.15 | 1 | 4 | (Zhao et al., 2014) |
| [N_2222_][PhO] | Tetraethylammonium phenol | - | 323.15 | 1 | 2.86 | (Ren et al., 2013) |
| [BMIM][Im] | 1-butyl-3-methylimidazolium imidazolide | - | 313.15 | 1 | 2.62 | (Zhang et al., 2013) |
| [EMIM][Im] | 1-ethyl-3-methylimidazolium imidazolate |  |  |  | 3.03 |  |
| [HO-EMIM][Im] | 1-hydroxylethyl-3-methylimidazolium imidazolate |  |  |  | 2.83 |  |
| [N_66614_][Lys] | Trihexyl(tetradecyl)ammonium lysinate | - | 295.15 | 1 | 3.43 | (Saravanamurugan et al., 2014) |
| [N_66614_][Asn] | Trihexyl(tetradecyl)ammonium asparaginate |  |  |  | 3.34 |  |
| [N_66614_][Gln] | Trihexyl(tetradecyl)ammonium glutaminate |  |  |  | 3.10 |  |
| [N_66614_][His] | Trihexyl(tetradecyl)ammonium histidinate |  |  |  | 3.10 |  |
| [P_66614_][Lys] | Trihexyl(tetradecyl)phosphonium lysinate |  |  |  | 2.54 |  |
| [Bis(MIM)C_2_][Im]_2_ | 1,2-bis(3-methylimidazolium-1-yl)ethane imidazolate | - | 313.15 | 1 | 7.68 | (Zhang et al., 2013) |
| [Bis(MIM)C_4_][Im]_2_ | 1,4-bis(3-methylimidazolium-1-yl)butane imidazolate |  |  |  | 9.72 |  |
| [MTBDH]_2_[HFPD] | - | - | 296.15 | 1 | 3.96 | (Wang et al., 2010) |
| [P_2228_][CNPyr] | Triethyloctylphosphonium 2-cyanopyrrole | 322.47 | 313 | 10 | ~3.3 | (Hospital-Benito et al., 2020) |
| [P_66614_][CNPyr] | Trihexyltetradecylphosphonium 2-cyanopyrrole | 574.95 |  | 10 | ~1.8 |  |
| [BMIM][acetate] | 1-butyl-3-methylimidazolium acetate | 198.26 |  | 10 | ~2.6 |  |
| [BMIM][i-but] | 1-butyl-3-methylimidazolium isobutyrate | 226.32 |  | 10 | ~1.7 |  |
| [BMIM][GLY] | 1-butyl-3-methylimidazolium glycinate | 213.28 |  | 10 | ~1.3 |  |
| [BMIM][PRO] | 1-butyl-3-methylimidazolium prolinate | 253.34 |  | 10 | ~1.1 |  |
| [MEA][Im] | Monoethanolamine imidazole | - | 298.15 | 1 | 4.48 | (Mukesh et al., 2019) |
| [DETA]_2_[Im] | Diethylenetriamine imidazole | - |  |  | 2.96 |  |
| [TEPA]_2_[Im] | Tetraethylenepentamine imidazole | - |  |  | 2.38 |  |
| [HDBU][Im] | 1,8-diazabicyclo- [5,4,0]undec-7-ene imidazole | - | 313 | 1 | 4.41 | (Yan et al., 2020) |
| [HDBU][Ind] | 1,8- diazabicyclo[5,4,0]undec-7-ene indole | - | 313 | 1 | 3.02 | (Yan et al., 2020) |
| [HDBU][Triz] | 1,8-diazabicyclo[5,4,0]undec-7-ene 1,2,4-triazole | - | 313 | 1 | 1.73 | (Yan et al., 2020) |
| [DBUH][MLU] | 1,8-diazabicyclo[5.4.0]undec-7-ene methyl urea | - | 313 | 1 | 1.75 | (Fu et al., 2021b) |
| [DBUH]_2_[DMU] | 1,8-diazabicyclo[5.4.0]undec-7-ene 1,3-dimethylurea | - | 313 | 1 | 2.68 | (Fu et al., 2021b) |

**Table S2.** Physical- and chemical-based DESs.

| Abbreviation |  | Structure | | | *T* (K) | *P* (bar) | Absorption capacity (mol/kg) | Ref. |
| --- | --- | --- | --- | --- | --- | --- | --- | --- |
|  | **HBA** | | **HBD** | **Molar ratio (HBA:HBD)** |  |  |  |  |
| Physical-based DES | | | | | | | | |
| [Al][La] | Alanine | | Lactic acid | 1:1 | 298.15 | 50 | 4.30 |  |
| [Al][Ma] | Alanine | | Malic acid | 1:1 |  |  | 4.14 |  |
| [Be][La] | Betaine | | Lactic acid | 1:1 |  |  | 4.26 | (Altamash et al., 2020) |
| [ChCl][Fr] | Choline chloride | | Fructose | 1:1 |  |  | 4.24 |  |
| [ChCl][La] | Choline chloride | | Lactic acid | 1:1 |  |  | 4.52 |  |
| [ChCl][Ma] | Choline chloride | | Malic acid | 1:1 |  |  | 4.22 |  |
| [ChCl][EG] | Choline chloride | | Ethylene glycol | 1:2 | 303.15 | 58.63 | 3.216 | (Leron and Li, 2013b;a) |
|  |  |  |  |  | 313.15 | 59.02 | 2.614 |  |
|  |  |  |  |  | 323.15 | 61.67 | 2.1903 |  |
|  |  |  |  |  | 333.15 | 61.04 | 1.582 |  |
|  |  |  |  |  | 343.15 | 63.23 | 1.2168 |  |
| [ChC][Ph] | Choline chloride | | Phenol | 1:2 | 293.15 | 0.99 | 0.0349 | (Li et al., 2014) |
|  |  |  |  | 1:3 |  | 1.044 | 0.0412 |  |
|  |  |  |  | 1:4 |  | 1.082 | 0.0419 |  |
| [ChCl][DEG] |  |  | Diethylene glycol | 1:3 | 293.15 | 1.128 | 0.0321 |  |
|  |  |  |  | 1:4 |  | 1.104 | 0.0346 |  |
|  |  |  |  | 1:3 |  | 1.093 | 0.0357 |  |
| [ChCl][TEG] |  |  | Triethylene glycol | 1:4 | 293.15 | 1.19 | 0.0409 |  |
| [ChCl][LA] | Choline chloride | | Levulinic acid | 1:3 | 303.15 | 0.794 | 0.0351 | (Lu et al., 2015) |
|  |  |  |  | 1:4 |  | 0.725 | 0.0321 |  |
|  |  |  |  | 1:5 |  | 0.715 | 0.0339 |  |
| [ChCl][FA] |  |  | Furfuryl alcohol | 1:3 |  | 0.809 | 0.0263 |  |
|  |  |  |  | 1:4 | - | 0.825 | 0.0306 |  |
|  |  |  |  | 1:5 |  | 0.773 | 0.0300 |  |
| [MTPPBr][[MEA] | Methyltriphenylphosphonium bromide | | Monoethanolamine | 1:6 | 298.15 | 10 | 1.63 | (Ali et al., 2016) |
| [TBABr][MEA] | Tetrabutylammonium bromide | | Monoethanolamine | 1:6 |  |  | 1.34 |  |
| [MTPPBr][MEA] | Methyltriphenylphosphonium bromide | | Monoethanolamine | 1:7 |  |  | 1.46 |  |
| [MTPPBr][MEA] | Methyltriphenylphosphonium bromide | | Monoethanolamine | 1:8 |  |  | 1.44 |  |
| [ChCl][EA] | Choline chloride | | Ethanolamine | 1:7 | 298.15 | 1.82-20.35 | 0.78-3.58 | (Sarmad et al., 2017) |
| [GuaCl][EA] | Guanidinium chloride | | Ethanolamine | 1:2 |  | 2.26-20.25 | 0.31-1.66 |  |
| [BTMACl][AC] | Benzyltrimethylammonium chloride | | Acetic acid | 1:2 |  | 2.19-20.37 | 0.078-1.45 |  |
| [TBACl][AC] | Tetrabutylammonium chloride | | Acetic acid | 1:2 |  | 3.48-20.02 | 0.18-1.41 |  |
| [TEACl][OCT] | Tetraethylammonium chloride | | Octanoic acid | 1:3 |  | 3.53-20.18 | 0.16-1.39 |  |
| [MTPPBr][AC] | Methyltriphenylphosphonium bromide | | Acetic acid | 1:4 |  | 1.73-20.14 | 0.073-3.02 |  |
| [TBABr][EA] | Tetrabutylammonium bromide | | Ethanolamine | 1:6 |  | 3.51-20.21 | 0.44-2.78 |  |
| [TBABr][EA] | Tetrabutylammonium bromide | | Ethanolamine | 1:7 |  | 3.81-20.40 | 0.53-3.01 |  |
| [TMACl][AC] | Tetramethylammonium chloride | | Acetic acid | 1:4 |  | 2.94-20.96 | 0.12-1.56 |  |
| [TPACl][EA] | Tetrapropylammonium chloride | | Ethanolamine | 1:4 |  | 4.81-20.09 | 0.34-1.43 |  |
| [TPACl][AC] | Tetrapropylammonium chloride | | Acetic acid | 1:6 |  | 3.50-20.30 | 0.25-1.72 |  |
| [TPA]Cl-[EA] | Tetrapropylammonium chloride | | Ethanolamine | 1:7 |  | 3.57-20.19 | 1.71-3.53 |  |
| [ChCl][Urea] | Choline chloride | | Urea | 1:2 | 308.2-328.2 | 6.51-45.04 | 0.51-2.80 | (Xie et al., 2016) |
| [L-Arg][GLY] | L-arginine | | Glycerol | 1:6 | 333.15 | 1 | 4.92 | (Ren et al., 2018) |
| [N_4444_Cl][DECA] | Tetrabutylammonium chloride | | Decanoic acid | 1:2 | 298.15-323.15 | 0.9-19.90 | 0.042-1.52 | (Zubeir et al., 2018) |
| [N_8888_Cl][DECA] | Tetraoctylammonium chloride | | Decanoic acid | 1:1.5 | 298.15-323.15 | 0.9-19.90 | 0.041-1.41 |  |
| [N_8881_Cl][DECA] | Methyltrioctylammonium chloride | | Decanoic acid | 1:2 | 298.15-308.15 | 0.9-19.90 | 0.045-1.35 |  |
| [BTPPBr][EG] | Butyltriphenylphosphonium bromide | | Ethylene glycol | 1:12 | 298.15 | 10 | 0.60 | (Ali et al., 2016) |
| [BTPPCl][GLY] | Benzyltriphenylphosphonium chloride | | Glycerol | 1:12 |  |  | 0.47 |  |
| [TBABr][TEA] | Tetrabutylammonium bromide | | Triethanolamine | 1:3 |  |  | 0.47 |  |
| [TBABr][DEA] | Tetrabutylammonium bromide | | Diethanolamine | 1:6 |  |  | 0.85 |  |
| [BHDECl][LA] | N-benzyl-2-hydroxy-n,n-dimethyl ethanaminium chloride | | Lactic acid | 1:2 | 298.15 | 2.83-20.86 | 0.016-0.50 | (Sarmad et al., 2017) |
| [BHDECl][AC] | N-benzyl-2-hydroxy-n,n-dimethyl ethanaminium chloride | | Acetic acid | 1:2 |  | 2.1-20.26 | 0.064-0.84 |  |
| [BTEACl][AC] | Benzyltriethylammonium chloride | | Acetic acid | 1:2 |  | 3.25-20.54 | 0.13-0.97 |  |
| [BTMACl][GLY] | Benzyltrimethylammonium chloride | | Glycerol | 1:2 |  | 3.94-20.26 | 0.037-0.26 |  |
| [MTPPBr][EG] | Methyltriphenylphosphonium bromide | | Ethylene glycol | 1:3 |  | 1.92-20.18 | 0.045-0.35 |  |
| [MTPPBr][LEV] | Methyltriphenylphosphonium bromide | | Levulinic acid | 1:3 |  | 3.01-20.68 | 0.024-0.69 |  |
| [MTPPBr][GLY] | Methyltriphenylphosphonium bromide | | Glycerol | 1:4 |  | 1.61-20.26 | 0.009-0.29 |  |
| [MTPPBr][1,2-PRO] | Methyltriphenylphosphonium bromide | | 1,2-propanediol | 1:4 |  | 2.2-20.26 | 0.022-0.55 |  |
| [TBABr][AC] | Tetrabutylammonium bromide | | Acetic acid | 1:2 |  | 3.88-20.11 | 0.14-1.13 |  |
| [TEACl][AC] | Tetraethylammonium chloride | | Acetic acid | 1:2 |  | 2.81-20.18 | 0.14-1.18 |  |
| [TEACl][AC] | Tetraethylammonium chloride | | Acetic acid | 1:3 |  | 3.97-20.16 | 0.13-1.23 |  |
| [TEMACl][GLY] | Triethylmethylammonium chloride | | Glycerol | 1:2 |  | 1.5-16.48 | 0.017-0.43 |  |
| [TEMACl][LA] | Triethylmethylammonium chloride | | Lactic acid | 1:2 |  | 1.43-18.63 | 0.047-0.53 |  |
| [TEMACl][LEV] | Triethylmethylammonium chloride | | Levulinic acid | 1:2 |  | 1.36-16.17 | 0.057-0.61 |  |
| [TEMACl][EG] | Triethylmethylammonium chloride | | Ethylene glycol | 1:2 | 298.15 | 1.38-13.45 | 0.062-0.63 | (Sarmad et al., 2017) |
| [TEMACl][AC] | Triethylmethylammonium chloride | | Acetic acid | 1:2 |  | 1.98-18.37 | 0.081-1.18 |  |
| [ChCl][1,2-PRO] | Choline chloride | | 1,2-propanediol | 1:2 | 323.15 | 30 | 1.14 | (Mulia et al., 2017) |
| [N_8881_Br][DECA] | Methyltrioctylammonium bromide | | Decanoic acid | 1:2 | 298.15-323.15 | 0.9-19.9 | 0.041-1.31 | (Zubeir et al., 2018) |
| [N_8888_Cl][DECA] | Tetraoctylammonium chloride | |  |  |  |  | 0.042-1.41 |  |
| [N_8888_Br][DECA] | Tetraoctylammonium bromide | |  |  |  |  | 0.039-1.33 |  |
| [TMG][OA] | N,N,N-trimethylglycine | | Oxalic acid dihydrate | 1:2 | 298.15 | 40 | 0.005 | (Siani et al., 2020) |
| [TMG][GA] |  |  | Glycolic acid |  |  |  | 0.208 |  |
| [TMG][PA] |  |  | Phenylacetic acid |  |  |  | 0.330 |  |
| [ChCl][U] | Choline chloride | | Urea | 1:2.5 | 313.15 | 11.5 | 0.556 | (Li et al., 2008) |
| [ChCl][U] |  |  | Urea | 1:2 | 313.15 | 57.8 | 0.416 | (Leron et al., 2013) |
| [ChCl][EG] |  |  | Ethylene glycol | 1:2 | 313.15 | 61.6 | 0.250 | (Leron and Li, 2013a) |
| [ChCl][Gly] |  |  | Glycerol | 1:2 | 303.15 | 58.6 | 0.343 | (Leron and Li, 2013b) |
| [ChCl][LA] |  |  | Lactic acid | 1:1.5 | 303.15 | 35.9 | 1.062 | (Francisco et al., 2013) |
| [ChCl][TEG] |  |  | Triethylene glycol | 1:4 | 298.15 | 10 | 0.284 | (Ali et al., 2014) |
| [ChCl][U] |  |  | Urea | 1:4 |  |  | 0.316 |  |
| [ChCl][EG] |  |  | Ethylene glycol | 1:8 |  |  | 0.368 |  |
| [ChCl][Gly] |  |  | Glycerol | 1:3 |  |  | 0.433 |  |
| [ChCl][EA] |  |  | Ethanolamine | 1:6 |  |  | 1.51 |  |
| [ChCl][DEA] |  |  | Diethanolamine | 1:6 |  |  | 0.845 |  |
| [TBABr][EA] | Tetrabutylammonium bromide | | Ethanolamine | 1:6 |  |  | 1.779 |  |
| [TBABr][DEA] |  |  | Diethanolamine | 1:6 |  |  | 0.764 |  |
| [TBABr][TEA] |  |  | Triethanolamine | 1:3 |  |  | 0.431 |  |
| [BTPPCl][Gly] | Benzyltriphenylphosphonium chloride | | Glycerol | 1:12 |  |  | 0.444 |  |
| [BTPPBr][EA] | N-butyltriphenylphosphonium bromide | | Ethanolamine | 1:12 |  |  | 0.441 | (Ali et al., 2014) |
| [MTPPBr][EA] | Methytriphenylphosphonium bromide | | Ethanolamine | 1:6 |  |  | 1.393 |  |
| [TPPB][DEG] | Allyltriphenyl phosphonium bromide- | | Diethylene glycol | 1:4 | 303.15 | 19.46 | 7.29 |  |
|  |  |  |  | 1:10 |  | 19.54 | 5.79 | (Ghaedi et al., 2017) |
|  |  |  |  | 1:16 |  | 19.54 | 5.24 |  |
| [ATPPB][TEG] |  |  | Triethylene glycol | 1:4 |  | 19.50 | 6.42 |  |
|  |  |  |  | 1:10 |  | 19.53 | 4.77 |  |
|  |  |  |  | 1:16 |  | 19.57 | 4.29 |  |
| [TBAB][MDEA] | Tetrabutyl ammonium bromide | | Methyldiethanol amine | 1:4 | 303.15 | 10 | 1.81 | (Haider et al., 2018) |
|  | **Chemical-based DES** | | | | | |  |  |
| [HMIMCl][EDA] | Methylimidazolium chloride | | Ethylenediamine | 1:2 | 298.15 | 1 | 5.65 | (Shukla and Mikkola, 2018) |
| [HMIMCl][EDA] | Methylimidazolium chloride | | Ethylenediamine | 1:3 | 298.15 | 1 | 6.02 |  |
| [HMIMCl][EDA] | Methylimidazolium chloride | | Ethylenediamine | 1:4 | 298.15 | 1 | 6.96 |  |
| [HMIMCl][DETA] | Methylimidazolium chloride | | Diethylenetriamine | 1:4 | 298.15 | 1 | 5.18 |  |
| [MEACl][AP] | Monoethanolammonium chloride | | 3-amino-1-propanol | 1:3 | 298.15 | 1 | 5.20 |  |
| [MEACl][AP] | Monoethanolammonium chloride | | 3-amino-1-propanol | 1:4 | 298.15 | 1 | 5.78 |  |
| [MEACl][DETA] | Monoethanolammonium chloride | | Diethylenetriamine | 1:4 | 298.15 | 1 | 5.59 |  |
| [DBU][BA] | 2,3,4,6,7,8,9,10-octahydropyrimido [1,2-a]azepine | | Benzyl alcohol | 1:4 | 298.15 | 1 | 7.70-8.55 | (García-Argüelles et al., 2017) |
| [DBU][EG] | 2,3,4,6,7,8,9,10-octahydropyrimido [1,2-a]azepine | | Ethylene glycol | 1:4 | 298.15 | 1 | 12.23-12.48 |  |
| [TBD][BA] | 2,3,4,6,7,8-hexahydro-1h-pyrimido [1,2-a]pyrimidine | | Benzyl alcohol | 1:4 | 298.15 | 1 | 8.40-8.75 |  |
| [TBD][EG] | 2,3,4,6,7,8-hexahydro-1h-pyrimido [1,2-a]pyrimidine | | Ethylene glycol | 1:4 | 298.15 | 1 | 12.9 |  |
| [DBN][EU] | 1,5-diazabicyclo [4.3.0]non-5-ene | | 2-imidazolidone | 2:1 | 318.15 | 1 | 5.23 | (Jiang et al., 2019a) |
| [HMIMCl][AP] | Methylimidazolium chloride | | 3-amino-1-propanol | 1:4 | 298.15 | 1 | 4.42 | (Shukla and Mikkola, 2018) |
| [MEACl][AP] | Monoethanolammonium chloride | | 3-amino-1-propanol | 1:2 | 298.15 | 1 | 4.48 |  |
| [MEACl][TEPA] | Monoethanolammonium chloride | | Tetraethylenepentamine | 1:4 | 298.15 | 1 | 4.62 |  |
| [TBABr][AP] | Tetrabutylammonium bromide | | 3-amino-1-propanol | 1:4 | 298.15 | 1 | 4.09 |  |
| [TBD][BA] | 2,3,4,6,7,8-hexahydro-1h-pyrimido [1,2-a]pyrimidine | | Benzyl alcohol | 1:1 | 298.15 | 1 | 3.64-4.04 | (García-Argüelles et al., 2017) |
| [TBD][EG] | 2,3,4,6,7,8-hexahydro-1h-pyrimido [1,2-a]pyrimidine | | Ethylene glycol | 1:1 | 298.15 | 1 | 4.97 |  |
| [TBD][MDEA] | 2,3,4,6,7,8-hexahydro-1h-pyrimido [1,2-a]pyrimidine | | Methyldiethanolamine | 1:2 | 298.15 | 1 | 3.97-4.13 |  |
| [DBN][EU] | 1,5-diazabicyclo [4.3.0]non-5-ene | | 2-imidazolidone | 3:1 | 318.15 | 1 | 4.39 | (Jiang et al., 2019a) |
| [TETACl][DEG] | Triethylenetetramine chloride | | Diethylene glycol | 1:2 | 313.15 | 1 | 3.61 | (Zhang et al., 2018) |
| [TETACl][EG] | Triethylenetetramine chloride | | Ethylene glycol | 1:3 | 313.15 | 1 | 3.98 |  |
| [HMIMCl][AP] | Methylimidazolium chloride | | 3-amino-1-propanol | 1:1 | 298.15 | 1 | 0.41 | (Shukla and Mikkola, 2018) |
|  |  |  |  | 1:2 |  |  | 2.34 |  |
|  |  |  |  | 1:3 |  |  | 3.49 |  |
| [HMIMCl][EDA] | Methylimidazolium chloride | | Ethylenediamine | 1:1 | 298.15 | 1 | 2.13 |  |
| [HMIMCl][PEHA] | Methylimidazolium chloride | | Pentaethylenehexamine | 1:4 | 298.15 | 1 | 1.91 |  |
| [HMIMCl][TEPA] | Methylimidazolium chloride | | Tetraethylenepentamine | 1:4 | 298.15 | 1 | 2.23 |  |
| [MEACl][AP] | Monoethanolammonium chloride | | 3-amino-1-propanol | 1:1 | 298.15 | 1 | 3.24 |  |
| [MEACl][PEHA] | Monoethanolammonium chloride | | Pentaethylenehexamine | 1:4 | 298.15 | 1 | 2.87 |  |
| [TBABr][AP] | Tetrabutylammonium bromide | | 3-amino-1-propanol | 1:2 | 298.15 | 1 | 2.73 | (Shukla and Mikkola, 2018) |
| [TBABr][AP] | Tetrabutylammonium bromide | | 3-amino-1-propanol | 1:3 | 298.15 | 1 | 3.58 |  |
| [TBABr][AMP] | Tetrabutylammonium bromide | | Aminomethylpropanol | 1:3 | 298.15 | 1 | 2.37 |  |
| [TBABr][AMP] | Tetrabutylammonium bromide | | Aminomethylpropanol | 1:4 | 298.15 | 1 | 2.80 |  |
| [DBU][BA] | 2,3,4,6,7,8,9,10-octahydropyrimido [1,2-a]azepine | | Benzyl alcohol | 1:1 | 298.15 | 1 | 1.92-2.15 | (García-Argüelles et al., 2017) |
| ]DBU][EG] | 2,3,4,6,7,8,9,10-octahydropyrimido [1,2-a]azepine | | Ethylene glycol | 1:1 | 298.15 | 1 | 2.80-3.08 |  |
| [DBU][MDEA] | 2,3,4,6,7,8,9,10-octahydropyrimido [1,2-a]azepine | | Methyldiethanolamine | 1:2 | 298.15 | 1 | 3.84 |  |
| [DBN][DMU] | 1,5-diazabicyclo [4.3.0]non-5-ene | | Dimethylolurea | 2:1 | 318.15 | 1 | 0.97 | (Jiang et al., 2019a) |
| [DBN][DMLU] | 1,5-diazabicyclo [4.3.0]non-5-ene | | 1,3-dimethylurea |  | 318.15 | 1 | 3.94 |  |
| [MEACl][EDA] | Monoethanolamine chloride | | Ethylenediamine | 1:4 | 303.15 | 1 | 7 | (Trivedi et al., 2016) |
| [MEACl][EDA] | Monoethanolamine chloride | | Ethylenediamine | 1:3 | 303.15 | 1 | 7.66 |  |
| [TEACl][EDA] | Triethanolamine chloride | | Ethylenediamine | 1:3 | 303.15 | 1 | 3.869 |  |
| [UECl][EDA] | Urea chloride | | Ethylenediamine | 1:3 |  |  | 2.659 | (Trivedi et al., 2016) |
| [TAECl][EDA] | Triethanolamine chloride | | Ethylenediamine | 1:3 | 303.15 | 1 | 2.302 |  |
| [ChCl][EA] | Choline chloride | | Ethanolamine | 1:7 | 298.15 | 20.15 | 5.55 | (Sarmad et al., 2020) |
| [ChCl][EA][DEA] |  |  | Ethanolamine/Diethanolamine | 1:7:1 |  | 20.11 | 6.08 |  |
| [ChCl][EA][MDE] |  |  | Ethanolamine/Methyldiethanolamine | 1:7:1 |  | 20.09 | 4.59 |  |
| [ChCl][EA][MDE] | Choline chloride | | Ethanolamine/ Methyldiethanolamine | 1:7:5 | 298.15 | 20.11 | 3.72 | (Sarmad et al., 2020) |
| [ChCl][EA][AEP] |  |  | Ethanolamine/Amino ethyl piperazine | 1:7:1 |  | 20.05 | 4.76 |  |
| [ChCl][EA][Pz] |  |  | Ethanolamine/Piperazine | 1:7:1 |  | 22.37 | 7.76 |  |
| [DBN][BMIMCl][Im] | 1,5-diazabicyclo [4.3.0] non-5-ene | | 1-butyl-3-methylimidazolium chloride/imidazole | 1:1:1 | 298.15 | 1 | 8.34 | (Zhang et al., 2019a) |
| [DBN][BMIMCl][Im] |  |  |  | 1:1:2 |  |  | 8.92 |  |
| [DBN][BMIMCl][Im] |  |  |  | 1:2:1 |  |  | 7.90 |  |
| [TBAB][MEA] | Tetrabutyl ammonium bromide | | 2-methylaminoethanol | 1:4 | 303.15 | 10.46 | 2.41 | (Haider and Kumar, 2020) |
| [BTEACl][MEA] | Benzyltriethyl ammonium chloride | | 2-methylaminoethanol | 1:4 |  | 10.07 | 2.27 |  |
| [TBAB][EAE] | Tetrabutyl ammonium bromide | | 2-ethylaminoethanol | 1:4 |  | 9.93 | 1.61 |  |
| [BTEACl][EAE] | Benzyltriethyl ammonium chloride | | 2-ethylaminoethanol | 1:4 |  | 10.19 | 2.05 |  |
| [P_2222_][Triz][EG] | Tetraethylphosphonium 1,2,4- triazolate | | Ethylene glycol | 1:2 | 298.15 | 1 | 2.68 | (Cui et al., 2019) |
| [P_2222_][Im][EG] | Tetraethylphosphonium imidazolate | | Ethylene glycol | 1:2 | 298.15 | 1 | 2.68 | (Cui et al., 2019) |
| [N_2222_][Triz][EG] | Tetraethylammonium 1,2,4- triazolate | | Ethylene glycol | 1:2 | 298.15 | 1 | 2.84 | (Cui et al., 2019) |
| [N_2222_][Im][EG] | Tetraethylammonium imidazolate | | Ethylene glycol | 1:2 | 298.15 | 1 | 2.93 | (Cui et al., 2019) |
| [MEA][Im][EG] | Monoethanolamine imidazolate | | Ethylene glycol | 1:1:1 | 298.15 | 1 | 3.26 | (Mukesh et al., 2019) |
| [DETA]_2_[Im][EG] | Diethylenetriamine imidazolate | | Ethylene glycol | 1:2:2 | 298.15 | 1 | 5.08 | (Mukesh et al., 2019) |
| [TEPA]_2_[Im][EG] | Tetraethylenepentamine imidazolate | | Ethylene glycol | 1:2:4 | 298.15 | 1 | 3.94 | (Mukesh et al., 2019) |
| [TETACl][thymol] | Triethylenetetramine chloride | | Thymol | 1:3 | 313.15 | 1.013 | 2.045 | (Gu et al., 2020) |
| [TEPACl][thymol] | Tetraethylpentamine chloride | | Thymol | 1:3 | 313.15 | 1.013 | 2 | (Gu et al., 2020) |
| [HDBU][Im][EG] | 1,8-diazabicyclo- [5,4,0]undec-7-ene imidazole | | Ethylene glycol | 7:3 | 313 | 1 | 3.20 | (Yan et al., 2020) |
| [HDBU][Ind][EG] | 1,8- diazabicyclo[5,4,0]undec-7-ene indole | | Ethylene glycol | 7:3 | 313 | 1 | 2.66 | (Yan et al., 2020) |
| [HDBU][Triz][EG] | 1,8-diazabicyclo[5,4,0]undec-7-ene 1,2,4-triazole | | Ethylene glycol | 7:3 | 313 | 1 | 2.45 | (Yan et al., 2020) |
| [HDBU][Im][EG] | 1,8-diazabicyclo- [5,4,0]undec-7-ene imidazole | | Ethylene glycol | 6:4 | 313 | 1 | 2.68 | (Yan et al., 2020) |
| [HDBU][Im][EG] | 1,8-diazabicyclo- [5,4,0]undec-7-ene imidazole | | Ethylene glycol | 5:5 | 313 | 1 | 2.48 | (Yan et al., 2020) |
| [HDBU][Im][EG] | 1,8-diazabicyclo- [5,4,0]undec-7-ene imidazole | | Ethylene glycol | 4:6 | 313 | 1 | 1.86 | (Yan et al., 2020) |
| [HDBU][Im][EG] | 1,8-diazabicyclo- [5,4,0]undec-7-ene imidazole | | Ethylene glycol | 3:7 | 313 | 1 | 1.43 | (Yan et al., 2020) |
| [Et_4_N][Thy][EG] | Tetraethylammonium thymol | | Ethylene glycol | 1:2 | 298.15 | 1 | 5.23 | (Wang et al., 2021b) |
| [Et_4_N][Car][EG] | Tetraethylammonium carvacrol | | Ethylene glycol | 1:2 | 298.15 | 1 | 5.06 | (Wang et al., 2021b) |
| [Et_4_N][Thy][4CH_3_-Im] | Tetraethylammonium thymol | | 4-methylimidazole | 1:2 | 298.15 | 1 | 4.86 | (Wang et al., 2021b) |
| [Et_4_N][Car][4CH_3_-Im] | Tetraethylammonium carvacrol | | 4-methylimidazole | 1:2 | 298.15 | 1 | 4.75 | (Wang et al., 2021b) |
| [DBUH][MLU][EG] | 1,8-diazabicyclo[5.4.0]undec-7-ene methyl urea | | Ethylene glycol | 1:1 | 313.15 | 1 | 3.14 | (Fu et al., 2021b) |
| [DBUH][MLU][EG] | 1,8-diazabicyclo[5.4.0]undec-7-ene methyl urea | | Ethylene glycol | 1:2 | 313.15 | 1 | 2.70 | (Fu et al., 2021b) |
| [DBUH][MLU][EG] | 1,8-diazabicyclo[5.4.0]undec-7-ene methyl urea | | Ethylene glycol | 1:4 | 313.15 | 1 | 2 | (Fu et al., 2021b) |
| [DBUH]_2_[DMU][EG] | 1,8-diazabicyclo[5.4.0]undec-7-ene 1,3-dimethylurea | | Ethylene glycol | 1:1 | 313.15 | 1 | 2.95 | (Fu et al., 2021b) |
| [DBUH]_2_[DMU][EG] | 1,8-diazabicyclo[5.4.0]undec-7-ene 1,3-dimethylurea | | Ethylene glycol | 1:2 | 313.15 | 1 | 3.70 | (Fu et al., 2021b) |
| [DBUH]_2_[DMU][EG] | 1,8-diazabicyclo[5.4.0]undec-7-ene 1,3-dimethylurea | | Ethylene glycol | 1:4 | 313.15 | 1 | 3.05 | (Fu et al., 2021b) |
| [EMIM][2CNpyr][EG] | 1-ethyl-3-methylimidazolium 2- cyanopyrrolide | | Ethylene glycol | 1:1 | 298.15 | 1 | ~3.5 | (Lee et al., 2021) |
| [EMIM][2CNpyr][EG] | 1-ethyl-3-methylimidazolium 2- cyanopyrrolide | | Ethylene glycol | 1:2 | 298.15 | 1 | 2.59 | (Lee et al., 2021) |
| [DBUH][Car][EG] | 1,8-diazabicyclo- [5,4,0]undec-7-ene carvacrol | | Ethylene glycol | 1:4 | 298.15 | 1 | 8.99 | (Wang et al., 2022) |
| [DBUH][Car][EG] | 1,8-diazabicyclo- [5,4,0]undec-7-ene carvacrol | | Ethylene glycol | 1:3 | 298.15 | 1 | 8.10 | (Wang et al., 2022) |
| [DBUH][Car][EG] | 1,8-diazabicyclo- [5,4,0]undec-7-ene carvacrol | | Ethylene glycol | 1:2 | 298.15 | 1 | 6.82 | (Wang et al., 2022) |
| [DBUH][Thy][EG] | 1,8-diazabicyclo- [5,4,0]undec-7-ene thymol | | Ethylene glycol | 1:4 | 298.15 | 1 | 9.08 | (Wang et al., 2022) |
| [DBUH][Thy][EG] | 1,8-diazabicyclo- [5,4,0]undec-7-ene thymol | | Ethylene glycol | 1:3 | 298.15 | 1 | 8.10 | (Wang et al., 2022) |
| [DBUH][Thy][EG] | 1,8-diazabicyclo- [5,4,0]undec-7-ene thymol | | Ethylene glycol | 1:2 | 298.15 | 1 | 6.82 | (Wang et al., 2022) |
| [Gly]:[MEA]/EG | Glycine:monoethanolamine | | Ethylene glycol | 1:4/30 wt.% | 303.15 | 1.01 | ~5 | (Qian et al., 2022) |
| [Pro]:[MEA]/EG | Proline: monoethanolamine | | Ethylene glycol | 1:4/30 wt.% | 303.15 | 1.01 | ~4.09 | (Qian et al., 2022) |

**Table S3.** Top 20 physical-based ILs.

| Abbreviation | Full name | Structure | | Mw (g/mol) | *T* (K) | *P* (bar) | Viscosity  (cP) | Selectivity | Absorption capacity  (mol/kg) | Ref. |
| --- | --- | --- | --- | --- | --- | --- | --- | --- | --- | --- |
|  |  | **Cation** | **Anion** |  |  |  |  |  |  |  |
| [BVIM][Tf_2_N] | 1- butyl-3-vinylimidazolium bis(trifluoromethylsulfonyl)imide | 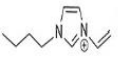 | 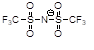 | 431.4 | 303.2 | 71.1 | 77 at 298.15 K | - | 6.268 | (Yim et al., 2021) |
| [P_4441_][Tf_2_N] | Tributylmethylphosphonium bis(trifluoromethylsulfonyl)imide | 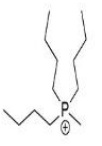 | 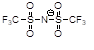 | 497.49 | 303.2 | 103.9 | 209 at 298.15 K | - | 6.063 | (Yim et al., 2021) |
| [HMIM][FAP] | 1-hexyl-3-methylimidazo tris (pentafluoroethyl) trifluorophosphate | 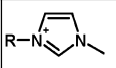 | 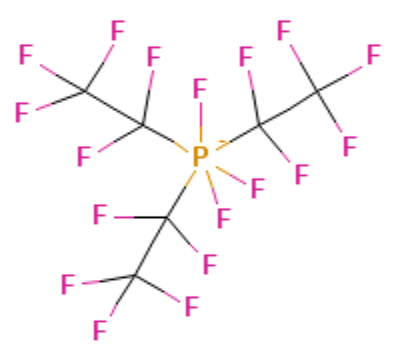 | 612.29 | 293.15 | 50 | 116 at 293 | - | ~5.791 | (Wu et al., 2021) |
| [BMIM][FAP] | 1-butyl-3-methylimidazo tris (pentafluoroethyl) trifluorophosphate | 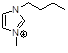 | 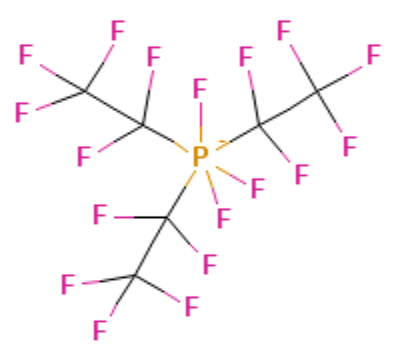 | 584.23 | 293.15 | 50 | 93 at 293 | - | ~4.628 | (Wu et al., 2021) |
| [BZMIM][Tf_2_N] | 1-benzyl-3-methylimidazolium bis(trifluoromethylsulfonyl) imide | 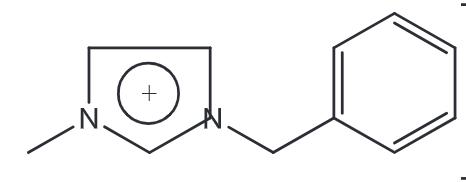 | 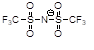 | - | 303.15 | 30.496 | - | 2.89 (CO_2_/H_2_S) | 4.23 | (Jalili et al., 2022) |
| [BMIM][BF_4_] | 1-butyl-3-methylimidazolium tetrafluoroborate | 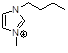 | 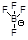 | 226.02 | 273.15-353.15 | 5.28-34.38 | - | - | 2.49-4.2 | (Jiang et al., 2019b) |
| [EMIM][FAP] | 1-ethyl-3-methylimidazo tris (pentafluoroethyl) trifluorophosphate | 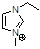 | 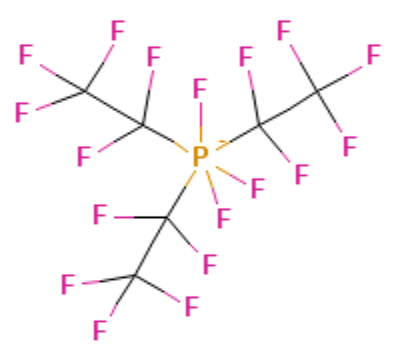 | 556.17 | 293.15 | 50 | 75 at 293 | - | ~ 4.195 | (Wu et al., 2021) |
| [N_4222_][Tf_2_N] | Butyltriethylammonium bis(trifluoromethylsulfonyl)imide | 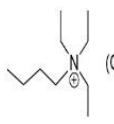 | 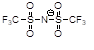 | 438.45 |  | 124.2 | 99 at 298.15 K | - | 4.180 | (Yim et al., 2021) |
| [AMIM][Tf_2_N] | 1-allyl-3-methylimidazolium bis(trifluoromethyl sulfonyl)imide | 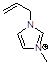 | 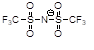 | 404.33 | 313.2-353.2 | 9.5-58.3 | - | - | 0.27-3.88 | (Taheri et al., 2018) |
| [DEA][Bu] | Diethylammonium butanoate | 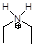 | 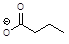 | 161.29 | 303-333 | 7.25-196.70 | - | Ideal:  12 (CO_2_/CH_4_, 303.1)  21 (CO_2_/N_2_, 303.1) | 0.63-3.71 | (Alcantara et al., 2018) |
| [C_12_MIM][PF_6_] | 1-dodecyl-3-methylimidazolium hexafluorophosphate | 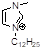 | 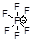 | - | 303.15 | 40 | - | 5.53 (CO_2_/CH_4_) | 3.643 | (Wang et al., 2020) |
| [EMIM][CH_3_SO_3_] | 1-ethyl-3-methylimidazolium methanesulfonate | 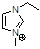 | 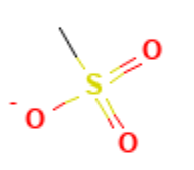 | - | 293.15 | 45 | - | - | 3.237 | (Safarov et al., 2021) |
| [EMIM][Tf_2_N] | 1-ethyl-3-methylimidazolium bis(trifluoromethylsulfonyl)imide | 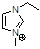 | 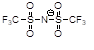 | 391.31 | 298.15 | 40 | - | - | ~2.746 | (Peng et al., 2021) |
| [C_6_MIM][Tf_2_N] | 1-hexyl-3-methyl-imidazolium bis(trifluoromethylsulfonyl) amide | 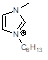 | 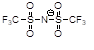 | 447.42 | 303.15-353.15 | 0-35.555 | - | - | 0-2.65 | (Jalili et al., 2017) |
| [C_12_MIM][BF_4_] | 1-dodecyl-3-methyl-imidazolium tetrafluoroborate | 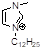 | 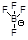 | 338.24 | 333.15-373.15 | 9.40-57.30 | - | ~15 (CO/CO_2_, 298.15)  ~17 (H_2_/CO_2_, 298.15) | 0.29-2.5 | (Dai et al., 2017) |
| [OMIM][Tf_2_N] | 1-methyl-3-octylimidazolium bis(trifluoromethylsulfonyl)imide | 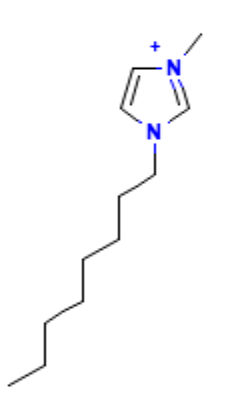 | 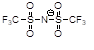 | 475.47 | 298.15 | 40 | - | - | 2.499 | (Peng et al., 2021) |
| [HMIM][Tf_2_N] | 1-hexyl-3-methylimidazolium bis(trifluoromethylsulfonyl)imide | 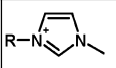 | 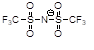 | 447.42 | 298.15 | 40 | - | - | ~2.421 | (Peng et al., 2021) |
| [BMIM][Tf_2_N] | 1-butyl-3-methylimidazolium bis(trifluoromethylsulfonyl)imide | 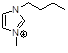 | 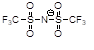 | 419.36 | 298.15 | 35 | - | - | ~2.201 | (Peng et al., 2021) |
| [C_16_MIM][PF_6_] | 1-hexadecyl-3-methylimidazolium hexafluorophosphate | 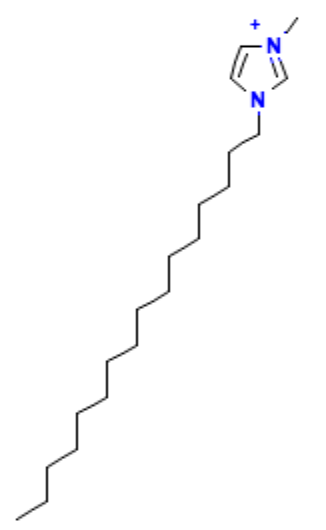 | 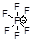 | - | 303.15 | 40 | - | 4.21 (CO_2_/CH_4_) | 2.08 | (Wang et al., 2020) |
| [BMIM][ATZ] | 1-butyl-3-methylimidazolium 3-amino-1H-1,2,4-triazolate | 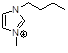 | 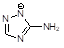 | 222.29 | 298.15 | 1 | - | - | 2.04 ± 0.06 | (Zhang et al., 2019b) |

**Table S4.** Top 20 chemical-based ILs.

| Abrreviation | Full name | Structure | | Mw (g/mol) | *T* (K) | *P* (bar) | Absorption capacity  (mol/kg) | Viscosity  (cP) | Ref. |
| --- | --- | --- | --- | --- | --- | --- | --- | --- | --- |
|  |  | **Cation** | **Anion** |  |  |  |  |  |  |
| [DETAH][Im] | Diethylenetriamine imidazolium | 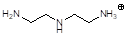 | 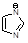 | 171.25 | 313.15 | 1 | 11.91 | 0.797 | (Wu et al., 2019) |
| [DETAH][Py] | Diethylenetriamine pyrazolium | 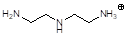 | 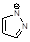 | 171.25 | 313.15 | 1 | 11.39 | 0.772 | (Wu et al., 2019) |
| [DETAH][Gly] | Diethylenetriamine glycinate | 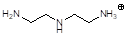 | 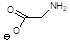 | 178.24 | 313.15 | 1 | 10.15 | - | (Wu et al., 2019) |
| [DETAH][Tz] | Diethylenetriamine 1,2,4-triazolate | 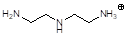 | 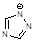 | 172.23 | 313.15 | 1 | 10.10 | 0.822 | (Wu et al., 2019) |
| [TETAH][Lys] | Triethylenetetramine lysine | 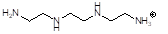 | 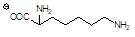 | 266.43 | 313.15 | 1 | 9.72 | 1.15 | (Jing et al., 2018) |
| [MOBMIM][Gly] | 1-methoxylbutyl-3-  methylimidazolium glycine | 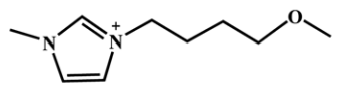 | 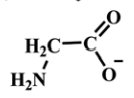 | - | 323.15 | 5 | 9.66 | 536.31 | (Qu et al., 2021) |
| [EMIM][Gly] | 1-ethyl-3- methylimidazolium glycinate | 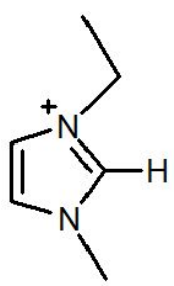 | 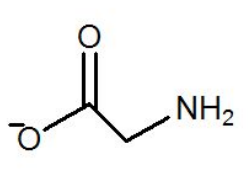 | - | 298.15 | 40 (ml/min) | 8.645 | - | (Min et al., 2021) |
| [DETAH][Lys] | Diethylenetriamine lysine | 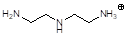 | 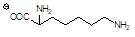 | 277.39 | 313.15 | 1 | 7.68 | 1.05 | (Jing et al., 2018) |
| [BMIM][Gly] | 1-butyl-3-methylimidazolium glycine | 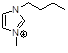 | 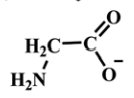 | - | 323.15 | 5 | 7.251 | 767.32 | Qu et al., 2021) |
| [MOBMIM][His] | 1-methoxylbutyl-3-  methylimidazolium histidine | 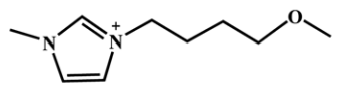 | 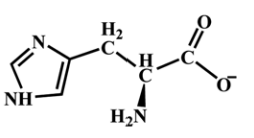 | - | 323.15 | 5 | 7.026 | 2986.94 | (Qu et al., 2021) |
| [MOBMIM][Lys] | 1-methoxylbutyl-3-methylimidazolium lysine | 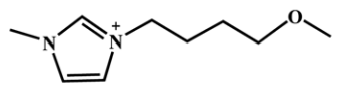 | 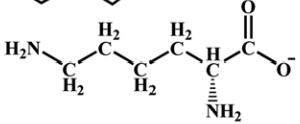 | - | 323.15 | 5 | 6.721 | 3201.13 | (Qu et al., 2021) |
| [Cho][Gly] | Cholinium glycinate | 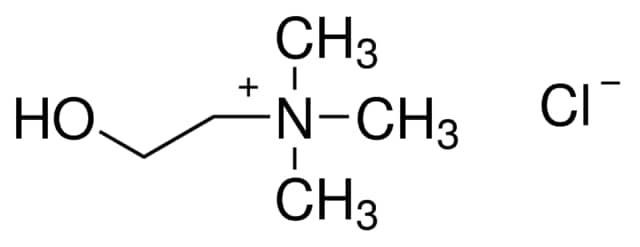 | 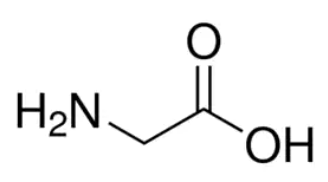 | - | 298.15 | 4 | 6.20 | - | (Noorani and Mehrdad, 2021) |
| [MOBMIM][Arg] | 1-methoxylbutyl-3-methylimidazolium arginine | 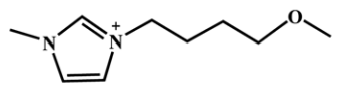 | 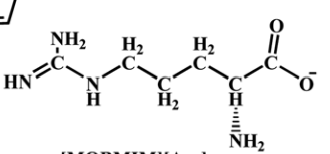 | - | 323.15 | 5 | 6.086 | 5631.12 | (Qu et al., 2021) |
| [P_4442_][Cy-Suc] | Tributyl(ethyl)phosphonium 1,2-cyclohexanedicarboximide | 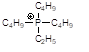 | 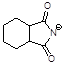 | 383.56 | 293.15 | 1 | 5.76 | - | (Huang et al., 2018) |
| [DMEDAH] [Py] | N,N-dimethylethylenediamine pyrazole | 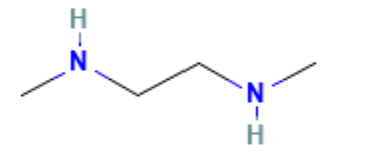 | 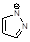 | - | 295.15 | 1.01 | 5.25 | - | (Wang et al., 2021a) |
| [P_4442_][Suc] | Tributyl(ethyl)phosphonium succinimido | 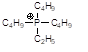 | 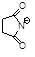 | 329.46 | 293.15, 303.15 | 0.1 | 5,  3.4 | - | (Huang et al., 2017) |
| [BMPyr][2-Op] | - | 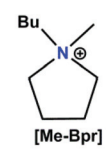 | 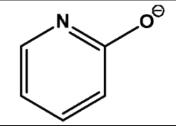 | 236.36 | 303.15 | 1 | 4.95 | - | (Luo et al., 2019a) |
| [DMEDAH] [Im] | N,N-dimethylethylenediamine imidazole | 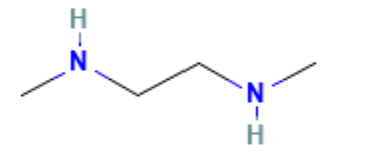 | 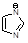 | - | 295.15 | 1.01 | 4.91 | - | (Wang et al., 2021a) |
| [Ph-C_8_eim][2-Op] | 1n-ethyl-3n-octyl-2-phenylimidazolium 2-hydroxylpyridium | 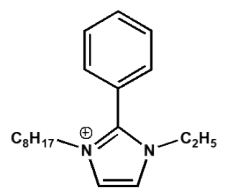 | 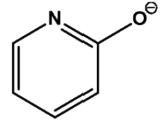 | 379.54 | 293.15 | 1 | 4.45 | 1112 at 298 K | (Luo et al., 2019a) |
| [P_4442_][2-Op] | Tributyl(ethyl)phosphonium 2-hydroxylpyridium | 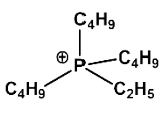 | 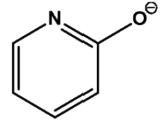 | 325.47 | 303.15 | 1 | 4.30 | 193.0 at 298 K | (Luo et al., 2019a) |

**Table S5.** Top 20 physical-based DESs.

| Abbreviation | Molar ratio (HBA:HBD) | *T* (K) | *P* (bar) | Absorption capacity (mol/kg) | Ref. |
| --- | --- | --- | --- | --- | --- |
| [EAHCl][TEPA] | 1:9 | 303.15 | 16.03 | 9.671 | (Pishro et al., 2021) |
| [TPPB][DEG] | 1:4 | 303.15 | 19.46 | 7.29 | (Ghaedi et al., 2017) |
| [ATPPB][TEG] | 1:4 | 303.15 | 19.50 | 6.42 | (Ghaedi et al., 2017) |
| [TPPB][DEG] | 1:10 | 303.15 | 19.54 | 5.79 | (Ghaedi et al., 2017) |
| [TPPB][DEG] | 1:16 | 303.15 | 19.54 | 5.24 | (Ghaedi et al., 2017) |
| [L-Arg][GLY] | 1:6 | 333.15 | 1 | 4.92 | (Ren et al., 2018) |
| [ATPPB][TEG] | 1:10 | 303.15 | 19.53 | 4.77 | (Ghaedi et al., 2017) |
| [ChCl][La] | 1:1 | 298.15 | 50 | 4.52 | (Altamash et al., 2020) |
| [Al][La] | 1:1 | 298.15 | 50 | 4.30 | (Altamash et al., 2020) |
| [ATPPB][TEG] | 1:16 | 303.15 | 19.57 | 4.29 | (Ghaedi et al., 2017) |
| [Be][La] | 1:1 | 298.15 | 50 | 4.26 | (Altamash et al., 2020) |
| [ChCl][Fr] | 1:1 | 298.15 | 50 | 4.24 | (Altamash et al., 2020) |
| [ChCl][Ma] | 1:1 | 298.15 | 50 | 4.22 | (Altamash et al., 2020) |
| [Al][Ma] | 1:1 | 298.15 | 50 | 4.14 | (Altamash et al., 2020) |
| [ChCl][MEA] | 1:5 | 313.15 | 12.5 | ~3.775 | (Yan et al., 2022) |
| [ChCl][EA] | 1:7 | 298.15 | 1.82-20.35 | 0.78-3.58 | (Sarmad et al., 2017) |
| [TPACl][EA] | 1:7 | 298.15 | 3.57-20.19 | 1.71-3.53 | (Sarmad et al., 2017) |
| [ChCl][EG] | 1:2 | 303.15 | 58.63 | 3.216 | (Leron and Li, 2013b;a) |
| [MTPPBr][AC] | 1:4 | 298.15 | 1.73-20.14 | 0.073-3.02 | (Sarmad et al., 2017) |
| [TBABr][EA] | 1:7 | 298.15 | 3.81-20.40 | 0.53-3.01 | (Sarmad et al., 2017) |

**Table S6.** Top 20 and (5 additional) chemical-based DESs*.

| Abberviation | Molar ratio (HBA:HBD) | *T* (K) | *P* (bar) | | Absorption capacity (mol/kg) | | Ref. |
| --- | --- | --- | --- | --- | --- | --- | --- |
| [TBD][EG] | 1:4 | 298.15 | 1 | 12.9 | | (García-Argüelles et al., 2017) | |
| [DBU][EG] | 1:4 | 298.15 | 1 | 12.23-12.48 | | (García-Argüelles et al., 2017) | |
| [ChCl][U]/AMP | 1:2 | 298.15 | 2 | 11 | | (Uma Maheswari and Palanivelu, 2015) | |
| [ChCl][TEG]/AMP | 1:4 | 298.15 | 2 | 10.51 | | (Uma Maheswari and Palanivelu, 2015) | |
| [ChCl][DEG]/AMP | 1:4 | 298.15 | 2 | 10.36 | | (Uma Maheswari and Palanivelu, 2015) | |
| [ChCl][U]/MAE | 1:2 | 298.15 | 2 | 9.97 | | (Uma Maheswari and Palanivelu, 2015) | |
| [ChCl][U]/MEA | 1:2 | 298.15 | 2 | 9.84 | | (Uma Maheswari and Palanivelu, 2015) | |
| [DBUH][Thy]/EG | 1:4 | 298.15 | 1 | 9.08 | | (Wang et al., 2022) | |
| [DBUH][Car]/EG | 1:4 | 298.15 | 1 | 8.99 | | (Wang et al., 2022) | |
| [DBN][BMIMCl][Im] | 1:1:2 | 298.15 | 1 | 8.92 | | (Zhang et al., 2019a) | |
| [TBD][BA] | 1:4 | 298.15 | 1 | 8.40-8.75 | | (García-Argüelles et al., 2017) | |
| [DBU][BA] | 1:4 | 298.15 | 1 | 7.70-8.55 | | (García-Argüelles et al., 2017) | |
| [DBN][BMIMCl][Im] | 1:1:1 | 298.15 | 1 | 8.34 | | (Zhang et al., 2019a) | |
| [DBU][Pyr] | 2:1 | 303 | 1 | 8.193 | | (Fu et al., 2021a) | |
| [DBUH][Car]/EG | 1:3 | 298.15 | 1 | 8.10 | | (Wang et al., 2022) | |
| [DBUH][Thy]/EG | 1:3 | 298.15 | 1 | 8.10 | | (Wang et al., 2022) | |
| [DBN][BMIMCl][Im] | 1:2:1 | 298.15 | 1 | 7.90 | | (Zhang et al., 2019a) | |
| [ChCl][EA][Pz] | 1:7:1 | 298.15 | 22.37 | 7.76 | | (Sarmad et al., 2020) | |
| [MEACl][EDA] | 1:3 | 303.15 | 1 | 7.66 | | (Trivedi et al., 2016) | |
| [DBU][Eth] | 2:1 | 303 | 1 | 7.504 | | (Fu et al., 2021a) | |
| [HMIMCl][EDA] | 1:4 | 298.15 | 1 | 6.96 | | (Shukla and Mikkola, 2018) | |
| [DBU][Py] | 1:1 | 303 | 1 | 6.5 | | (Fu et al., 2021a) | |
| [DBU][Oxa] | 2:1 | 303 | 1 | 6.336 | | (Fu et al., 2021a) | |
| [HMIMCl][EDA] | 1:3 | 298.15 | 1 | 6.02 | | (Shukla and Mikkola, 2018) | |
| [MEACl][AP] | 1:4 | 298.15 | 1 | 5.78 | | (Shukla and Mikkola, 2018) | |

*****As the molar ratios of 5 DESs were not mentioned in the literature, it is impossible to predict their properties with COSMO-RS, and other 20 DESs were provided for further screening.

**Table S7.** The selectivity of CO_2_ over other gases and viscosity of ILs as well as the excess enthalpy of equimolar CO_2_ in physical- and chemical-based ILs predicted by the COSMO-RS model at 298.15 K and 1 bar.

| Abbreviation | Selectivity | | | | Viscosity (cP) | Excess enthalpy of equimolar CO_2_/(J/mol) |
| --- | --- | --- | --- | --- | --- | --- |
|  | **CO_2_/CO** | **CO_2_/H_2_** | **CO_2_/CH_4_** | **CO_2_/N_2_** |  |  |
| Physical-based ILs | | | | | | |
| [AMIM][Tf_2_N] | 50.09 | 48.71 | 20.28 | 12.36 | 44.89 | -289.23 |
| [BMIM][BF_4_] | 58.73 | 47.72 | 22.81 | 24.87 | 164.40 | -347.98 |
| [DEA][Bu] | 40.02 | 26.92 | 12.56 | 16.80 | 378.36 | -468.94 |
| [C_6_MIM][Tf_2_N] | 42.80 | 43.75 | 15.13 | 12.56 | 76.47 | -425.19 |
| [C_12_MIM][BF_4_] | 43.01 | 40.46 | 14.29 | 20.95 | 777.04 | -253.90 |
| [BMIM][ATZ] | 45.76 | 49.54 | 17.64 | 24.63 | 222.24 | -767.99 |
| [EMIM][FAP] | 40.05 | 52.25 | 16.03 | 14.10 | 25.83 | -673.78 |
| [BMIM][FAP] | 37.21 | 48.43 | 14.17 | 13.78 | 37.78 | -670.90 |
| [HMIM][FAP] | 35.11 | 45.47 | 12.86 | 13.50 | 54.18 | -662.10 |
| [C_12_MIM][PF_6_] | 42.63 | 44.08 | 14.74 | 19.81 | 983.97 | -415.23 |
| [C_16_MIM][PF_6_] | 33.47 | 36.50 | 10.41 | 12.24 | 1556.70 | -453.04 |
| [EMIM][Tf_2_N] | 50.90 | 48.42 | 20.12 | 12.56 | 33.59 | -309.73 |
| [BMIM][Tf_2_N] | 45.94 | 46.30 | 17.05 | 12.55 | 51.44 | -390.33 |
| [HMIM][Tf_2_N] | 42.46 | 44.17 | 15.04 | 12.48 | 76.47 | -425.19 |
| [OMIM][Tf_2_N] | 39.97 | 42.30 | 13.68 | 12.44 | 106.91 | -437.74 |
| [EMIM][CH_3_SO_3_] | 43.63 | 49.28 | 15.91 | 11.78 | 122.61 | -998.75 |
| [BVIM][Tf_2_N] | 51.19 | 46.38 | 16.24 | 14.18 | 55.69 | -384.23 |
| [N_4222_][Tf_2_N] | 39.08 | 45.56 | 18.98 | 12.97 | 53.96 | -360.13 |
| [P_4441_][Tf_2_N] | 46.17 | 41.13 | 12.69 | 11.88 | 106.20 | -601.32 |
| [BZMIM][Tf_2_N] | 45.43 | 50.78 | 18.53 | 19.82 | 76.33 | -405.74 |
| Chemical-based ILs | | | | | | |
| [DETAH][Lys] | 14.10 | 62.45 | 13.52 | 26.13 | 11579.08 | -285.18 |
| [TETAH][Lys] | 15.22 | 59.76 | 12.81 | 25.13 | 27389.91 | -316.27 |
| [DETAH][Tz] | 15.53 | 79.28 | 21.49 | 31.14 | 1014.26 | -337.75 |
| [DETAH][Gly] | 11.65 | 63.54 | 14.63 | 26.19 | 2317.28 | -277.08 |
| [DETAH][Py] | 14.53 | 72.83 | 18.37 | 26.96 | 1600.02 | -265.74 |
| [DETAH][Im] | 15.33 | 79.28 | 20.63 | 29.73 | 1008.85 | -220.54 |
| [P_4442_][Suc] | 29.03 | 76.11 | 14.49 | 20.08 | 494.77 | -663.46 |
| [P_4442_][Cy-Suc] | 26.46 | 70.81 | 13.15 | 19.02 | 791.60 | -542.68 |
| [EMIM][Gly] | 30.11 | 64.26 | 14.00 | 16.43 | 154.96 | -1001.64 |
| [MOBMIM][Lys] | 26.07 | 70.76 | 14.44 | 20.42 | 3962.51 | -510.70 |
| [MOBMIM][His] | 27.81 | 73.79 | 16.75 | 20.24 | 369.45 | -675.84 |
| [MOBMIM][Arg] | 27.15 | 72.85 | 16.09 | 22.41 | 3643.64 | -669.69 |
| [MOBMIM][Gly] | 28.01 | 69.67 | 14.45 | 18.02 | 525.96 | -841.21 |
| [BMIM][Gly] | 67.77 | 71.44 | 23.02 | 29.60 | 174.97 | -655.41 |
| [BMPyr][2-Op] | 30.35 | 81.01 | 17.22 | 22.25 | 257.26 | -740.28 |
| [Ph-C_8_eim][2-Op] | 28.48 | 62.70 | 13.28 | 15.81 | 586.36 | -549.08 |
| [P_4442_][2-Op] | 29.14 | 71.37 | 14.34 | 19.82 | 454.80 | -645.01 |
| [Cho][Gly] | 40.57 | 41.33 | 14.99 | 19.94 | 220.79 | -1296.13 |
| [DMEDAH] [Py] | 45.51 | 20.18 | 14.91 | 22.49 | 750.15 | -251.10 |
| [DMEDAH] [Im] | 45.51 | 21.91 | 17.38 | 22.49 | 464.38 | -231.19 |

**Table S8**. The selectivity of CO_2_ over other gases and excess enthalpy of equimolar CO_2_ in physical- and chemical-based DESs predicted by the COSMO-RS model at 298.15 K and 1 bar.

| Abbreviation | Selectivity | | | | | Excess enthalpy of equimolar CO_2_/(J/mol) |
| --- | --- | --- | --- | --- | --- | --- |
|  | **Molar ratio (HBA:HBD)** | **CO_2_/CO** | **CO_2_/H_2_** | **CO_2_/CH_4_** | **CO_2_/N_2_** |  |
| Physical-based DES | | | | | | |
| [EAHCl][TEPA] | 1:9 | 30.38 | 44.09 | 11.08 | 26.19 | -877.27 |
| [TPPB][DEG] | 1:4 | 34.11 | 36.51 | 10.82 | 11.79 | -256.25 |
| [ATPPB][TEG] | 1:4 | 30.15 | 29.03 | 8.95 | 9.97 | -133.78 |
| [TPPB][DEG] | 1:10 | 34.85 | 32.13 | 10.30 | 10.72 | -93.06 |
| [TPPB][DEG] | 1:16 | 35.15 | 30.62 | 10.11 | 10.35 | -53.60 |
| [L-Arg][GLY] | 1:6 | 32.03 | 20.88 | 8.65 | 8.58 | -130.83 |
| [ATPPB][TEG] | 1:10 | 29.97 | 25.02 | 8.32 | 8.96 | -26.28 |
| [ChCl][La] | 1:1 | 32.55 | 24.02 | 9.61 | 11.04 | -114.07 |
| [Al][La] | 1:1 | 24.93 | 22.44 | 6.71 | 7.47 | -276.30 |
| [ATPPB][TEG] | 1:16 | 29.96 | 23.81 | 8.13 | 8.64 | -8.96 |
| [Be][La] | 1:1 | 34.19 | 28.01 | 10.18 | 11.84 | -391.44 |
| [ChCl][Fr] | 1:1 | 31.09 | 19.76 | 8.51 | 9.38 | -222.30 |
| [ChCl][Ma] | 1:1 | 29.84 | 19.92 | 8.16 | 7.38 | -215.60 |
| [Al][Ma] | 1:1 | 23.48 | 19.21 | 5.85 | 5.05 | -605.80 |
| [ChCl][MEA] | 1:5 | 37.79 | 32.46 | 12.33 | 18.63 | -25.99 |
| [ChCl][EA] | 1:7 | 38.22 | 32.89 | 12.51 | 18.87 | -32.79 |
| [TPACl][EA] | 1:7 | 36.56 | 35.43 | 11.76 | 18.90 | -6.57 |
| [ChCl][EG] | 1:2 | 31.83 | 22.42 | 9.24 | 11.16 | -70.24 |
| [MTPPBr][AC] | 1:4 | 30.96 | 33.61 | 9.82 | 8.32 | -185.19 |
| [TBABr][EA] | 1:7 | 37.01 | 37.15 | 12.07 | 19.70 | -15.71 |
| Chemical-based DES | | | | | | |
| [TBD][EG] | 1:4 | 29.04 | 24.39 | 8.12 | 10.44 | -123.05 |
| [DBU][EG] | 1:4 | 29.98 | 21.84 | 8.10 | 9.23 | -130.22 |
| [DBUH][Thy]/EG | 1:4 | 26.78 | 24.90 | 7.53 | 9.69 | -33.26 |
| [DBUH][Car]/EG | 1:4 | 26.33 | 24.35 | 7.34 | 9.35 | -50.27 |
| [DBN][BMIMCl][Im] | 1:1:2 | 35.36 | 46.54 | 13.34 | 19.62 | -471.29 |
| [TBD][BA] | 1:4 | 28.05 | 37.23 | 9.39 | 11.62 | -234.48 |
| [DBU][BA] | 1:4 | 29.02 | 33.88 | 9.40 | 10.58 | -187.76 |
| [DBN][BMIMCl][Im] | 1:1:1 | 34.49 | 44.81 | 12.33 | 19.15 | -525.20 |
| [DBU][Pyr] | 2:1 | 31.61 | 41.95 | 10.03 | 18.57 | -684.325 |
| [DBUH][Car]/EG | 1:3 | 25.85 | 25.41 | 7.28 | 9.77 | -45.36 |
| [DBUH][Thy]/EG | 1:3 | 26.36 | 26.04 | 7.49 | 10.15 | -12.95 |
| [DBN][BMIMCl][Im] | 1:2:1 | 37.04 | 45.78 | 13.30 | 19.35 | -389.84 |
| [ChCl][EA][Pz] | 1:7:1 | 37.79 | 33.94 | 12.39 | 19.22 | -33.34 |
| [MEACl][EDA] | 1:3 | 36.18 | 49.66 | 14.03 | 28.33 | -318.61 |
| [DBU][Eth] | 2:1 | 31.34 | 41.00 | 10.17 | 18.95 | -554.55 |
| [HMIMCl][EDA] | 1:4 | 35.75 | 59.93 | 14.78 | 29.55 | -407.64 |
| [DBU][Py] | 1:1 | 31.98 | 32.51 | 9.56 | 13.67 | -574.933 |
| [DBU][Oxa] | 2:1 | 34.71 | 31.42 | 10.40 | 12.92 | -404.603 |
| [HMIMCl][EDA] | 1:3 | 34.84 | 57.77 | 14.13 | 27.33 | -471.57 |
| [MEACl][AP] | 1:4 | 32.49 | 32.51 | 10.54 | 17.35 | -55.39 |

**References**

Aki, S.N., Mellein, B.R., Saurer, E.M., and Brennecke, J.F. (2004). High-pressure phase behavior of carbon dioxide with imidazolium-based ionic liquids. *The Journal of Physical Chemistry B* 108**,** 20355-20365.

Alcantara, M.L., Santos, J.P., Loreno, M., Ferreira, P.I., Paredes, M.L., Cardozo-Filho, L., Silva, A.K., Liao, L.M., Pires, C.A., and Mattedi, S. (2018). Low viscosity protic ionic liquid for CO_2_/CH_4_ separation: Thermophysical and high-pressure phase equilibria for diethylammonium butanoate. *Fluid Phase Equilibria* 459**,** 30-43.

Ali, E., Hadj-Kali, M.K., Mulyono, S., and Alnashef, I. (2016). Analysis of operating conditions for CO_2_ capturing process using deep eutectic solvents. *International Journal of Greenhouse Gas Control* 47**,** 342-350.

Ali, E., Hadj-Kali, M.K., Mulyono, S., Alnashef, I., Fakeeha, A., Mjalli, F., and Hayyan, A. (2014). Solubility of CO_2_ in deep eutectic solvents: experiments and modelling using the Peng–Robinson equation of state. *Chemical Engineering Research and Design* 92**,** 1898-1906.

Altamash, T., Amhamed, A., Aparicio, S., and Atilhan, M. (2020). Effect of hydrogen bond donors and acceptors on CO_2_ absorption by deep eutectic solvents. *Processes* 8**,** 1533.

Altamash, T., Haimour, T.S., Tarsad, M.A., Anaya, B., Ali, M.H., Aparicio, S., and Atilhan, M. (2017). Carbon dioxide solubility in phosphonium-, ammonium-, sulfonyl-, and pyrrolidinium-based ionic liquids and their mixtures at moderate pressures up to 10 bar. *Journal of Chemical & Engineering Data* 62**,** 1310-1317.

Chen, F.F., Huang, K., Fan, J.P., and Tao, D.J. (2018). Chemical solvent in chemical solvent: A class of hybrid materials for effective capture of CO_2_. *AIChE Journal* 64**,** 632-639.

Cui, G., Lv, M., and Yang, D. (2019). Efficient CO_2_ absorption by azolide-based deep eutectic solvents. *Chemical Communications* 55**,** 1426-1429.

Dai, C., Lei, Z., and Chen, B. (2017). Gas solubility in long‐chain imidazolium‐based ionic liquids. *AIChE Journal* 63**,** 1792-1798.

Francisco, M., Van Den Bruinhorst, A., Zubeir, L.F., Peters, C.J., and Kroon, M.C. (2013). A new low transition temperature mixture (LTTM) formed by choline chloride+ lactic acid: characterization as solvent for CO_2_ capture. *Fluid Phase Equilibria* 340**,** 77-84.

Fu, H., Hou, Y., Sang, H., Mu, T., Lin, X., Peng, Z., Li, P., and Liu, J. (2021a). Carbon dioxide capture by new DBU‐based DES: the relationship between ionicity and absorptive capacity. *AIChE Journal* 67**,** e17244.

Fu, H., Wang, X., Sang, H., Liu, J., Lin, X., and Zhang, L. (2021b). Highly efficient absorption of carbon dioxide by EG-assisted DBU-based deep eutectic solvents. *Journal of CO_2_ Utilization* 43**,** 101372.

García-Argüelles, S., Ferrer, M.L., Iglesias, M., Del Monte, F., and Gutiérrez, M.C. (2017). Study of superbase-based deep eutectic solvents as the catalyst in the chemical fixation of CO_2_ into cyclic carbonates under mild conditions. *Materials* 10**,** 759.

Ghaedi, H., Ayoub, M., Sufian, S., Shariff, A.M., Hailegiorgis, S.M., and Khan, S.N. (2017). CO_2_ capture with the help of phosphonium-based deep eutectic solvents. *Journal of Molecular Liquids* 243**,** 564-571.

Goodrich, B.F., De La Fuente, J.C., Gurkan, B.E., Lopez, Z.K., Price, E.A., Huang, Y., and Brennecke, J.F. (2011). Effect of water and temperature on absorption of CO_2_ by amine-functionalized anion-tethered ionic liquids. *The Journal of Physical Chemistry B* 115**,** 9140-9150.

Gu, Y., Hou, Y., Ren, S., Sun, Y., and Wu, W. (2020). Hydrophobic functional deep eutectic solvents used for efficient and reversible capture of CO_2_. *ACS omega* 5**,** 6809-6816.

Haider, M.B., Jha, D., Marriyappan Sivagnanam, B., and Kumar, R. (2018). Thermodynamic and kinetic studies of CO_2_ capture by glycol and amine-based deep eutectic solvents. *Journal of Chemical & Engineering Data* 63**,** 2671-2680.

Haider, M.B., and Kumar, R. (2020). Solubility of CO_2_ and CH_4_ in sterically hindered amine-based deep eutectic solvents. *Separation and Purification Technology* 248**,** 117055.

Hospital-Benito, D., Lemus, J., Moya, C., Santiago, R., and Palomar, J. (2020). Process analysis overview of ionic liquids on CO_2_ chemical capture. *Chemical Engineering Journal* 390**,** 124509.

Huang, K., and Peng, H.-L. (2017). Solubilities of Carbon Dioxide in 1-Ethyl-3-methylimidazolium Thiocyanate, 1-Ethyl-3-methylimidazolium Dicyanamide, and 1-Ethyl-3-methylimidazolium Tricyanomethanide at (298.2 to 373.2) K and (0 to 300.0) kPa. *Journal of Chemical & Engineering Data* 62**,** 4108-4116.

Huang, Y., Cui, G., Wang, H., Li, Z., and Wang, J. (2018). Tuning ionic liquids with imide-based anions for highly efficient CO_2_ capture through enhanced cooperations. *Journal of CO_2_ Utilization* 28**,** 299-305.

Huang, Y., Cui, G., Zhao, Y., Wang, H., Li, Z., Dai, S., and Wang, J. (2017). Preorganization and cooperation for highly efficient and reversible capture of low‐concentration CO_2_ by ionic liquids. *Angewandte Chemie International Edition* 56**,** 13293-13297.

Jalili, A.H., Mehdizadeh, A., Ahmadi, A.N., Zoghi, A.T., and Shokouhi, M. (2022). Solubility behavior of CO_2_ and H_2_S in 1-benzyl-3-methylimidazolium bis (trifluoromethylsulfonyl) imide ionic liquid. *The Journal of Chemical Thermodynamics***,** 106721.

Jalili, A.H., Mehrabi, M., Zoghi, A.T., Shokouhi, M., and Taheri, S.A. (2017). Solubility of carbon dioxide and hydrogen sulfide in the ionic liquid 1-butyl-3-methylimidazolium trifluoromethanesulfonate. *Fluid Phase Equilibria* 453**,** 1-12.

Jiang, B., Ma, J., Yang, N., Huang, Z., Zhang, N., Tantai, X., Sun, Y., and Zhang, L. (2019a). Superbase/acylamido-based deep eutectic solvents for multiple-site efficient CO_2_ absorption. *Energy & Fuels* 33**,** 7569-7577.

Jiang, Y.-Y., Wang, G.-N., Zhou, Z., Wu, Y.-T., Geng, J., and Zhang, Z.-B. (2008). Tetraalkylammonium amino acids as functionalized ionic liquids of low viscosity. *Chemical communications***,** 505-507.

Jiang, Y., Taheri, M., Yu, G., Zhu, J., and Lei, Z. (2019b). Experiments, modeling, and simulation of CO_2_ dehydration by ionic liquid, triethylene glycol, and their binary mixtures. *Industrial & Engineering Chemistry Research* 58**,** 15588-15597.

Jing, G., Qian, Y., Zhou, X., Lv, B., and Zhou, Z. (2018). Designing and screening of multi-amino-functionalized ionic liquid solution for CO_2_ capture by quantum chemical simulation. *ACS Sustainable Chemistry & Engineering* 6**,** 1182-1191.

Keller, A.N., Bentley, C.L., Morales-Collazo, O., and Brennecke, J.F. (2022). Design and characterization of aprotic N-heterocyclic anion ionic liquids for carbon capture. *Journal of Chemical & Engineering Data*.

Lee, Y.-Y., Penley, D., Klemm, A., Dean, W., and Gurkan, B. (2021). Deep eutectic solvent formed by imidazolium cyanopyrrolide and ethylene glycol for reactive CO_2_ separations. *ACS Sustainable Chemistry & Engineering* 9**,** 1090-1098.

Lei, X., Xu, Y., Zhu, L., and Wang, X. (2014). Highly efficient and reversible CO_2_ capture through 1, 1, 3, 3-tetramethylguanidinium imidazole ionic liquid. *RSC Advances* 4**,** 7052-7057.

Leron, R.B., Caparanga, A., and Li, M.-H. (2013). Carbon dioxide solubility in a deep eutectic solvent based on choline chloride and urea at T= 303.15–343.15 K and moderate pressures. *Journal of the Taiwan Institute of Chemical Engineers* 44**,** 879-885.

Leron, R.B., and Li, M.-H. (2013a). Solubility of carbon dioxide in a choline chloride–ethylene glycol based deep eutectic solvent. *Thermochimica acta* 551**,** 14-19.

Leron, R.B., and Li, M.-H. (2013b). Solubility of carbon dioxide in a eutectic mixture of choline chloride and glycerol at moderate pressures. *The Journal of Chemical Thermodynamics* 57**,** 131-136.

Li, F., Bai, Y., Zeng, S., Liang, X., Wang, H., Huo, F., and Zhang, X. (2019). Protic ionic liquids with low viscosity for efficient and reversible capture of carbon dioxide. *International Journal of Greenhouse Gas Control* 90**,** 102801.

Li, G., Deng, D., Chen, Y., Shan, H., and Ai, N. (2014). Solubilities and thermodynamic properties of CO_2_ in choline-chloride based deep eutectic solvents. *The Journal of Chemical Thermodynamics* 75**,** 58-62.

Li, X., Hou, M., Han, B., Wang, X., and Zou, L. (2008). Solubility of CO_2_ in a choline chloride+ urea eutectic mixture. *Journal of Chemical & Engineering Data* 53**,** 548-550.

Liu, Y.-M., Tian, Z., Qu, F., Zhou, Y., Liu, Y., and Tao, D.-J. (2019). Tuning ion-pair interaction in cuprous-based protic ionic liquids for significantly improved CO capture. *ACS Sustainable Chemistry & Engineering* 7**,** 11894-11900.

Lu, M., Han, G., Jiang, Y., Zhang, X., Deng, D., and Ai, N. (2015). Solubilities of carbon dioxide in the eutectic mixture of levulinic acid (or furfuryl alcohol) and choline chloride. *The Journal of Chemical Thermodynamics* 88**,** 72-77.

Luo, X.-Y., Chen, X.-Y., Qiu, R.-X., Pei, B.-Y., Wei, Y., Hu, M., Lin, J.-Q., Zhang, J.-Y., and Luo, G.-G. (2019a). Enhanced CO_2_ capture by reducing cation–anion interactions in hydroxyl-pyridine anion-based ionic liquids. *Dalton Transactions* 48**,** 2300-2307.

Luo, X.Y., Lv, X.Y., Shi, G.L., Meng, Q., Li, H.R., and Wang, C.M. (2019b). Designing amino‐based ionic liquids for improved carbon capture: one amine binds two CO_2_. *AIChE Journal* 65**,** 230-238.

Meng, Y., Wang, X., Zhang, F., Zhang, Z., and Wu, Y. (2018). IL-DMEE nonwater system for CO_2_ capture: absorption performance and mechanism investigations. *Energy & Fuels* 32**,** 8587-8593.

Min, Z., Li, Z., Wang, H., Xuan, X., Zhao, Y., and Wang, J. (2021). How does the moisture affect CO_2_ absorption by a glycinate ionic liquid? *ACS Sustainable Chemistry & Engineering* 9**,** 853-862.

Mukesh, C., Khokarale, S.G., Virtanen, P., and Mikkola, J.-P. (2019). Rapid desorption of CO_2_ from deep eutectic solvents based on polyamines at lower temperatures: an alternative technology with industrial potential. *Sustainable Energy & Fuels* 3**,** 2125-2134.

Mulia, K., Putri, S., Krisanti, E., and Nasruddin (Year). "Natural deep eutectic solvents (NADES) as green solvents for carbon dioxide capture", in: *AIP Conference Proceedings*: AIP Publishing LLC), 020022.

Niedermaier, I., Bahlmann, M., Papp, C., Kolbeck, C., Wei, W., Krick CalderóN, S., Grabau, M., Schulz, P.S., Wasserscheid, P., and SteinrüCk, H.-P. (2014). Carbon dioxide capture by an amine functionalized ionic liquid: fundamental differences of surface and bulk behavior. *Journal of the American Chemical Society* 136**,** 436-441.

Noorani, N., and Mehrdad, A. (2021). Experimental and theoretical study of CO_2_ sorption in biocompatible and biodegradable cholinium-based ionic liquids. *Separation and Purification Technology* 254**,** 117609.

Peng, L., Wu, W., Hou, Y., Li, K., and Zhang, H. (2021). Experimental investigation on solubility characteristics of CO_2_–ionic liquids as new working pairs for absorption refrigeration systems. *Journal of Engineering Thermophysics* 30**,** 330-339.

Pishro, K.A., Murshid, G., Mjalli, F.S., and Naser, J. (2021). Carbon dioxide solubility in amine-based deep eutectic solvents: experimental and theoretical investigation. *Journal of Molecular Liquids* 325**,** 115133.

Qian, W., Hao, J., Zhu, M., Sun, P., Zhang, K., Wang, X., and Xu, X. (2022). Development of green solvents for efficient post-combustion CO_2_ capture with good regeneration performance. *Journal of CO_2_ Utilization* 59**,** 101955.

Qu, Y., Lan, J., Chen, Y., and Sun, J. (2021). Amino acid ionic liquids as efficient catalysts for CO_2_ capture and chemical conversion with epoxides under metal/halogen/cocatalyst/solvent-free conditions. *Sustainable Energy & Fuels* 5**,** 2494-2503.

Ren, H., Lian, S., Wang, X., Zhang, Y., and Duan, E. (2018). Exploiting the hydrophilic role of natural deep eutectic solvents for greening CO_2_ capture. *Journal of Cleaner Production* 193**,** 802-810.

Ren, S., Hou, Y., Tian, S., Chen, X., and Wu, W. (2013). What are functional ionic liquids for the absorption of acidic gases? *The Journal of Physical Chemistry B* 117**,** 2482-2486.

Safarov, J., Abdullayeva, G., Bashirov, M., Tuma, D., and Bashirov, R. (2021). The ionic liquid 1-ethyl-3-methylimidazolium methanesulfonate revisited: solubility of carbon dioxide over an extended range of temperature and pressure. *Journal of Molecular Liquids* 333**,** 115920.

Santos, E., Albo, J., Rosatella, A., Afonso, C.A., and Irabien, A. (2014). Synthesis and characterization of magnetic ionic liquids (MILs) for CO_2_ separation. *Journal of Chemical Technology & Biotechnology* 89**,** 866-871.

Saravanamurugan, S., Kunov‐Kruse, A.J., Fehrmann, R., and Riisager, A. (2014). Amine‐functionalized amino acid‐based ionic liquids as efficient and high‐capacity absorbents for CO_2_. *ChemSusChem* 7**,** 897-902.

Sarmad, S., Nikjoo, D., and Mikkola, J.-P. (2020). Amine functionalized deep eutectic solvent for CO_2_ capture: Measurements and modeling. *Journal of Molecular Liquids* 309**,** 113159.

Sarmad, S., Xie, Y., Mikkola, J.-P., and Ji, X. (2017). Screening of deep eutectic solvents (DESs) as green CO_2_ sorbents: from solubility to viscosity. *New Journal of Chemistry* 41**,** 290-301.

Seo, S., Desilva, M.A., Xia, H., and Brennecke, J.F. (2015). Effect of cation on physical properties and CO_2_ solubility for phosphonium-based ionic liquids with 2-cyanopyrrolide anions. *The Journal of Physical Chemistry B* 119**,** 11807-11814.

Shahrom, M.S.R., Wilfred, C.D., Macfarlane, D.R., Vijayraghavan, R., and Chong, F.K. (2019). Amino acid based poly (ionic liquid) materials for CO_2_ capture: effect of anion. *Journal of Molecular Liquids* 276**,** 644-652.

Sharma, P., Do Park, S., Park, K.T., Nam, S.C., Jeong, S.K., Yoon, Y.I., and Baek, I.H. (2012). Solubility of carbon dioxide in amine-functionalized ionic liquids: Role of the anions. *Chemical engineering journal* 193**,** 267-275.

Shukla, S.K., and Mikkola, J.-P. (2018). Intermolecular interactions upon carbon dioxide capture in deep-eutectic solvents. *Physical Chemistry Chemical Physics* 20**,** 24591-24601.

Siani, G., Tiecco, M., Di Profio, P., Guernelli, S., Fontana, A., Ciulla, M., and Canale, V. (2020). Physical absorption of CO_2_ in betaine/carboxylic acid-based natural deep eutectic solvents. *Journal of Molecular Liquids* 315**,** 113708.

Song, T., Avelar Bonilla, G.M., Morales-Collazo, O., Lubben, M.J., and Brennecke, J.F. (2019). Recyclability of encapsulated ionic liquids for post-combustion CO_2_ capture. *Industrial & Engineering Chemistry Research* 58**,** 4997-5007.

Taheri, M., Dai, C., and Lei, Z. (2018). CO_2_ capture by methanol, ionic liquid, and their binary mixtures: Experiments, modeling, and process simulation. *AIChE Journal* 64**,** 2168-2180.

Thapaliya, B.P., Puskar, N.G., Slaymaker, S., Feider, N.O., Do-Thanh, C.-L., Schott, J.A., Jiang, D.-E., Teague, C.M., Mahurin, S.M., and Dai, S. (2021). Synthesis and characterization of macrocyclic ionic liquids for CO_2_ separation. *Industrial & Engineering Chemistry Research* 60**,** 8218-8226.

Trivedi, T.J., Lee, J.H., Lee, H.J., Jeong, Y.K., and Choi, J.W. (2016). Deep eutectic solvents as attractive media for CO_2_ capture. *Green Chemistry* 18**,** 2834-2842.

Uma Maheswari, A., and Palanivelu, K. (2015). Carbon dioxide capture and utilization by alkanolamines in deep eutectic solvent medium. *Industrial & Engineering Chemistry Research* 54**,** 11383-11392.

Vijayaraghavan, R., Oncsik, T., Mitschke, B., and Macfarlane, D. (2018). Base-rich diamino protic ionic liquid mixtures for enhanced CO_2_ capture. *Separation and Purification Technology* 196**,** 27-31.

Wang, C., Luo, H., Jiang, D.E., Li, H., and Dai, S. (2010). Carbon dioxide capture by superbase‐derived protic ionic liquids. *Angewandte Chemie* 122**,** 6114-6117.

Wang, L., Xie, H., Huang, X., Xu, Y., Chu, T., and Wu, Z. (2020). CO_2_ and CH_4_ sorption by solid-state ammonium and imidazolium ionic liquids. *Energy & Fuels* 35**,** 599-609.

Wang, X., Wu, C., and Yang, D. (2021a). CO_2_ absorption mechanism by diamino protic ionic liquids (DPILs) containing azolide anions. *Processes* 9**,** 1023.

Wang, Z., Wang, Z., Chen, J., Wu, C., and Yang, D. (2021b). The Influence of hydrogen bond donors on the CO_2_ absorption mechanism by the bio-phenol-based deep eutectic solvents. *Molecules* 26**,** 7167.

Wang, Z., Wang, Z., Xin, H., Yang, D., Wu, C., and Chen, J. (2022). Deep eutectic solvents composed of bio-phenol-derived superbase ionic liquids and ethylene glycol for CO_2_ capture. *Chemical Communications*.

Wu, J., Lv, B., Wu, X., Zhou, Z., and Jing, G. (2019). Aprotic heterocyclic anion-based dual-functionalized ionic liquid solutions for efficient CO_2_ uptake: quantum chemistry calculation and experimental research. *ACS Sustainable Chemistry & Engineering* 7**,** 7312-7323.

Wu, W., Wang, L., Li, X., Liu, H., Zhang, H., and Dou, B. (2021). Phase equilibrium characteristics of CO_2_ and ionic liquids with [FAP]− anion used for absorption-compression refrigeration working pairs. *Journal of Thermal Science* 30**,** 165-176.

Xie, Y., Dong, H., Zhang, S., Lu, X., and Ji, X. (2016). Solubilities of CO_2_, CH_4_, H_2_, CO and N_2_ in choline chloride/urea. *Green Energy & Environment* 1**,** 195-200.

Yan, H., Zhao, L., Bai, Y., Li, F., Dong, H., Wang, H., Zhang, X., and Zeng, S. (2020). Superbase ionic liquid-based deep eutectic solvents for improving CO_2_ absorption. *ACS Sustainable Chemistry & Engineering* 8**,** 2523-2530.

Yan, M., Huan, Q., Zhang, Y., Fang, W., Chen, F., Pariatamby, A., Kanchanatip, E., and Wibowo, H. (2022). Effect of operating parameters on CO_2_ capture from biogas with choline chloride—monoethanolamine deep eutectic solvent and its aqueous solution. *Biomass Conversion and Biorefinery***,** 1-15.

Yim, J.-H., Seo, W.-W., and Lim, J.S. (2021). CO_2_ solubility in bis (trifluoromethylsulfonyl) imide ([Tf_2_N]) anion-based ionic liquids:[BVIM][Tf_2_N],[P_4441_][Tf_2_N], and [N_4222_][Tf_2_N]. *Journal of Chemical & Engineering Data* 67**,** 3-13.

Yu, H., Wu, Y.-T., Jiang, Y.-Y., Zhou, Z., and Zhang, Z.-B. (2009). Low viscosity amino acid ionic liquids with asymmetric tetraalkylammonium cations for fast absorption of CO_2_. *New Journal of Chemistry* 33**,** 2385-2390.

Zema, Z.A., Chen, T., Shu, H., and Xu, Y. (2021). Tuning the CO_2_ absorption and physicochemical properties of K+ chelated dual functional ionic liquids by changing the structure of primary alkanolamine ligands. *Journal of Molecular Liquids* 344**,** 117983.

Zhang, K., Hou, Y., Wang, Y., Wang, K., Ren, S., and Wu, W. (2018). Efficient and reversible absorption of CO_2_ by functional deep eutectic solvents. *Energy & Fuels* 32**,** 7727-7733.

Zhang, N., Huang, Z., Zhang, H., Ma, J., Jiang, B., and Zhang, L. (2019a). Highly efficient and reversible CO_2_ capture by task-specific deep eutectic solvents. *Industrial & Engineering Chemistry Research* 58**,** 13321-13329.

Zhang, Y., Wu, Z., Chen, S., Yu, P., and Luo, Y. (2013). CO_2_ capture by imidazolate-based ionic liquids: effect of functionalized cation and dication. *Industrial & Engineering Chemistry Research* 52**,** 6069-6075.

Zhang, Z., Zhang, L., He, L., Yuan, W.-L., Xu, D., and Tao, G.-H. (2019b). Is it always chemical when amino groups come across CO_2_? anion–anion-interaction-induced inhibition of chemical adsorption. *The Journal of Physical Chemistry B* 123**,** 6536-6542.

Zhao, T., Zhang, X., Tu, Z., Wu, Y., and Hu, X. (2018). Low-viscous diamino protic ionic liquids with fluorine-substituted phenolic anions for improving CO_2_ reversible capture. *Journal of Molecular Liquids* 268**,** 617-624.

Zhao, Y., Yu, B., Yang, Z., Zhang, H., Hao, L., Gao, X., and Liu, Z. (2014). A protic ionic liquid catalyzes CO_2_ conversion at atmospheric pressure and room temperature: synthesis of quinazoline‐2, 4 (1H, 3H)‐diones. *Angewandte Chemie International Edition* 53**,** 5922-5925.

Zubeir, L.F., Van Osch, D.J., Rocha, M.A., Banat, F., and Kroon, M.C. (2018). Carbon dioxide solubilities in decanoic acid-based hydrophobic deep eutectic solvents. *Journal of Chemical & Engineering Data* 63**,** 913-919.
